# AS‐TLC‐Based High‐Throughput Screening Integrated with CuAAC Optimization Strategy Facilitates the Rapid Discovery of YTHDC1 Inhibitor

**DOI:** 10.1002/advs.77386

**Published:** 2026-08-27

**Authors:** Mingchen Wang, Shuiping Fu, Ye Xu, Jia Gao, Jiwei Ren, Xu Yang, Hesong Xu, Ge Sun, Yanjie Li, Yanlin Wang, Lu Jin, Peijuan Chen, Ying Xu, Jianhao Li, Hua Lin, Chujiao Hu, Kaixian Chen, Huan Xiong, Cheng Luo, Shijie Chen

**Affiliations:** ^1^ School of Pharmaceutical Science and Technology Hangzhou Institute for Advanced Study University of Chinese Academy of Sciences Hangzhou China; ^2^ Precision Medicine Research Institute of Guizhou and Guizhou Provincial Key Laboratory of Digestive System Diseases The Affiliated Hospital of Guizhou Medical University Guizhou Medical University Guiyang China; ^3^ Innovation Center for AI and Drug Discovery School of Pharmacy East China Normal University Shanghai China; ^4^ State Key Laboratory of Drug Research Shanghai Institute of Materia Medica Chinese Academy of Sciences Shanghai China; ^5^ University of Chinese Academy of Sciences Beijing China; ^6^ Zhongshan Institute for Drug Discovery Shanghai Institute of Materia Medica Chinese Academy of Sciences Zhongshan China; ^7^ Key Laboratory of Microbial Pathogenesis and Interventions of Fujian Province University the Key Laboratory of Innate Immune Biology of Fujian Province Biomedical Research Center of South China College of Life Sciences Fujian Normal University Fuzhou China; ^8^ Tianfu Jincheng Laboratory Chengdu China

**Keywords:** affinity selection thin‐layer chromatography, click chemistry, drug discovery, RNA recognition, YTHDC1

## Abstract

The classical sequential workflow—individual compound screening, followed by stepwise design‐synthesis‐purification of derivatives—makes lead compound discovery time‐consuming and cost‐prohibitive. Herein, we developed Affinity Selection Thin‐Layer Chromatography (AS‐TLC) as a method for high‐throughput screening (HTS), enabling efficient and cost‐effective screening of compound mixtures. Furthermore, we integrated activity assays with AS‐TLC to minimize target protein consumption and lower the false positive rate, through which we identified a YTH domain‐containing protein 1 (YTHDC1) inhibitor, fragment YD, with a half‐maximal inhibitory concentration (IC_50_) of 20.01 ± 3.30 µm. Building on this hit, we applied copper(I)‐catalyzed azide–alkyne cycloaddition (CuAAC) click chemistry for rapid structural modification and compound library construction, ultimately obtaining LC‐YD03 with significantly enhanced inhibitory activity (IC_50_ = 41.98 ± 6.24 nm). The interaction mode between LC‐YD03 and YTHDC1 was further clarified via X‐ray crystallographic analysis. This study demonstrates that the AS‐TLC‐activity assay platform enables efficient hit screening from compound mixtures; subsequent click chemistry‐based combinatorial library technology accelerates lead compound optimization. Collectively, this workflow significantly reduces the time and cost associated with lead compound discovery.

## Graphical abstract

We developed Affinity Selection Thin‐Layer Chromatography (AS‐TLC) as a method for high‐throughput screening (HTS) of compound mixtures. Furthermore, we integrated activity assays with AS‐TLC and identified a YTH domain‐containing protein 1 (YTHDC1) inhibitor. Building on this hit, we applied copper(I)‐catalyzed azide–alkyne cycloaddition (CuAAC) click chemistry for rapid structural modification and compound library construction, ultimately obtaining LC‐YD03 with significantly enhanced inhibitory activity.

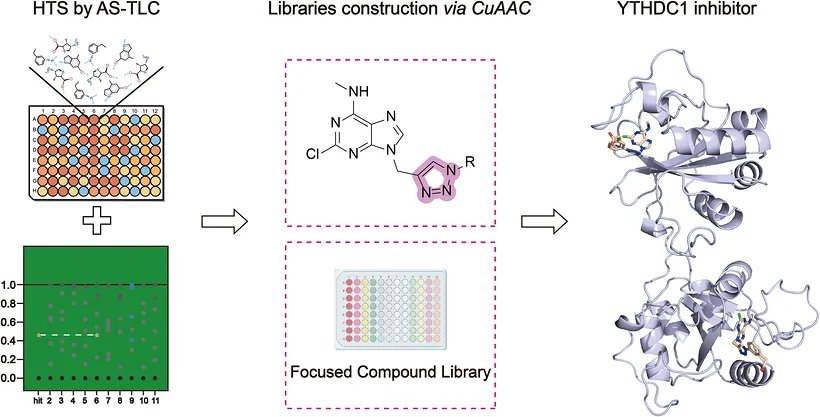

## 1 Introduction

High‐throughput screening (HTS) serves as a pivotal tool for lead discovery. A canonical approach for hit identification involves utilizing automated instrumentation to conduct exhaustive individual compound screening, yet this method often relies on expensive equipment and reagents, rendering HTS costly. More recently, some innovative strategies have emerged, such as Affinity Selection Mass Spectrometry (AS‐MS) [1], DNA‐encoded chemical library (DEL) [2, 3], and nuclear magnetic resonance (NMR)‐based screening of combinatorial compound libraries [4]. Unlike traditional “one‐well‐one‐compound” processes, these strategies use mixtures of compounds, thereby lowering screening costs and improving screening efficacy.

These advantages have rendered such screening approaches increasingly prominent, and they are playing an increasingly important role, especially in emerging drug discovery and the development of agents targeting difficult‐to‐drug proteins. AS‐MS enables direct screening of crude reaction mixtures and natural extracts, facilitating the rapid mining of molecular glues and G protein‐coupled receptor [5, 6] ligands. DEL, integrated with chemical biology, covalent modification, and artificial intelligence [7], constructs large‐scale mixed libraries to efficiently identify potent and selective inhibitors against undruggable targets [8]. NMR, relying on saturation transfer difference, water‐ligand observed via gradient spectroscopy and diffusion‐ordered spectroscopy label‐free techniques coupled with multi‐dimensional NMR and intelligent algorithms, can screen fragment libraries, recognize weak‐affinity molecules, and assemble high‐affinity compounds, with wide applications in antitumor lead discovery [9, 10]. However, despite these advantages, AS‐MS relies heavily on mass spectrometry detection; DEL analysis requires next‐generation sequencing, and its DNA tags impose restrictions on applicable chemical synthesis routes; while NMR‐based approaches suffer from difficult deconvolution of complex mixtures and high technical barriers regarding instrumentation and operation. As a result, the utility of these processes is limited in many laboratories.

Thin‐layer chromatography (TLC) is a separation technique that utilizes a liquid solvent (mobile phase) to separate the components of a chemical mixture across the surface of an adsorbent material (stationary phase) [11, 12]. Compounds are determined by R*_f_* values (the ratio of the distance from the sample origin to the center of the sample spot to the distance from the sample origin to the solvent front after development) and colors under ultraviolet (UV) light or after reaction with a chromogenic agent. Notably, a previous study has reported the application of TLC in bioautography for detecting compound activity [13]. With its simplicity, reliability, and sensitivity in qualitative analysis, mixture separation, and compound identification, TLC holds potential as a method for screening hit compounds from chemical mixtures.

Here, we propose an Affinity Selection Thin‐Layer Chromatography (AS‐TLC) strategy for high‐throughput screening of mixed‐compound. In our method, a mixture of compounds with known structures is first incubated with the protein of interest (i.e., the target protein). Potential hit compounds that interact with the target protein are then separated from the chemical mix using size‐exclusion chromatography (SEC). A subsequent denaturation step releases the hit compounds from the target protein, which are then analyzed alongside the original mixture on the same TLC plate. The hit compounds are identified based on their R*_f_* values and colors under UV.

We demonstrated the feasibility of AS‐TLC using three targets, ubiquitin carboxyl‐terminal hydrolase 7 (USP7), chromatin remodeling regulator CECR2 (CECR2) and cyclin‐dependent kinase 2 (CDK2) with their positive compounds. However, our results showed two limitations: protein consumption that requires further reduction (currently 18–36 nmol per 1000 compounds), and unavoidable false positives that lack functional activity. We therefore developed a two‐stage strategy pairing AS‐TLC with target‐specific activity assays—screening compound mixtures by activity first, then resolving and confirming hits by AS‐TLC. Validated on USP7 and CDK2, it reduced protein use to 0.6–3 nmol per 1000 compounds and excluded a non‐inhibitory false positive for CDK2.

YTH domain‐containing protein 1 (YTHDC1), an N⁶‐methyladenosine (m^6^A)‐reader target whose narrow binding pocket hinders small‐molecule discovery, was particularly challenging [14]. We thus integrated homogeneous time‐resolved fluorescence (HTRF), the activity assay designed to characterize the binding of YTHDC1 to m^6^A ribonucleic acid (RNA), with AS‐TLC, and screened 14 560 compounds to identify fragment YD with ahalf‐maximal inhibitory concentration (IC_50_) of 20.01 ± 3.30 µM. Structure‐guided click‐chemistry libraries (1636 derivatives) then yielded LC‐YD03 (IC_50_ = 41.98 ± 6.24 nM)—a 476‐fold gain over fragment YD and 4.2‐fold over positive control compound **25** [15]—whose co‐crystal structure with YTHDC1 revealed the key binding interactions.

## 2 Results and Discussion

### 2.1 Design of the AS‐TLC Method for Identifying Target‐Binding Compounds

**FIGURE 1:**
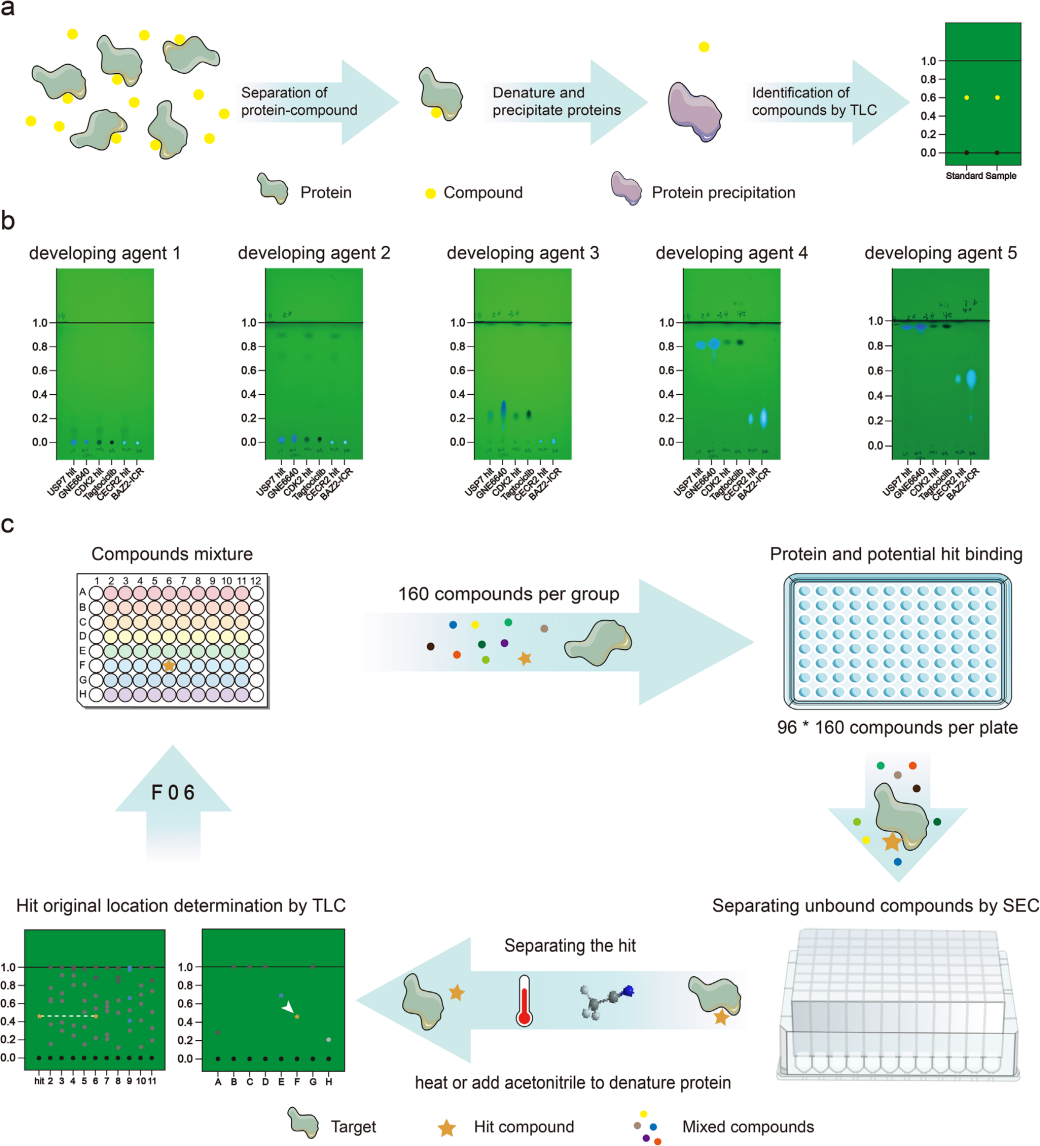
Development of the AS‐TLC HTS method. (a) Schematic diagram of the AS‐TLC detection method based on compound binding with target. (b) Identification of compounds binding to targets by TLC. Positive compounds of USP7, CDK2, and CECR2 were identified by TLC. Developing agent 1: petroleum ether: ethyl acetate (5:1, v/v); Developing agent 2: petroleum ether: ethyl acetate (1:1, v/v); Developing agent 3: 100% ethyl acetate; Developing agent 4: ethyl acetate: methanol (4:1, v/v); Developing agent 5: ethyl acetate: methanol (3:2, v/v). TLC plates were observed under 254 nm ultraviolet light. (c) Schematic diagram of AS‐TLC HTS method.

First, we sought to use TLC to identify hit compounds that bind to target proteins. The process began with incubating the target protein with a mixture of compounds to allow binding, after which protein‐compound complexes were separated from non‐binding compounds using SEC [16]. The target protein was then denatured through heating or by treatment with organic reagents to release the bound compounds and then subjected to TLC (Figure 1a). Given that this method relies on the binding affinity between targets and compounds, we term it “Affinity Selection Thin‐Layer Chromatography (AS‐TLC)”.

To verify whether AS‐TLC could detect compounds that bind to proteins, USP7, CDK2, and CECR2 were incubated with their respective positive compounds GNE‐6640 [17] and USP7‐IN‐8 [18], Tagtociclib [19] and AT7519 [20], GNE‐375 [21] and BAZ2‐ICR [22], with a protein concentration of 25 µm and each compound at 50 µM. After incubation at room temperature for 30 min, non‐binding compounds were excluded by SEC (the desalting step). The compound‐target complexes were then denatured to release the enriched compounds, and the latter were subjected to TLC, with measurements taken against standards. This method successfully identified the positive compounds for each of the three targets (Figure 1b), confirming that AS‐TLC is a suitable workflow for detecting compound‐target interactions. On this basis, we further explored the application of AS‐TLC in high‐throughput screening, aiming to pick out hit compounds from mixed compounds.

### 2.2 Establish AS‐TLC HTS Method for Mixed Compounds Screening

Under consistent individual compound concentrations, the detected quantity of hits decreases as the total compounds increases. Specifically, mixing scales exceeding 320 compounds show significantly reduced detection yields compared to scale of 80 or 160 compounds (Figure S1a,b). Then, we compared the accuracy of tracing a “hit” from 40, 80, and 160 compounds in one mixture, respectively. Here, “hit” refers to the compound selected randomly to simulate the hit compound in a real screening situation. “Hit” compound and mixture of each scale were analyzed on the same TLC plate. Mixtures were divided into 5/10 dispensing groups, adapted to the size of the TLC plate. Then, UV light was used to determine which subgroup the “hit” came from by comparing the R*_f_* values and the colors. The results showed the successful identification of “hits” across all three different mixtures. Meanwhile, increasing the number of mixed compounds raises the likelihood of encountering compounds with similar R*_f_* values, colors, or spot shapes to hit compounds, thereby reducing TLC analysis accuracy (Figure S1c). To achieve the highest possible screening throughput while ensuring detection accuracy, we chose a mixing scale of 160 compounds.

Next, we determined the method of mixing 160 compounds to allow for the subsequent identification of the position and structure of the hit within the compound library. Compounds in our laboratory were stored in 96‐well plates at 10 mm in dimethyl sulfoxide (DMSO), with each plate comprising 80 compounds (in columns 2–11, rows A–H). We constructed a mixture of 160 compounds via two steps. First, we aspirated 1 µL of each compound from within the same column for each of the two plates, and combined the compounds into one well of a new plate. This resulted in 10 (columns 2–11) mixtures of 16 compounds. Second, we took 8 µL of the 16‐compound mixture from columns 2–11 in the same row in plate 3 and combined them into a well in the first column of plate 3 to obtain a mixture of 160 compounds (Figure S1d). All compounds from original plates 1 and 2 were mixed to give this 160 compounds mixture.

We used this strategy to mix the USP7‐positive compound GNE‐6640 into the 160‐compound mixture to verify whether GNE‐6640 could be identified from the mixture. We then sought to verify the specific well from which the hit compound originated, which was achieved based on the R*_f_* value and color by UV detection. Through this process, we identified that the hit compound originated from well H06 on plate 2—exactly the position where the positive compound GNE‐6640 was initially placed (Figure S1e). These results validated the successful identification of the positive compound from a mix of 160 compounds.

In our assay, mixed compounds needed to be sufficiently separated and displayed in TLC for efficient recognition. Since HTS generally uses a large number of diverse compounds, we prepared five developing solvents of different polarities: (1) petroleum ether: ethyl acetate (5:1, v/v); (2) petroleum ether: ethyl acetate (1:1, v/v); (3) 100% ethyl acetate; (4) ethyl acetate: methanol (4:1, v/v); (5) ethyl acetate: methanol (3:2, v/v). Compound mixtures were separated sequentially on the TLC plate using these various solvents until the hit compound could be distinguished (with the R*_f_* value of the hit compound ranging between 0.2 and 0.8) (Figure 1b and Figure S1f). Moreover, we sought to choose a non‐destructive and broad‐spectrum visualization method for compounds. In TLC, most compounds can be visualized non‐destructively using UV light at 365 and 254 nm. To verify this, we randomly selected batches of 40, 80, 160, and 320 compounds, respectively, with three replicate groups for each batch size, from a total of 20 000 in‐house compounds. All samples were detected under UV light at 365 and 254 nm. The average UV‐detectable ratios (mean ± standard deviation) for the four batch sizes were 92.5 ± 0.0%, 92.1 ± 1.9%, 90.8 ± 2.4% and 93.3 ± 3.2%, respectively. In total, 1800 compounds were assayed, yielding an overall UV detection rate of 92.4% (Figure S1g).

To explore the general working concentration range of the protein, we performed assays using the bromodomain PHD finger transcription factor (BPTF) protein. Two positive compounds against this target, DC‐BPi‐01 (IC_50_ = 7.8 µm) and DC‐BPi‐10 (IC_50_ = 14.7 µm), were selected [23]. With the compound concentration fixed at 50 µm, the protein concentration was set from 10 to 50 µm. The detection results revealed that obvious binding between BPTF and the positive compounds was observed when the protein concentration was 20 µm or higher (Figure S1h,i). For high‐throughput screening, a protein working concentration of 50 µm was adopted to ensure the capture of potential weakly active compounds with K_d_ values ranging from 50 to 100 µm.

In summary, we established the AS‐TLC high‐throughput screening method: (1) Construct a mixed compounds library, with each pool containing 160 compounds, then incubate target proteins (a working concentration of 50 µm is recommended) with a mix of compounds (at a concentration of 50 µM per individual compound); (2) Separate the non‐bound compounds using a high‐throughput desalting plate; (3) Denature the compound‐target complexes by heating (100°C, 1 min) or through the use of organic solvents (300 µL acetonitrile); and (4) Identify the hit compounds within the supernatant using TLC (detected under 254 and 365 nm ultraviolet light) (Figure 1c).

### 2.3 Validation of AS‐TLC HTS Method using USP7, CDK2, and CECR2 and their Reported Inhibitors

To further validate the feasibility of AS‐TLC method across various targets, we selected USP7, CDK2, and CECR2 to conduct small‐scale screening. For each target, we randomly chose 800 compounds from 10 plates of our in‐house compound library and mixed them with two positive compounds of each protein with different activities into five groups (Figure S2a–c), with each group containing 160 compounds. The concentration of a single compound is 50 µm, and the concentration of the protein is 25 µm. Our analysis identified two positive compounds for USP7 in groups 2 and 4, positioned at well E06 on plate 3 (GNE‐6640) and well F07 on plate 8 (USP7‐IN‐8) (Figure S2d). Similarly, we accurately identified positive compounds for CDK2 at well B05 on plate 4 (Tagtociclib) and well E03 on plate 8 (AT7519), as well as for CECR2 at well B10 on plate 2 (GNE‐375) and well E06 on plate 10 (BAZ2‐ICR) (Figure S2e,f). These results validate the utility of the AS‐TLC method to identify hit compound through HTS. We further determined the Z’‐factor of AS‐TLC using the corresponding hit compounds for these three targets in both single‐compound and mixed‐compound systems, which verified the stability of the experimental system (Z’‐factor > 0.5) (Figure S3).

While these initial results were encouraging, we noted that the method required a high protein concentration (25 µm), leading to significant protein usage in large‐scale screening applications. Notably, alongside validated CDK2 inhibitors, a preliminary hit (164‐A02) showed target binding (Figures S2e and S4a) but no kinase inhibition (Figure S4b,c), highlighting that affinity‐based screening alone would yield false positives. To address this, we integrated activity assays with AS‐TLC to reduce protein demand and false‐positive rates. Based on whether activity assays use probes, we categorized the approach into two types: (1) AS‐TLC combined with a fluorescent probe‐dependent activity assay and (2) AS‐TLC combined with a fluorescent probe‐independent activity assay (Figure S5).

### 2.4 Establishment and Validation of AS‐TLC Combined with Fluorescent Probe‐Dependent Activity Assays

We first explored the integration of AS‐TLC with fluorescent probe‐dependent activity assays, selecting USP7 and CDK2 as model enzymes for this development.

For USP7, ubiquitin‐rhodamine is a substrate analog commonly used as a fluorescent probe in its enzymatic assays [24, 25]. USP7 cleaves ubiquitin to release rhodamine, which emits fluorescence under 365 nm UV excitation. We thus designed the combined assay as follows: compound mixtures were incubated with 250 nM USP7 and 500 nM ubiquitin‐rhodamine at 37°C for 60 min, after which the reaction solution was spotted onto a TLC plate. Rhodamine was separated using an ethyl acetate: methanol (4:1, v/v) solvent, where a decrease in its fluorescence intensity indicated the presence of USP7 inhibitors. Subsequently, AS‐TLC enabled isolation and identification of hit compounds (Figure S6a).

To validate this approach, we randomly divided 160 compounds mixture into two groups, one containing GNE‐6640 and the other without, and observed significant differences in rhodamine fluorescence (Figure S6b). Further, we screened 8 groups of 160 compounds via the USP7 enzyme activity assay, identified one group with significant inhibitory activity, and through subsequent AS‐TLC analysis, correctly pinpointed the USP7 positive inhibitor GNE‐6640 (Figure S6c). These results confirmed the feasibility of the method for USP7.

For targets lacking available fluorescent probes, attaching fluorophores to the natural substrate or positive compounds offers a solution. For CDK2, we synthesized a fluorescent probe (CPJ‐4‐1, Supporting Information) by conjugating fluorescein isothiocyanate (FITC) to the CDK2 positive inhibitor PF‐06873600 [26] (Figure S7). The probe was incubated with CDK2, and then complexes were isolated via SEC, with their fluorescence detectable under UV light on TLC plates. We selected 1 µM CPJ‐4‐1 and 2 µM CDK2 for analysis with the compound mixture, hypothesizing that compounds competing with the probe for CDK2 binding would reduce the probe's fluorescence on the TLC plate—an observation that would trigger further AS‐TLC analysis of that specific mixture (Figure S8a). First, we added the positive inhibitor Tagtociclib to 5 randomly selected groups of 160 compound mixtures, which resulted in a significant reduction in fluorescence intensity (Figure S8b). We then screened 8 sets of 160 compound mixtures (totaling 1280 compounds), which included the false positive compound 164‐A02 that had been identified via standalone AS‐TLC screening. Through this screening, we precisely identified the positive compound Tagtociclib (Figure S8c). Notably, the false positive compound 164‐A02 was no longer detected in the integrated screening. Additionally, the protein usage for USP7 and CDK2 protein was only 0.21 and 0.18 mg, respectively—approximately tenfold lower than the 2.0 and 1.5 mg required for standalone AS‐TLC screening at the same scale.

Collectively, these results demonstrated that AS‐TLC can be effectively combined with probe‐based activity detection methods for high‐efficiency screening of compound mixtures.

### 2.5 Establishment of AS‐TLC Combined with Fluorescent Probe‐Independent Activity Assay and its Application in YTHDC1 Screening

While the combination of AS‐TLC with fluorescent probe‐dependent activity assays proved effective, many targets lack natural substrates or reported positive compounds that can be easily modified with fluorophores. To address this limitation, we sought to integrate AS‐TLC with an alternative activity detection assay that does not rely on fluorescent probes, and applied this strategy to YTHDC1 screening. We proposed integrating AS‐TLC with the existing HTRF assay system, aiming to reduce reagent consumption of the HTRF kits, mitigate false positive screening results, and enhance screening efficiency for rapid identification of valid hit compounds.

**FIGURE 2:**
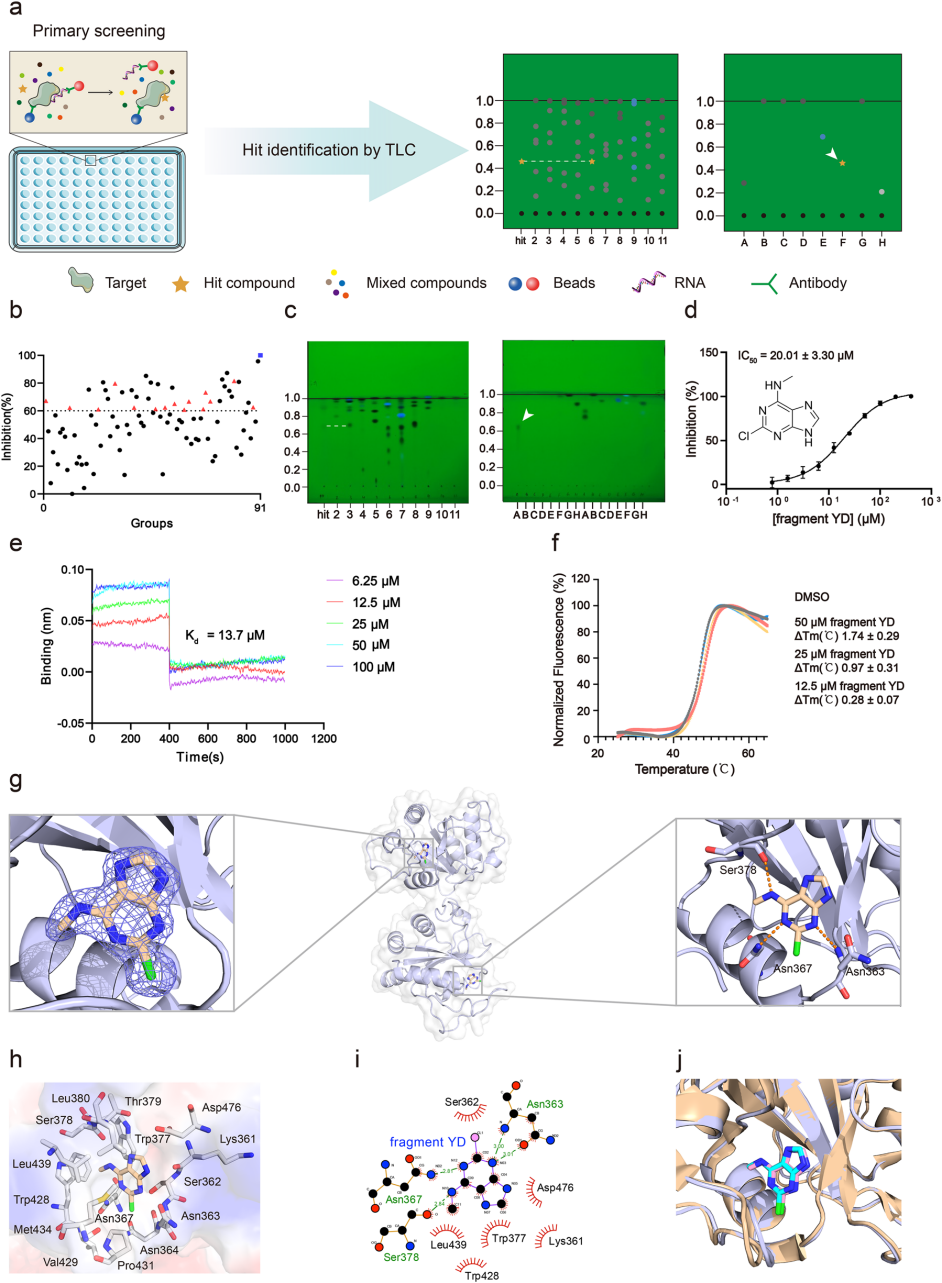
Development and application of the HTRF assay combined AS‐TLC method for YTHDC1. (a) Schematic design of the combined HTRF and AS‐TLC screening assay for YTHDC1. (b) HTRF‐based screening results of compound mixtures. Red dots indicate candidate mixtures, and a blue dot indicates a mixture containing the positive compound. (c) Identification of the hit compound fragment YD from candidate mixtures via AS‐TLC. White dotted lines connect standard compounds in the mixture that match fragment YD in terms of R*_f_* value and color. White arrow points to fragment YD. (d) AlphaScreen assay assessment of YTHDC1 activity upon treatment with increasing concentrations of fragment YD. Data are presented as mean ± SD from three independent experiments. (e) Dissociation constants (K_d_) of fragment YD binding to YTHDC1 determined via biolayer interferometry (BLI) experiments. (f) Effects of fragment YD on thermal stability of the YTHDC1‐YTH domain protein (referred to as YTHDC1 for brevity, unless specified otherwise) assessed by TSA (n = 3). A representative melting curve is shown. (g) Electron density map (contoured at 1.2 σ) and binding mode of fragment YD in complex with YTHDC1 (PDB ID: 9VS9). Orange dashed lines indicate hydrogen bonds. (h) Residues within a 5‐Å radius of fragment YD in the YTHDC1 binding pocket (PDB ID: 9VS9). (i) Interaction diagram of fragment YD with YTHDC1 generated using LigPlot+ (v.2.2), depicting hydrogen bonds (green dashed lines) and hydrophobic contacts (red arcs). (j) Structural superposition of the YTHDC1‐fragment YD complex (YTHDC1 in light purple, fragment YD in pink, PDB ID: 9VS9) and the compound **1**‐bound structure (YTHDC1 in wheat, compound **1** in cyan, PDB ID: 7P88).

Hit compounds in the mixture should suppress the fluorescence ratio, and such hits could then be further identified by AS‐TLC (Figure 2a). To establish this assay, we selected compound **25** as the positive compound of YTHDC1 and added it to one of 16 groups mixed compounds, each containing 160 compounds. Using HTRF, the mixture with compound **25** showed a significantly reduced fluorescence ratio compared to the control group without compound **25** (Figure S9a). Subsequent AS‐TLC analysis successfully identified compound **25** from the mixture (Figure S9b), confirming the feasibility of this probe‐independent combined approach.

Building on this, we proceeded to conduct high‐throughput screening of YTHDC1 and verification of identified compounds. We screened 91 groups of 160 mixed compounds (totaling 14560 individual compounds) against YTHDC1. After eliminating compounds with fluorescence interference, 16 groups with inhibition above 60% (fluorescence ratios below 0.9) were selected for repetition (Figure 2b, Table S1). Among these, 6 groups maintained consistently low fluorescence ratios and were subjected to further AS‐TLC analysis to identify potential hits (Figure S9c). To identify potentially weak‐affinity hit compounds, we used a higher protein concentration (50 µM) than that employed in the aforementioned AS‐TLC assays (25 µM) of CECR2, USP7, and CDK2 earlier in this study. One of these 6 groups, which showed the strongest inhibitory activity, was found to contain the compound **25** (Figure S9d), while the remaining 5 groups exhibited slightly reduced inhibition.

Subsequent AS‐TLC analysis of these 5 groups identified hits from 2 groups: 125‐A03 (named fragment YD) (Figure S9e and Figure 2c) and 168‐G04 (Figure S9f). To verify the accuracy of its identification, we incubated YTHDC1 with graded concentrations of 168‐G04 standard and confirmed the recovered compound exhibited identical R*_f_* values to the standard (Figure S9g). To validate the activity of fragment YD and 168‐G04 and their binding to YTHDC1, we used AlphaScreen and biolayer interferometry (BLI) assays, respectively. The AlphaScreen assay showed fragment YD had an IC_50_ of 20.01 ± 3.30 µM (Figure 2d), while 168‐G04 exhibited substantially weaker activity (IC_50_ > 100 µM) (Figure S9h). BLI assays corroborated these findings, with binding dissociation constants (K_d_) of 13.7 µM for fragment YD and 71.8 µM for 168‐G04 (Figure 2e and Figure S9i).

For the remaining 3 groups identified via activity assays, AS‐TLC recovered no compounds—likely false positives eliminated by AS‐TLC assay. To verify, 480 individual compounds from these 3 groups underwent HTRF screening at 25 µM. After excluding compound fluorescence interference, three compounds with inhibition rates exceeding 70% were identified (Figure S9j). However, BLI showed all had K_d_ greater than 100 µM against YTHDC1 (Figure S9k–m), and AlphaScreen assays confirmed they had almost no inhibitory activity (Figure S9n). These results validate that AS‐TLC correctly recovered no compounds from these 3 groups, further demonstrating the integrated AS‐TLC‐activity approach helps effectively reduce false positives. Moreover, compared to screening individual compounds sequentially, our current strategy cut the amount of the HTRF reagent kit used to less than 1/100, significantly lowering reagent consumption.

Through these screening and validation steps, we ultimately identified fragment YD as a YTHDC1 inhibitor, which we further characterized using thermal shift assay (TSA) to confirm direct binding. At a 10:1 molar ratio of fragment YD to protein, the melting temperature (Tm) of YTHDC1 increased by 1.74°C. This shift decreased in a concentration‐dependent manner, confirming that fragment YD binds to YTHDC1 and enhances its thermal stability (Figure 2f). Notably, fragment YD is structurally identical to compound **1**, previously reported by Zálešák and coworkers [27], which further validates the reliability of our AS‐TLC screening approach.

Structural insights into the binding mechanism were obtained by co‐crystallizing fragment YD with YTHDC1, yielding a high‐resolution (1.82 Å) structure (PDB ID: 9VS9) via X‐ray crystallography. The crystal structure revealed that fragment YD occupies the substrate‐binding pocket of YTHDC1, specifically within the aromatic cage formed by Trp377, Trp428, and Leu439 (Figure 2g). Primary interactions involve van der Waals contacts between the N^6^‐methyl group of fragment YD and the hydrophobic cavity, along with key hydrogen bonds: between the 6‐amino group of fragment YD and Ser378 (2.84 Å), and between the 1‐ and 3‐nitrogen atoms of fragment YD and Asn367 (2.81 Å) and Asn363 (3.00 Å, 3.01 Å), respectively (Figure 2h,i). Superimposition of our YTHDC1‐fragment YD complex with the structure reported by Zálešák et al. revealed a high degree of structural alignment [27], reinforcing the consistency and validating the binding mode of this inhibitor (Figure 2j). Given this structural consistency and the compound's weak activity, further modification and optimization of fragment YD are needed. These structural insights provide a robust foundation for rational optimization of YTHDC1‐selective inhibitors with improved potency, specificity, and distinct structural features.

### 2.6 Design and Construction of the ‘Direct‐to‐Biology’ Focused Library Via Combinatorial Library Technology

**FIGURE 3:**
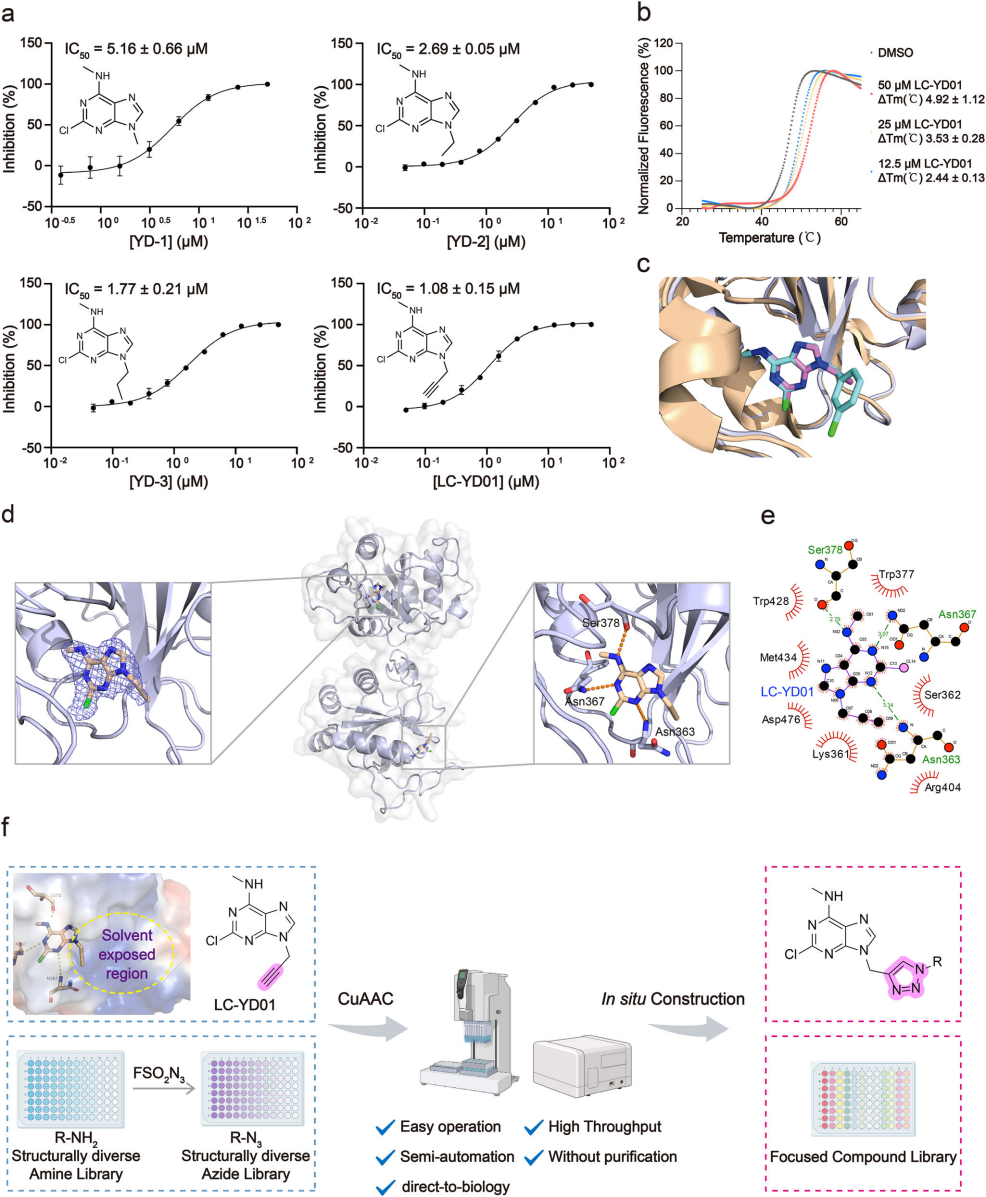
Preliminary chemical modification and library design based on fragment YD. (a) YTHDC1 was treated with increasing concentrations of compounds YD‐1, YD‐2, YD‐3, and LC‐YD01 and activity of protein was assessed using the AlphaScreen assay. Data are presented as mean ± SD from three independent experiments. (b) TSA assays evaluating the stabilizing effect of LC‐YD01 on YTHDC1 protein. Data represent mean ± SD from three replicates. (c) Structural superposition of the YTHDC1‐LC‐YD01 complex (YTHDC1 in light purple, LC‐YD01 in violet, PDB ID: 9VRA) and the compound **25**‐bound structure (YTHDC1 in wheat, compound **25** in aquamarine, PDB ID: 8Q4M). (d) Binding mode of LC‐YD01 in complex with YTHDC1 determined via X‐ray crystallography (PDB ID: 9VRA). Orange dashed lines denote hydrogen bonds. (e) Interaction profile of LC‐YD01 with residues in the YTHDC1 binding pocket, generated using LigPlot+ (v.2.2), illustrating hydrogen bonds (green dashes) and hydrophobic interactions (red arcs). (f) High‐throughput construction of focused libraries via single‐substrate diversified CuAAC.

Based on the observation of essentially identical binding poses of fragment YD and YTHDC1 (Figure 2j), we hypothesized that N^9^‐alkylation of the purine scaffold to make interactions outside the aromatic cage would be preserved across different fragments. The preliminary exploration revealed the validity of this strategy. As shown in Figure 3a, the introduction of alkyl chains of varying length (methyl, ethyl, propyl) exhibited enhanced potency (YD‐1, IC_50_ = 5.16 ± 0.66 µM; YD‐2, IC_50_ = 2.69 ± 0.05 µM; YD‐3, IC_50_ = 1.77 ± 0.21 µM) (Figure 3a) over the parent compound fragment YD. To accelerate insights into structure‐activity relationships (SARs) and ultimately a speed‐up of the whole lead optimization process, we then utilized combinatorial library technology to design and construct the ‘direct‐to‐biology’ focused library. The replacement of the propyl group of YD‐3 with a propargyl moiety, yielding click chemistry‐compatible derivative LC‐YD01 (Figure 3a). This modification preserves the core scaffold while incorporating a bioorthogonal handle (alkyne) for efficient conjugation with azide‐functionalized fragments. Notably, LC‐YD01 exhibited acceptable inhibitory potency against YTHDC1 (IC_50_ = 1.08 ± 0.15 µm, Figure 3a), and TSA assays demonstrated concentration‐dependent manner (Figure 3b).

We also resolved the co‐crystal structure of LC‐YD01 with YTHDC1 at 2.49 Å resolution (PDB ID: 9VRA). The crystal structure of the obtained complex revealed the presence of two copies of YTHDC1. For consistency, all subsequent structural analyses of the complex were performed using Chain B. As expected, structural alignment of this co‐crystal structure of LC‐YD01 and the benzyl substituent of compound **25** exhibited identical spatial orientations, providing experimental validation of our structural hypothesis (Figure 3c). As anticipated, the purine core of LC‐YD01 maintained key hydrogen‐bond interactions with Asn363, Asn367, and Ser378, mirroring the binding pattern observed for fragment YD (Figure 3d). The propargyl group extended near the solvent‐exposed region, engaging in hydrophobic interactions with Lys361, Ser362, and Asn363. Additionally, the overall hydrophobic contacts between LC‐YD01 and YTHDC1 were significantly enhanced (Figure 3e). These findings confirmed the primary rationale of the N^9^‐alkylation of the purine scaffold and further guided the focused compound library construction.

**FIGURE 4:**
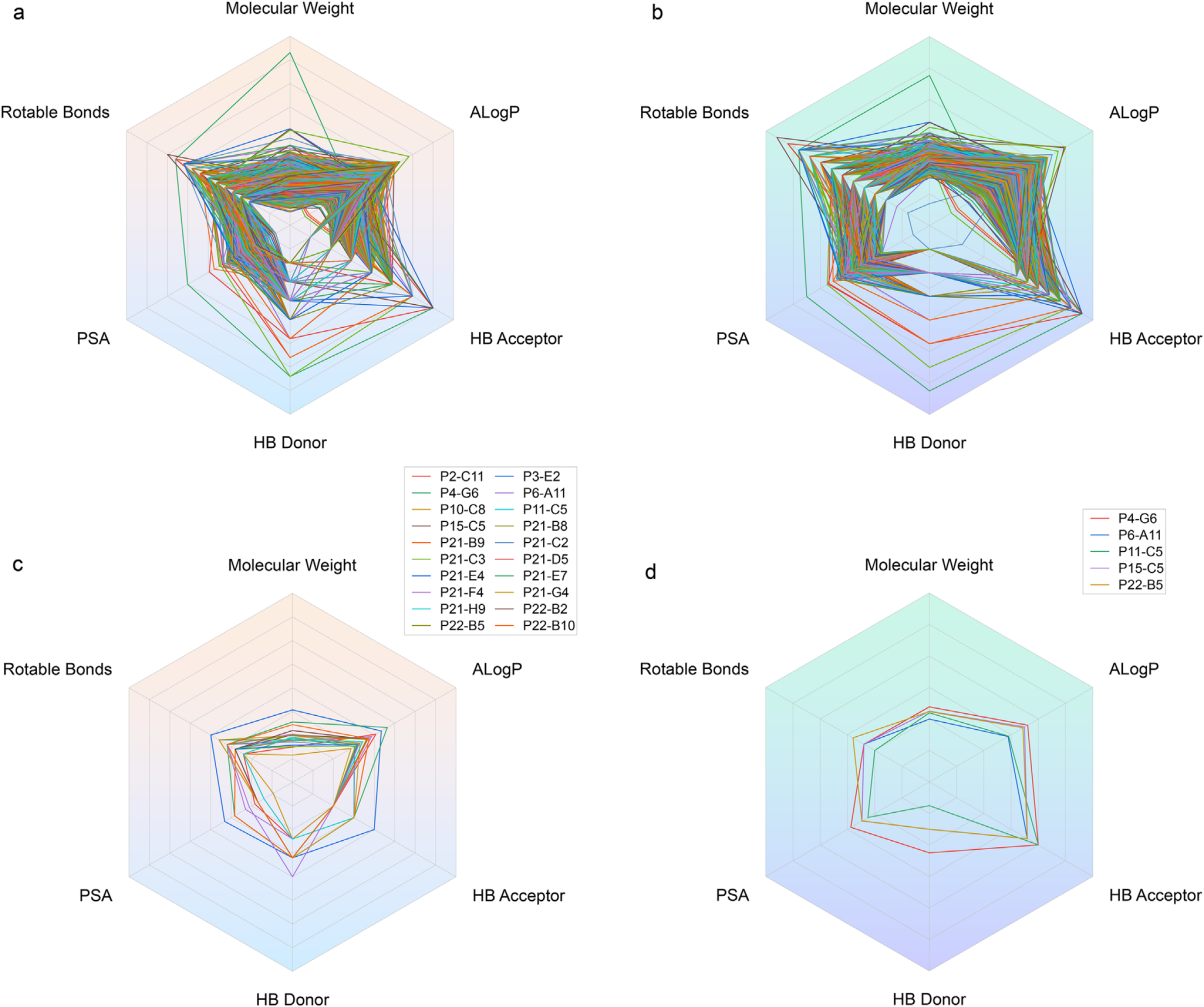
The chemical properties of azide building blocks and corresponding triazole compounds. (a) The coverage of above‐mentioned calculated properties of all azide building blocks; (b) The coverage of above‐mentioned calculated properties of all azides derived corresponding triazole compounds; (c) The screened azide fragments properties of P2‐C11, P3‐E2, P4‐G6, P6‐A11, P10‐C8, P11‐C5, P15‐C5, P21‐B8, P21‐B9, P21‐C2, P21‐C3, P21‐D5, P21‐E4, P21‐E7, P21‐F4, P21‐G4, P21‐H9, P22‐B5 and P22‐B10, highlighted using indicated colors. (d) The properties of the screened azide‐derived corresponding triazole compounds P4‐G6, P6‐A11, P11‐C5, P15‐C5, and P22‐B5, highlighted using the indicated colors. The calculated properties were visualized by Origin using a radar chart.

We next executed copper‐catalyzed azide‐alkyne cycloaddition (CuAAC) to rapidly assemble a focused 1636‐member triazole library (named Library YD) for SAR profiling (Figure 3f). Our previous work has demonstrated a microplate‐based parallel compound library approach that introduced a 1360‐member primary amine fragment library [28]. Inspired by Meng et al.’s landmark work [29] inventing the diazotizing reagent fluorosulfuryl azide (FSO_2_N_3_), which enabled the transformation of azides from primary amines with high yields, we expanded our in‐house primary amine fragment library to a total of 1636 and used the reagent to obtain the azide library. To obtain the most comprehensive SAR information, this azide library considered various properties of azide fragments, including molecular weight (MW), ALogP, hydrogen bond donors and acceptors (HBD and HBA), polar surface area (PSA), and rotatable bond counts (Figure 4a). The Library YD was then constructed based on the azide library and LC‐YD01 via a CuAAC reaction, yielding triazole derivatives of the purine scaffold with diverse characteristics (Figure 4b). The incorporation of a pipetting workstation and microplate oscillators ensured minimal manual intervention and high reproducibility. Compatibility with biological assays enabled a ‘direct‐to‐biology’ profile and led to in situ high‐throughput screening.

The detailed library construction process is provided in Supporting Information. The progression of the reaction was monitored using ultra‐performance liquid chromatography‐mass spectrometry (UPLC‐MS) to confirm the formation of triazole product. From the compound library, 498 crude samples were analyzed, and their conversion efficiencies were quantified as percentages, which can be found in Table S2 and Figure S10. Twenty representative compounds were selected for detailed profiling. These compounds exhibited distinct chromatographic peaks and mass spectral patterns, as shown in Figure S11.

### 2.7 Rapid Identification of the Potent YTHDC1 Inhibitor LC‐YD03 from Library YD

We designed and synthesized a FITC‐conjugated probe (Figure S12a) based on positive control compound **25** and developed a fluorescence polarization (FP) assay for rapid primary screening of Library YD (Figure S12b,c). To rapidly identify bioactive compounds, the in situ constructed compound library of 1636 compounds (theoretical concentration of each library compound = 10 mm) was diluted to 5 µm with DMSO after pre‐testing and tested for their YTHDC1 inhibitory activity without further purification. The initial data are presented as the YTHDC1 inhibition rate in Tables S3–S5. We selected 20 compounds (identified by their position in the microtiter plate well) exhibiting > 95% inhibition against YTHDC1, synthesized and purified each individually on a milligram scale for their inhibitory potency at four concentrations (5, 2.5, 1.25, and 0.625 µm, respectively) (Table 1).

**TABLE 1:** The inhibition rate of the 20 re‐synthesized compounds against YTHDC1.

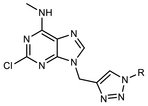					
Compd.	R	Inhibition Rate [%]			
		C = 5 µM	C = 2.5 µM	C = 1.25 µm	C = 0.625 µm
P2‐C11	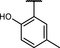	84.13 ± 1.09	79.70 ± 1.63	70.14 ± 2.10	44.78 ± 1.94
P3‐E2	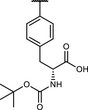	91.65 ± 0.47	85.24 ± 0.42	73.54 ± 1.17	58.93 ± 1.07
P4‐G6 (LC‐YD02)	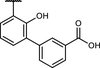	97.56 ± 0.62	92.27 ± 1.07	85.12 ± 0.98	72.64 ± 1.73
P6‐A11 (LC‐YD03)	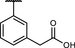	94.56 ± 0.43	89.00 ± 0.80	79.98 ± 1.16	69.03 ± 0.69
P11‐C5 (LC‐YD04)	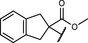	93.40 ± 0.65	88.25 ± 0.80	80.16 ± 0.68	66.72 ± 1.82
P15‐C5 (LC‐YD05)	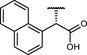	94.57 ± 0.27	89.68 ± 0.82	82.51 ± 1.75	70.17 ± 2.93
P21‐B4	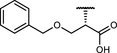	81.69 ± 1.62	69.21 ± 1.46	50.01 ± 1.38	34.41 ± 2.78
P21‐B8	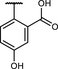	87.96 ± 0.36	79.59 ± 0.90	67.33 ± 1.83	52.02 ± 2.28
P21‐B9	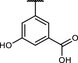	91.23 ± 0.44	85.17 ± 1.39	71.87 ± 0.73	53.61 ± 2.86
P21‐C2	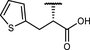	85.68 ± 1.35	77.03 ± 0.75	64.26 ± 1.09	47.98 ± 1.38
P21‐C3	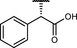	89.86 ± 1.00	84.14 ± 2.40	69.28 ± 0.60	52.08 ± 2.88
P21‐D5	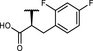	84.66 ± 1.09	74.70 ± 1.99	59.70 ± 0.54	40.67 ± 2.26
P21‐E4	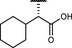	82.07 ± 1.00	71.18 ± 0.52	57.45 ± 1.68	39.97 ± 1.94
P21‐E7	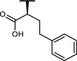	86.21 ± 1.59	75.88± 0.51	61.12 ± 0.75	44.58 ± 2.79
P21‐F4	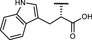	86.06 ± 0.44	75.88 ± 1.16	60.51 ± 1.53	42.99 ± 0.69
P21‐G4	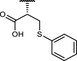	88.04 ± 0.77	77.94 ± 0.74	64.26 ± 0.29	47.86 ± 2.18
P21‐H9	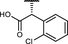	89.04 ± 0.24	82.17 ± 1.01	70.84 ± 0.37	56.07 ± 0.95
P22‐B2	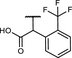	91.51 ± 1.00	85.20 ± 0.92	74.28 ± 0.58	59.85 ± 2.50
P22‐B5 (LC‐YD06)	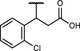	95.94 ± 0.83	92.32 ± 0.61	82.57 ± 0.94	71.95 ± 1.75
P22‐B10	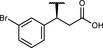	89.81 ± 1.02	82.11 ± 1.63	67.35 ± 2.97	51.55 ± 1.68
compound **25**	—	81.12 ± 1.41	75.36 ± 4.80	50.66 ± 2.32	30.01 ± 1.39

Promising YTHDC1 inhibitory activity was exhibited by all 20 of the screened compounds in a concentration‐dependent manner (Table 1). Interestingly, these screened azide fragments covered only a small fraction of the entire chemical space, which was defined by specific parameters: HBD ranging from 1 to 3, HBA ranging from 2 to 4, PSA ranging from 35.25 to 124.39, and ALogP values between 0.37 and 3.69 (Figure 4c). Apart from P21‐E4, most of these compounds contain aromatic rings, primarily benzene rings, which are either directly linked to the triazole or connected by short carbon chains. It is noteworthy that almost all of the screened compounds contain a carboxyl group, with the exceptions of P2‐C11 and P11‐C5 (Table 1). This suggests a high probability of special ion–ion or dipole–ion interactions forming between the negatively charged carboxyl group and the positively charged residues. The direct attachment of the carboxyl moiety to a phenyl ring was observed in compounds P4‐G6 (LC‐YD02), P21‐B8 and P21‐B9. These compounds exhibited moderate to high inhibition rates (C = 0.625 µm: 72.64%, 52.02%, and 53.61%, respectively) against YTHDC1. The rigidity of the aromatic rings may stabilize bioactive conformations and enhance hydrophobic interactions, thereby enabling sustained target inhibition. The rest compounds that contain α/β‐aliphatic linkers linking the carboxyl group to aromatic rings showed fascinating properties. Compounds in which the α‐C of the carboxyl group is directly connected to the aromatic ring (e.g., P15‐C5, P21‐C3, P21‐H9, and P22‐B2) showed a relatively higher YTHDC1 inhibition rate than compounds in which the α‐C of the carboxyl is not directly connected to the aromatic ring (e.g., P10‐C8, P21‐C2, P21‐D5, P21‐F4, and P21‐G4). Compounds in which the β‐C of the carboxyl group is directly connected to a halogen‐substituted benzene ring, such as P22‐B5 (LC‐YD06) and P22‐B10, also exhibited strong potency against YTHDC1 (C = 0.625 µM: 71.95% and 51.55%, respectively).

**FIGURE 5:**
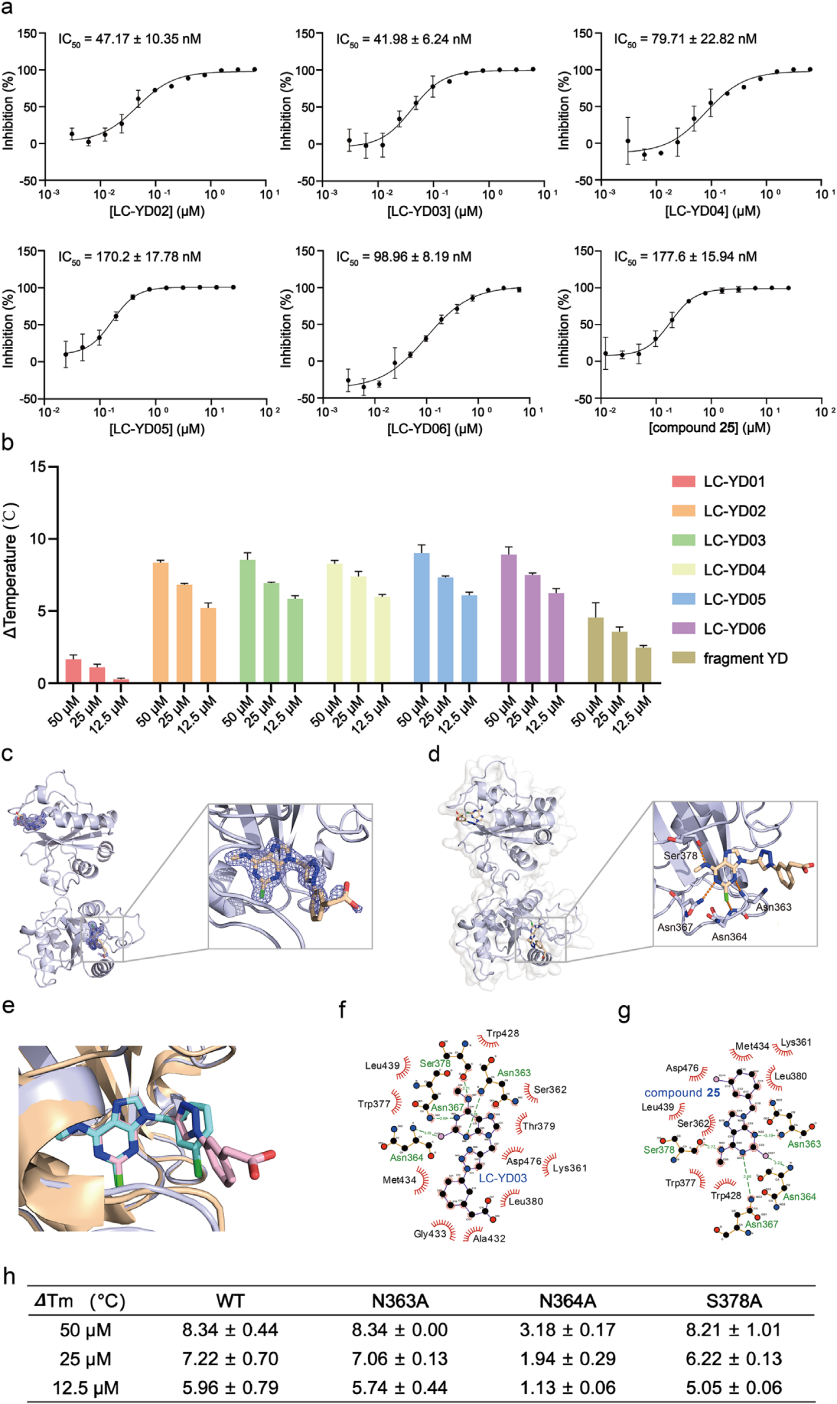
Identification of YTHDC1 inhibitor LC‐YD03. (a) AlphaScreen assay assessment of YTHDC1 activity upon treatment with increasing concentrations of compounds LC‐YD02 to LC‐YD06 and the positive control (compound **25**). Data represent mean ± SD from three biologically independent experiments. (b) TSA assays evaluating the thermal stabilization effects of LC‐YD02‐LC‐YD06 and compound **25** on the YTHDC1 protein. Data represent mean ± SD of triplicate measurements from three independent experiments. (c) Electron density map (contoured at 1.2 σ) of LC‐YD03 bound to YTHDC1 (PDB ID: 9VSB). (d) Binding mode of LC‐YD03 in the YTHDC1 pocket, determined via X‐ray crystallography (PDB ID: 9VSB). Orange dashed lines indicate hydrogen‐bonding interactions. (e) Structural superposition of the YTHDC1‐LC‐YD03 complex (YTHDC1 in light purple, LC‐YD03 in light pink, PDB ID: 9VSB) and the compound **25**‐bound structure (YTHDC1 in wheat, compound **25** in aquamarine, PDB ID: 8Q4M). (f) Ligand interaction diagram of LC‐YD03 with YTHDC1, generated using LigPlot+ (v.2.2), depicting hydrogen bonds (green dashes) and hydrophobic contacts (red arcs). (g) Interaction profile of compound **25** with residues in the YTHDC1 binding pocket (LigPlot+ v.2.2). (h) Effects of LC‐YD03 on the thermal stability of YTHDC1(WT), YTHDC1(N363A), YTHDC1(N364A), and YTHDC1(S378A) proteins measured by TSA (n = 3).

Five compounds (LC‐YD02 to LC‐YD06) that exhibited high YTHDC1 inhibition rates (> 60%) at the concentration of 0.625 µm were selected to determine their half‐maximal inhibitory concentration (IC_50_) via AlphaScreen assays against YTHDC1 (Figure 5a). These compounds were more potent than the positive control compound **25**, with distinct properties: HBD ranged from 1 to 3, HBA ranged from 9 to 10, PSA ranged from 112.64 to 143.87, and ALogP values between 1.78 and 3.22 (Figure 4d).

In particular, compound LC‐YD03 showed the most promising potency (IC_50_ = 41.98 ± 6.24 nm)—476‐fold higher than hit fragment YD (IC_50_ = 20.01 ± 3.30 µm) and 4.2‐fold higher than the positive control compound **25** (IC_50_ = 177.6 ± 15.93 nm) (Figure 5a). BLI analysis determined the dissociation constants (K_d_) of YTHDC1 with LC‐YD03 and compound **25** to be 102 and 189 nm, respectively (Figure S13a,b). Further TSA experiments confirmed the strong binding of these optimized compounds. For example, 50 µM of LC‐YD03 increased YTHDC1's melting temperature by 8.6°C (Figure 5b). Simultaneously, LC‐YD03 demonstrates selectivity towards YTHDC1 among YTH family proteins. In the FP assay, LC‐YD03 showed nearly complete inhibition of YTHDC1 at 50 µm, whereas its inhibition of YTHDF1, YTHDF2, YTHDF3, and YTHDC2 was much weaker (Figure S14a). In the AlphaScreen assay, LC‐YD03 also displayed preferential inhibition of YTHDC1 over YTHDF1, YTHDF2, and YTHDF3 (Figure S14b). We further examined whether LC‐YD03 affects the m^6^A writer complex METTL3/METTL14. The positive control STM2457 [30] showed clear inhibition with an IC_50_ of 171.3 nm, confirming the reliability of the assay. In contrast, LC‐YD03 didn't reach 50% inhibition within the tested concentration range, with IC_50_ values greater than 25 µm, indicating no obvious inhibition of METTL3/METTL14 under these conditions (Figure S14c).

Since LC‐YD03 contains a purine‐linked triazole scaffold, which has been used in kinase inhibitor development [31], we further performed kinase profiling against 41 representative kinases at 1000 nm. LC‐YD03 showed no obvious inhibition of any tested kinase, with all inhibition rates below 50%; the highest inhibition was only 13.4% against MET (Figure S15a,b). Taken together, these data demonstrate that LC‐YD03 has favorable selectivity toward YTHDC1 and shows no apparent off‐target activity against METTL3/METTL14 or the tested kinase panel.

To gain a deeper understanding of the binding mechanism of LC‐YD03 and to explain its improved activity compared to fragment YD, as well as its excellent performance in high‐throughput screening, we resolved the co‐crystal structure of LC‐YD03 with YTHDC1 at a resolution of 1.80 Å (Figure 5c). The structure revealed that, in addition to the conserved hydrogen bonds between the purine core and Asn363, Asn367, and Ser378 (Figure 5d), the overall binding mode of LC‐YD03 is highly similar to that of compound **25**, as confirmed by structural superimposition (Figure 5e). Furthermore, a detailed comparison uncovered that LC‐YD03 forms an additional hydrogen bond via its purine chlorine atom to Asn364 (Figure 5f). Notably, the hydrogen bond between the 6‐amino group and Ser378 is strengthened (2.71 versus 2.84 Å in fragment YD), and LC‐YD03 exhibits enhanced hydrophobic interactions compared to LC‐YD01. These structural improvements collectively contributed to the significant boost in potency. In contrast to compound **25** (Figure 5g), LC‐YD03 engages in enhanced interactions through the meta‐substituted phenylacetic acid moiety attached to the triazole ring, notably with Leu380, Gly433, and Ala432—a finding that explains why the majority of compounds from the high‐throughput screening contain a carboxyl group. The phenyl ring serves as the core for hydrophobic interactions, and the carboxyl group of LC‐YD03 is linked to the phenyl ring via a methylene spacer. This also explains why compounds that contain a carboxyl group but have the carboxyl group located farther from the benzene ring (e.g., P21‐B4, P21‐E7, and P21‐F4), or even lack a benzene ring entirely (e.g., P21‐C2 and P21‐E4), exhibit weaker activity than LC‐YD03. In LC‐YD03, the carboxyl group is attached to the meta position of the phenyl ring via a methylene linker. This positioning allows both the phenyl ring and the carboxyl group to embed into a hydrophobic subpocket formed by residues such as Trp428, Leu380, Gly433, and Ala432, thereby generating stronger van der Waals interactions. Consequently, LC‐YD03 stands out among other carboxyl‐containing compounds. To functionally validate these structural observations, we generated YTHDC1 mutants YTHDC1(N363A), YTHDC1(N364A), YTHDC1(N367A), and YTHDC1(S378A) and evaluated them by TSA, FP, and AlphaScreen assays. TSA analysis showed that LC‐YD03 strongly stabilized YTHDC1(wild‐type, WT), YTHDC1(N363A), and YTHDC1(S378A), whereas the thermal shift was markedly reduced for YTHDC1(N364A). At 50 µm LC‐YD03, the ΔTm values of YTHDC1(WT), YTHDC1(N363A), YTHDC1(S378A), and YTHDC1(N364A) were 8.34 ± 0.44°C, 8.34 ± 0.00°C, 8.21 ± 1.01°C, and 3.18 ± 0.17°C, respectively (Figure 5h). FP assays showed that YTHDC1(N364A) failed to generate a reliable FP assay window (Figure S16a), whereas LC‐YD03 retained activity against YTHDC1(WT), YTHDC1(N363A), and YTHDC1(S378A), with IC_50_ values of 290.7 ± 27.01 nm, 396.9 ± 46.29 nm, and 476.3 ± 27.13 nm, respectively (Figure S16b–d). In AlphaScreen assays, the tested mutants also failed to produce reliable assay windows, likely due to impaired interaction with the m^6^A RNA probe (Figure S16e). In addition, YTHDC1(N367A) could not be successfully purified, suggesting a role in protein stability or folding. These mutagenesis results support the binding mode revealed by the crystal structure and identify N364 as an important residue for LC‐YD03 binding and inhibition.

### 2.8 Cellular Activity Evaluation of LC‐YD17, an Optimized YTHDC1 Inhibitor Derived from LC‐YD03

We next assessed the cellular inhibitory activity of LC‐YD03 toward MOLM‐13 and THP‐1 cells; unfortunately, this compound showed limited activity in both cell types (Figure S17a,b). To investigate whether this was associated with insufficient cellular exposure, we quantified intracellular LC‐YD03 levels in THP‐1 cells by LC‐MS/MS. After treatment with 30 µm LC‐YD03 for 2 h, the ratio of compound levels in the cell lysate to those in the dosing medium was only 0.08%, markedly lower than that of the reported positive control compound **25**, 2.29% (Figure S17c). This result suggests that the weak cellular activity of LC‐YD03 may be largely associated with its poor intracellular exposure, likely due to its relatively high polarity, low lipophilicity, and potential efflux liability.

**FIGURE 6:**
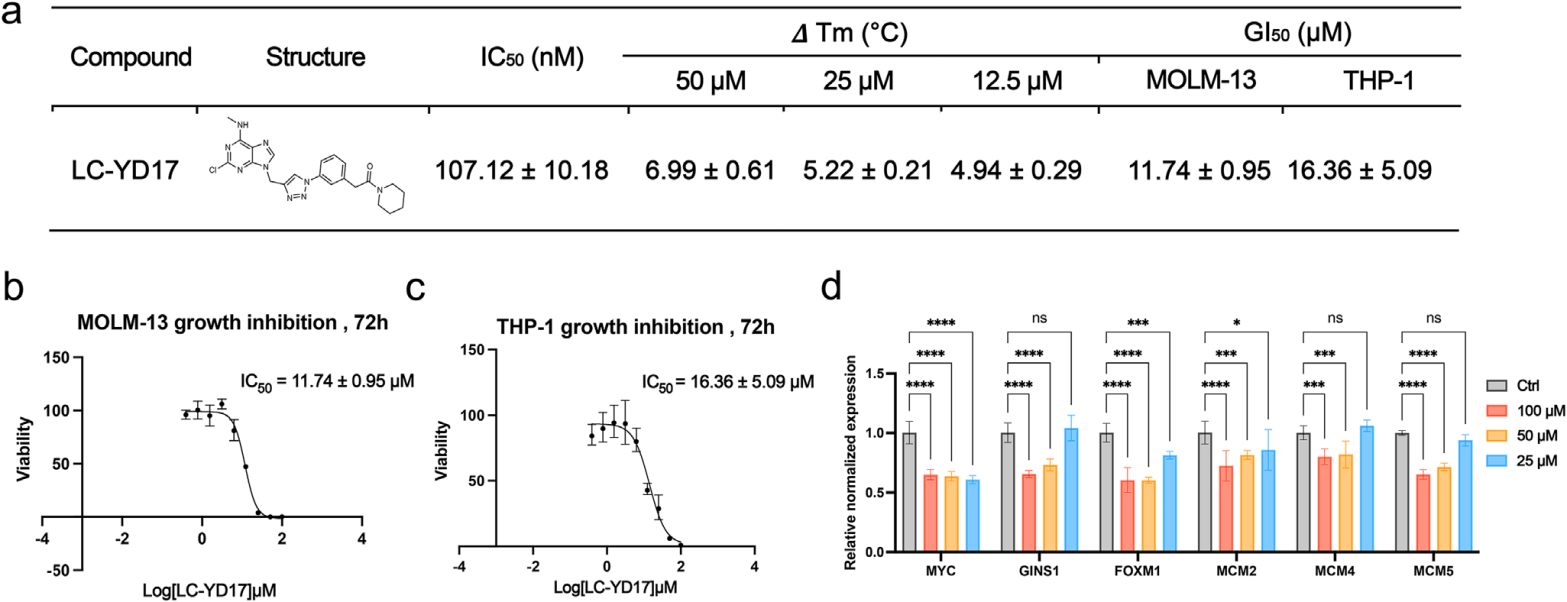
Characterization of LC‐YD17. (a) Chemical structures, AlphaScreen IC_50_ values, TSA ΔTm values, and CTG GI_50_ values of LC‐YD17 (n = 3). (b,c), Growth inhibition curves of LC‐YD17 in MOLM‐13 and THP‐1 cells after 72 h treatment, measured by CTG assay(n = 3). (d) RT‐qPCR analysis of MYC, GINS1, FOXM1, MCM2, MCM4, and MCM5 mRNA levels after LC‐YD17 treatment. Data are shown as mean ± SD from three independent experiments. *P* values were calculated compared with the control group; ns, not significant; ^*^ *P* < 0.05; ^***^ *P* < 0.001; ^****^ *P* < 0.0001.

Prompted by these observations, we designed and synthesized a series of focused analogs of LC‐YD03 to improve its cellular exposure and further elucidate its structure–activity relationship (SAR). As shown in Figure S17d, initial modifications targeted the triazole ring (LC‐YD07‐10), followed by variations of the carboxylic acid moiety (LC‐YD11‐19). The biochemical (AlphaScreen and TSA) and cellular (CellTiter‐Glo, CTG) assay results are summarized in Figure S17e and partially presented in Figure S18. Among them, LC‐YD17 showed improved and relatively consistent growth inhibitory activity in MOLM‐13 and THP‐1 cells, with IC_50_ values of 11.74 and 16.36 µm, respectively (Figure 6a–c). TSA and AlphaScreen assays further confirmed that LC‐YD17 retained in vitro YTHDC1 inhibitory activity, with an AlphaScreen IC_50_ of 107.12 ± 10.18 nm (Figure 6a). In addition, LC‐YD17 maintained favorable selectivity within the YTH family, showing preferential inhibition of YTHDC1 over other tested YTH family proteins in both FP and AlphaScreen assays (Figure S19a,b). LC‐YD17 also showed no obvious inhibition of the METTL3/METTL14 complex within the tested concentration range (Figure S19c).

To further assess whether LC‐YD17 could affect YTHDC1‐related molecular events in cells, we examined the expression of six previously reported YTHDC1‐associated downstream transcripts in myeloid leukemia, including MYC, FOXM1, GINS1, MCM2, MCM4, and MCM5, by reverse transcription quantitative polymerase chain reaction (RT‐qPCR) [32, 33]. After 48 h of treatment, LC‐YD17 reduced the mRNA levels of these YTHDC1‐associated downstream transcripts, particularly at 100 and 50 µm (Figure 6d). These results indicate that LC‐YD17, as an optimized derivative of LC‐YD03, retains YTHDC1 inhibitory activity, exhibits improved cellular activity and favorable family selectivity, and can modulate YTHDC1‐associated transcript expression in cells.

## 3 Conclusions

**FIGURE 7:**
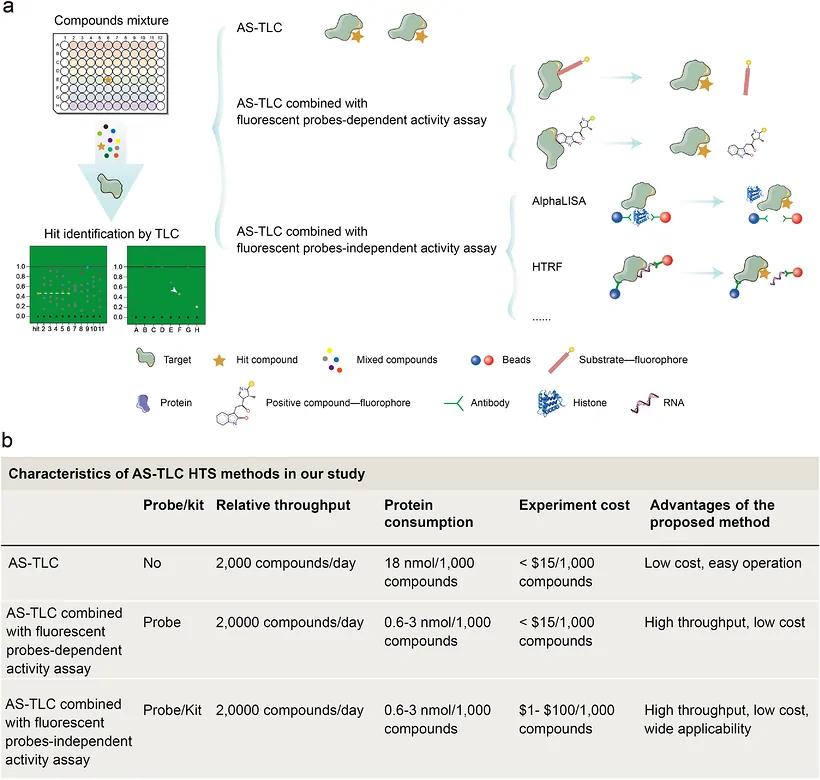
(a) Flowchart and potential application of the three AS‐TLC‐based screening methods. (b) Characteristics of AS‐TLC HTS methods in our study.

Mixed compound screening strategies, such as AS‐MS, DEL and NMR, are increasingly used in lead compound development. However, AS‐MS relies on expensive mass spectrometry to determine the hit compound, DEL requires next‐generation sequencing and DNA tagging–tags may interfere with compound synthesis or bioactivity, NMR‐based approaches are constrained by challenges in deconvoluting complex mixtures and the high technical demands associated with instrumentation and operation, restricting widespread adoption. Here we developed an AS‐TLC HTS method, a low‐cost, accessible mixed‐compound screening strategy that enhanced screening throughput and reduced cost (Table S6). Subsequent validation showed its adaptability to multiple scenarios (Figure 7a,b): (1) AS‐TLC only relies on protein binding with hits and does not require probes or kits, which makes it available to many targets. (2) In AS‐TLC combined with a fluorescent probe‐dependent activity assay, pre‐screening with activity assays significantly reduced protein dosage. And this integrated method helps lower the false positive rate. (3) In AS‐TLC combined with a fluorescent probe‐independent activity assay, false‐positive rates are reduced while screening costs for assays relying on expensive commercial kits are cut by at least 1/100. We also conducted parallel screening experiments to compare the costs and screening outcomes of AS‐MS and AS‐TLC combined with activity assay. The screening strategy we developed exhibited advantages of shorter screening duration, lower protein consumption (Figures S20 and S21, Table S7, Figures S22 and S23, Table S8), and higher sensitivity (Figures S24 and S25, Table S9). However, AS‐TLC also has limitations: (1) Detergents in reaction solutions impair SEC‐mediated separation of unbound compounds, precluding its use for membrane protein targets (Figure S26a,b); (2) Covalent compounds cannot be effectively separated, limiting applicability (Figure S26c,d); (3) Compounds with weak UV absorption are harder to detect, though this has minimal impact on identifying high‐affinity hits.

While AS‐TLC identified hits, hit optimization remains a bottleneck. Conventional medicinal chemistry typically focuses on sequential, stepwise construction of individual compounds, which is time‐consuming and cost‐prohibitive. In contrast, CuAAC‐based combinatorial library technology employs parallel synthesis to rapidly yield extensive derivatives simultaneously. Additionally, this biocompatible synthetic method enables rapid in situ evaluation of large compound sets—eliminating the need for compound purification.

Building on these, this work not only develops AS‐TLC as a straightforward, accessible method for hit identification from compound mixtures—while further validating the feasibility and efficiency of integrating with functional activity assays—but also advances a “hit discovery‐to‐optimization” pipeline tailored for challenging targets like YTHDC1. Specifically, after identifying initial YTHDC1 hits via the AS‐TLC‐activity‐based screening system, we leveraged CuAAC‐based combinatorial library technology to rapidly synthesize a large‐scale derivative library and ultimately yielded potent YTHDC1 inhibitors with significantly enhanced activity. These chemical and biological steps can potentially be consolidated in 384‐well plates, enabling full automation.

This strategy reduces costs and improves efficiency in early drug discovery by prioritizing rapid access to viable leads over time‐consuming, stepwise meticulous optimization. In doing so, it provides a broadly applicable framework for accelerating the discovery of inhibitors against intractable biological targets, not just for YTHDC1 but for a wide range of therapeutic targets in future research.

## 4 Methods

### 4.1 Chemical Synthesis and General Methods

Detailed procedures for the synthesis and chemical characterization of all compounds are given in the Supporting Information.

### 4.2 Expression and Purification of CECR2 Protein

The GST‐CECR2 (424‐538) construct was cloned into the pGEX‐6p‐1 vector and expressed in *Escherichia coli* BL21(DE3) cells. The cells were cultured in LB medium at 37°C until the optical density at 600 nm (OD600) reached 0.6. Protein expression was induced by adding 0.4 mm IPTG, followed by incubation for an additional 16 hours. The harvested cell pellet was resuspended in buffer A (20 mm HEPES, pH 7.4, 150 mm NaCl, 1 mm DTT, 5% glycerol) and disrupted via sonication. After centrifugation, the supernatant was loaded onto a GSTrap FF column (GE Healthcare) pre‐equilibrated with buffer A. The column was washed with buffer A until the absorbance at 280 nm (A280) returned to baseline, and the target protein was eluted using buffer A supplemented with 20 mm GSH. For further refinement, the eluted protein underwent size‐exclusion chromatography using a Superdex 75 Increase 10/300 GL column (GE Healthcare) equilibrated with SEC buffer (20 mm HEPES, pH 7.4, 150 mm NaCl). The purified protein was then concentrated, divided into aliquots, flash‐frozen in liquid nitrogen, and stored at −80°C for subsequent applications.

### 4.3 Expression and Purification for USP7 Protein

The His‐USP7 (208–560) construct was inserted into the pET‐28a vector and expressed in *Escherichia coli* BL21(DE3) cells. The bacterial cultures were grown in LB medium at 37°C until the optical density at 600 nm (OD600) reached 0.6. Subsequently, the temperature was lowered to 16°C, and protein expression was induced with 0.4 mm IPTG, followed by incubation for an additional 14 h. The collected cell pellet was resuspended in Buffer A (50 mm HEPES, pH 7.4, 300 mm NaCl, 5 mm β‐mercaptoethanol, 15 mm imidazole) and lysed via sonication. The supernatant obtained after centrifugation was loaded onto a cobalt affinity column pre‐equilibrated with Buffer A. The column was washed with Buffer A until the absorbance at 280 nm (A280) returned to baseline, and the target protein was eluted using Buffer B (50 mm HEPES, pH 7.4, 300 mm NaCl, 5 mm β‐mercaptoethanol, 300 mm imidazole). The eluted protein was desalted into a storage buffer (50 mm HEPES, pH 7.4, 150 mm NaCl, 1 mm DTT) using a desalting column. The purified protein was subsequently concentrated, divided into aliquots, flash‐frozen in liquid nitrogen, and stored at −80°C.

### 4.4 Expression and Purification for CDK2 Protein

The full‐length GST‐CDK2 construct was cloned into the pGEX‐6p‐1 vector and expressed in *Escherichia coli* BL21(DE3) cells. The cells were cultured in LB medium at 37°C until the optical density at 600 nm (OD600) reached 0.6. Protein expression was induced with 0.4 mm IPTG, and the culture was incubated at 18°C for 16 h. The cell pellet was resuspended in buffer A (20 mm HEPES, pH 7.4, 150 mm NaCl, 10 mm β‐mercaptoethanol) and lysed via sonication. After centrifugation, the supernatant was loaded onto a GSTrapTM FF column (GE Healthcare) pre‐equilibrated with buffer A. The column was washed with buffer A until the absorbance at 280 nm (A280) returned to baseline, and the target protein was eluted using buffer A supplemented with 20 mm GSH. Subsequent to buffer exchange, HRV 3C protease was added to the concentrated purified protein to cleave the N‐terminal tags. This mixture was incubated at 4°C for 16 h. The tag‐free CDK2 protein underwent further purification using a Superdex 200 Increase 10/300 GL column (GE Healthcare). The final eluate was concentrated, flash‐frozen in liquid nitrogen, and stored at −80°C.

### 4.5 Expression and Purification for YTHDC1, YTHDF1, YTHDF2, YTHDF3

Proteins were expressed and purified as described previously [34]. The YTH domains of human YTH family proteins (YTHDC1: residues 344–509; YTHDF1: residues 364–559; YTHDF2: residues 385–579; YTHDF3: residues 391–585) were subcloned into the pET28a vector. The constructed plasmid was transformed into *E. coli* BL21(DE3) cells. The cells were grown at 37°C with shaking at 220 rpm until the optical density (OD) reached 0.6–0.8. The temperature was then reduced to 16°C and protein expression was induced by adding 0.4 mm IPTG for 14–16 h. The bacterial cultures were harvested by centrifugation at 4°C. The cell pellets were resuspended in Ni‐A buffer (20 mm Tris‐HCl pH 7.4, 150 mm NaCl, 0.7% β‐mercaptoethanol) and lysed by sonication. The lysate was centrifuged at 16 000 rpm for 60 min at 4°C. The supernatant was loaded onto a GE HisTrap HP affinity chromatography column pre‐equilibrated with Ni‐A buffer, and purification was performed using an ÄKTA purification system. Non‐specifically bound proteins were washed off with Ni‐A buffer at a flow rate of 3 mL/min. The target protein was eluted with the appropriate Ni‐B buffer (20 mm Tris‐HCl pH 7.4, 150 mm NaCl, 1 m imidazole, 0.7% β‐mercaptoethanol). The eluted protein was then exchanged into Ni‐A buffer using a GE HiTrap desalting column. The desalted protein was incubated with Tobacco etch virus (TEV) protease overnight at 4°C to remove the N‐terminal tag. Subsequently, the mixture was diluted to an appropriate concentration with SP‐A buffer (20 mm Tris‐HCl pH 7.4, 20 mm NaCl, 0.7% β‐mercaptoethanol) and loaded onto an SP column. The target protein was eluted with the appropriate SP‐B buffer (20 mm Tris‐HCl pH 7.4, 2 m NaCl, 0.7% β‐mercaptoethanol). The recombinant protein was further purified by size‐exclusion chromatography on a Superdex 200 Increase 10/300 GL column (GE Healthcare). Finally, the purified protein was concentrated to an appropriate concentration and stored at −80°C. The same expression and purification procedure was used for mutant YTHDC1 proteins.

### 4.6 Expression and Purification for YTHDC2

Proteins were expressed and purified as described previously [35]. Human YTHDC2, residues 1287–1423, was cloned into the pGEX‐6P1 vector and expressed in *Escherichia coli* BL21(DE3) cells cultured in LB medium supplemented with ampicillin. Protein expression was induced with 0.2 mm IPTG at 16°C for 15 h. The cell pellet was resuspended in buffer A (20 mm HEPES, pH 7.4, 150 mm NaCl, 10 mm β‐mercaptoethanol) and lysed via sonication. After centrifugation, the supernatant was loaded onto a GSTrapTM FF column (GE Healthcare) pre‐equilibrated with buffer A. The column was washed with buffer A until the absorbance at 280 nm (A280) returned to baseline, and the target protein was eluted using buffer A supplemented with 20 mm GSH. The GST tag was cleaved using PreScission Protease, and the recombinant protein was further purified by size‐exclusion chromatography on a Superdex 200 Increase 10/300 GL column (GE Healthcare). Purified proteins were stored at −80°C in buffer containing 20 mm Tris‐HCl, pH 7.4, and 150 mm NaCl.

### 4.7 Expression and Purification of METTL3–METTL14

Full‐length METTL3, residues 1–580, and full‐length METTL14, residues 1–456, were co‐transformed into *E. coli* BL21(DE3) competent cells at a plasmid ratio of 1:2. Transformants were selected on LB agar plates containing ampicillin and kanamycin. A single colony was inoculated into LB medium with the corresponding antibiotics and cultured overnight at 37°C. The overnight culture was then transferred into 1 L LB medium and grown at 37°C until the OD_600_ reached approximately 0.8. Protein expression was induced with 0.5 mm IPTG at 16°C for 14–16 h. Cells were harvested by centrifugation at 8000 rpm for 10 min at 4°C and resuspended in lysis buffer (25 mm HEPES, 1 m NaCl, pH 7.4). After sonication on ice, the lysate was clarified by centrifugation at 18 000 rpm for 90 min at 4°C. The supernatant was loaded onto a 5 mL HisTrap FF Ni‐affinity column pre‐equilibrated with buffer A (25 mm HEPES, 250 mm NaCl, 20 mm imidazole, pH 7.4). Bound proteins were eluted with an increasing gradient of buffer B (25 mm HEPES, 100 mm NaCl, 250 mm imidazole, pH 7.4). Fractions containing METTL3–METTL14 were identified by SDS‐PAGE and Coomassie Brilliant Blue staining, pooled, and concentrated. The sample was further purified by size‐exclusion chromatography on a Superdex 200 Increase 10/300 GL column using buffer 2 (25 mm HEPES, 100 mm NaCl, pH 7.4). Purified protein fractions were analyzed by SDS‐PAGE, concentrated to 32 µm, flash‐frozen in liquid nitrogen, and stored at −80°C until use.

### 4.8 AS‐TLC Screening Method

Here we provide a general set of AS‐TLC experimental conditions, which is also the condition used for screening hit compounds targeting YTHDC1 in the main text. For other specific applications, protein and compound concentrations can be adjusted according to the potency of the expected hit compounds. The freeze‐dried mixed samples of 160 compounds were reconstituted in 5 µL of DMSO and diluted with 45 µL of PBS buffer to achieve a compound concentration of 100 µm. The protein was diluted in PBS to a concentration of 100 µm. Equal volumes of compound solution (50 µL) and protein solution (50 µL) were combined. After incubation, the mixture was centrifuged to remove any potential compound precipitates. The supernatant was desalted using Spin Desalting Columns. To denature the protein, 300 µL of acetonitrile was added to the eluate. Protein precipitates were removed by centrifugation, and the resulting supernatant was freeze‐dried. The freeze‐dried samples, along with the 10 groups of compounds (16 compounds per group) from the initial mixing step, were dissolved in 5 µL of ethyl acetate and spotted onto the same silica gel plate. Once the spots dried, the compounds were sequentially developed using five polarity gradient developing agents until the R*_f_* values of the “hit compounds” ranged between 0.2 and 0.8. The group containing the “hit compounds” was identified. The 16 compound standards from the identified group were dissolved in 5 µL of ethyl acetate and spotted on the same silica gel plate. The developing agent from the final step of the previous procedure was used to pinpoint the specific “hit compound.” All compounds were freeze‐dried before being spotted onto the silica gel plate.

### 4.9 Substrate‐Fluorophore Combined with AS‐TLC

After freeze‐drying mixed compounds (160 compounds per group), each group was redissolved in 0.5 µL of DMSO. Subsequently, 9.5 µL of 500 nm USP7 protein was added to each group, resulting in a final compound concentration of 2.5 µm, and incubated at room temperature for 30 min. Then, 10 µL of 1 µm ubiquitin‐rhodamine 110 was introduced, and the enzymatic reactions were conducted at 37°C for 1 h. Upon completion, the reaction mixtures were spotted onto silica gel plates using multichannel pipettes, and the solvents were evaporated with a heat gun. The samples were developed using ethyl acetate: methanol (4:1, v/v). The fluorescence intensity of rhodamine 110 in each group was observed under 365 nm UV light. For mixed compounds exhibiting USP7 inhibition activity, AS‐TLC methods were applied to identify lead compounds.

### 4.10 USP7 Activity Assay

To determine compound IC_50_ values, compounds were diluted in a threefold, 9‐point dilution series starting from 100 µm. Equal volumes of compound and protein were mixed and incubated at room temperature for 30 min. Subsequently, 5 µL of a 1 µm ubiquitin‐rhodamine 110 solution was added to the protein‐compound mixture and incubated at 37°C for 60 min. Rhodamine 110 emission was detected at 520 nm upon excitation at 485 nm using Multimode Plate Reader (EnVision 2015). The reactions were performed in a black 384‐well plate with a total volume of 10 µL. The reaction buffer comprised 50 mm Tris‐HCl, pH 7.4, 100 mm NaCl, 0.05% CHAPS, and 1 mm EDTA, pH 8.0.

### 4.11 Positive Compound‐Fluorophore Combined with AS‐TLC

After freeze‐drying the mixed compounds (160 compounds per group), each group was reconstituted in 1 µL of DMSO. Subsequently, 49 µL of a 2 µm CDK2 protein and 1 µm CPJ‐4‐1 mixed solution was added to each group and incubated at room temperature for 30 min. The reaction mixtures were desalted, and 20 µL of the eluate from each group was spotted. Fluorescence was examined under 365 nm UV light. For groups displaying CDK2 inhibition activity, AS‐TLC methods were employed to identify hit compounds.

### 4.12 Homogeneous Time‐Resolved Fluorescence (HTRF) Combined with AS‐TLC

After freeze‐drying the mixed compounds, they were reconstituted in 0.8 µL of DMSO. The diluted YTHDC1 protein and compounds were incubated at 37°C for 30 min. And then, biotin‐mRNA was added, and incubated for 10 min. The final concentrations of the compounds, YTHDC1 protein, and biotin‐mRNA were 10, 200, and 40 nm, respectively, with a total reaction volume of 15 µL. The HTRF reaction solution consisted of 20 mm HEPES (pH 7.4), 150 mm NaCl, 1 mg/mL BSA, and 0.01% TritonX‐100. Subsequently, 5 µL mixture of acceptor beads and donor beads was added, followed by incubation at room temperature for 10 min. The signal was measured in HTRF mode using the Multimode Plate Reader (EnVision 2015). For groups of mixed compounds demonstrating YTHDC1 inhibitory activity, AS‐TLC was utilized to identify hit compounds.

### 4.13 Thermal Shift Assay (TSA)

TSA buffer contained 20 mm HEPES (pH 7.4) and 150 mm NaCl. Protein solution (final concentration 10 µm) was mixed with varying concentrations of test compounds. SYPRO Orange dye was added in the dark to a final concentration of 10×. The microplate was then transferred to a QuantStudio 6 Flex Real‐Time PCR System (Applied Biosystems) for reading. The temperature was gradually increased from 25°C to 95°C over 25 min, and fluorescence signals were recorded as raw data at each time point. Protein TmD values were analyzed using Protein Thermal Shift Software v1.3.

### 4.14 Amplified Luminescent Proximity Homogeneous Assay Screen (AlphaScreen)

AlphaScreen assay buffer contained 20 mm HEPES (pH 7.4), 150 mm NaCl, 1 mg/mL bovine serum albumin (BSA), and 0.01% (v/v) Triton X‐100. Test compounds were diluted with His‐ YTH family proteins (final concentration 200 nm). 20 nm (final concentration) synthetic single‐stranded oligoribonucleotide (YTHDC1: 5′‐biotin‐GAACCGGm^6^ACUGUCUUA‐3′; YTHDF1/2/3: 5′‐biotin‐UUCUUCUGUGG(m^6^A)CUGUG‐3′, GenScript) was then added, followed by incubation on ice for 15 min. Streptavidin‐coated donor beads and anti‐His_6_ acceptor beads were diluted 100‐fold in assay buffer under light‐protected conditions and transferred to corresponding wells, followed by incubation at 4°C for 1 h. Quantification of AlphaScreen signals was performed on an EnVision multilabel reader using 570‐nm excitation. Data were processed in Excel and analyzed using GraphPad Prism 8.3.0.

### 4.15 YTH Family Proteins Fluorescence Polarization (FP)

FP assay buffer contained 20 mM HEPES (pH 7.4), 50 mm NaCl, 0.01% (v/v) Tween 20, and 5% (v/v) glycerol. Test compounds were diluted and transferred to black 384‐well plates (Corning, #3575). After initial gain calibration using blank buffer, 250 nm YTH family proteins were individually added and incubated at room temperature for 30 min. Subsequently, 25 nm fluorescent probes (YTHDC1: FP‐YD; YTHDF1/2/3: 5′‐FAM‐ UUCUUCUGUGG(m^6^A)CUGUG‐3′, GenScript; YTHDC2: 5′‐FAM‐UUCUUCUGUGG(m^6^A)CUGUG‐3′, GenScript) were introduced under light‐protected conditions, followed by additional incubation at 4°C for 30 min. FP was measured on an EnVision plate reader (λex/λem = 480/535 nm). Data were processed in Excel and analyzed using GraphPad Prism 8.3.0.

### 4.16 METTL3–METTL14 Fluorescence Polarization

The METTL3–METTL14 fluorescence polarization assay was performed in two steps. In the reaction step, the RNA probe(5′‐FAM‐GAACCGGACUGUCUUA‐3′), METTL3/METTL14 complex, and SAM were diluted in reaction buffer (20 mm HEPES, pH 7.4, 1 mm DTT, and 0.4 U/µL RNase inhibitor) and added sequentially to black 384‐well plates (Corning, #3575). The reaction volume was 20 µL, and the mixture was incubated at room temperature for 60 min. In the termination step, 20 µL YTHDF1 protein diluted in termination buffer (20 mm HEPES, pH 7.4, and 500 mm NaCl) was added to each well, giving a final volume of 40 µL. Plates were mixed at 1000 rpm for 2 min using a MixMate shaker (Eppendorf). Fluorescence polarization was measured on an EnVision multimode plate reader with excitation at 480 nm and emission at 535 nm. Data were analyzed and plotted using GraphPad Prism 8.3.0.

### 4.17 Biolayer Interferometry (BLI)

The BLI assay was conducted to determine the binding affinity between YTHDC1 protein and compounds using an OCTET R8 instrument (SARTOTIUS) at 25°C. YTHDC1 protein was immobilized on SSA biosensors to achieve signal up to 12 nm, a row of biosensors without immobilized protein served as the control reference. Compounds were dissolved in PBS buffer and serially diluted to different concentrations. Biosensor was soaked in compound solution, allowing binding to the immobilized protein for 450 seconds, followed by dissociation for 600 s. The dissociation constant (K_d_) of compounds for YTHDC1 protein was calculated using Octet Analysis Studio 13.0.

### 4.18 Crystallization and Structure Determination of the YTH Domain of YTHDC1 Protein in Complex with Compounds Fragment YD, LC‐YD01, and LC‐YD03

Crystals were grown at 18°C using the hanging‐drop vapor diffusion method. 1 µL drop containing 10 mg/mL YTHDC1 protein solution was mixed with 1 µL of reservoir solution (0.1 m Bis‐Tris pH 7.5, 0.2 m ammonium sulfate, 20% (w/v) PEG 3350) and equilibrated against 600 µL of reservoir solution. Crystals typically formed within approximately one week. To obtain protein‐fragment complex crystals, native crystals were transferred and soaked overnight at 16°C in a 2 µL drop containing 2 mm compound dissolved in 0.1 m Bis‐Tris pH 7.5, 0.2 m ammonium sulfate, and 30% (w/v) PEG 3350. Soaked crystals were then flash‐cooled in liquid nitrogen for X‐ray diffraction data collection. All diffraction data were collected at beamline BL18U1 of the National Center for Protein Science Shanghai (NCPSS) at the Shanghai Synchrotron Radiation Facility (SSRF). Data were processed using the HKL3000 program suite and the XDS software package. Structures were solved by molecular replacement using Phaser from the Phenix package. Model building and refinement were performed using COOT^40^ and phenix. Refine, respectively. All structural figures were prepared using PyMOL.

### 4.19 Cell Culture

THP‐1 and MOLM‐13 cell lines were obtained from the American Type Culture Collection (ATCC). Cells were cultured in RPMI 1640 medium (ShareBio) containing 10% FBS (Biological Industries) and 1% penicillin–streptomycin (Gibco) in 5% CO2 at 37°C in a humidified incubator.

### 4.20 Cytotoxicity

Cells were plated in white, clear‐bottom 96‐well plates at 3 × 10^3^ cells per well in 100 µL of complete RPMI medium. An equal volume of medium containing serially diluted test compounds in DMSO was then added to achieve final compound concentrations of 0.4–100 µm. Cells treated with 0.5% DMSO, v/v served as the vehicle control. Plates were incubated for 72 h at 37°C in a humidified incubator with 5% CO_2_. Cell viability was determined using a CellTiter‐Glo luminescent cell viability assay (Vazyme) based on the detection of ATP according to the manufacturer's instructions. 20 µL of the reagent was added to each well and incubated for 15 min at room temperature on an orbital shaker. After incubation, the resulting reaction mixture was transferred to a white opaque polystyrene 96‐well assay plate, with 70 µL transferred into each well. The luminescence signal of each sample well was then immediately measured using the Lumi mode of an EnVision multimode plate reader. All procedures were performed at room temperature and protected from light. Finally, the luminescence data were exported and analyzed using GraphPad Prism 8.3.0. Cell viability inhibition curves were generated by curve fitting.

### 4.21 Kinases Selectivity

For kinase inhibition testing, a customized 41‐kinase profiling panel was performed by Hefei Zhongke PreceDo Biopharmaceutical Technology Co., Ltd. using the ADP‐Glo assay. LC‐YD03 was tested at a single concentration of 1000 nm, in two replicates, against a panel of 41 kinases. Briefly, kinase and compound were pre‐incubated at room temperature for 5 min, followed by addition of the substrate/ATP mixture and incubation for 60 min. ADP‐Glo Reagent and Kinase Detection Reagent were then added sequentially, and luminescence was recorded using a microplate reader. The inhibition rate was calculated using DMSO as the 0% inhibition control and the corresponding positive inhibitor as the 100% inhibition control.

### 4.22 THP‐1 Cellular Uptake Assay

THP‐1 cells were cultured in RPMI 1640 medium supplemented with 10% fetal bovine serum and 1% penicillin‐streptomycin at 37°C in a humidified incubator containing 5% CO_2_. Cells were seeded into 6‐well plates at a density of 1 × 10^6^ cells per well in 2 mL of culture medium. Stock solutions of LC‐YD03 and compound **25** at 30 mm were diluted in RPMI 1640 medium to a final concentration of 30 µm and incubated with the cells at 37°C for 4 h. After incubation, the cells were collected and centrifuged at 1000 rpm for 5 min. The supernatant was collected as the dosing medium sample. The cell pellets were washed three times with ice‐cold PBS. Subsequently, 500 µL of ACN/H_2_O, 4:1, v/v, was added to each well, and the cells were lysed by sonication. The cell lysates were collected and centrifuged at 12 000 rpm for 10 min. The resulting supernatants were subjected to LC‐MS/MS analysis.

### 4.23 RT‐qPCR

MOLM‐13 cells were treated with DMSO, LC‐YD17 at indicated concentrations for 48 h. Total RNA was extracted using TRIzol reagent (Vazyme). Briefly, cells were lysed with 500 µL TRIzol reagent (Vazyme) for 5 min, followed by extraction with 200 µL chloroform. After centrifugation at 12 000 rpm for 10 min at 4°C, the aqueous phase was mixed with 0.5 volume of absolute ethanol and loaded onto an RNA purification column (BBI). The column was washed twice with 500 µL 1× RPE buffer, and RNA was eluted with 30 µL DEPC‐treated water. RNA concentration was measured using a NanoDrop spectrophotometer. cDNA was synthesized using reverse transcription reagents from Vazyme and diluted to 200 ng/µL. qPCR was performed using the SYBR Green method with Vazyme qPCR reagents. Gene expression was calculated using the comparative ΔΔCt method with the GAPDH for normalization. All primers of the selected genes were listed as follows:

GAPDH, forward, 5′‐ GAAATCCCATCACCATCTTCCAGG ‐3′;

GAPDH, reverse, 5′‐GAGCCCCAGCCTTCTCCATG‐3′;

MYC, forward, 5′‐TTCGGGTAGTGGAAAACCAG‐3′;

MYC, reverse, 5′‐CAGCAGCTCGAATTTCTTCC‐3′;

GINS1, forward, 5′‐GGTCACTGGGAGGAGATGAA‐3′;

GINS1, reverse, 5′‐GCTCACATTTCCATCGAGGT‐3′;

FOXM1, forward, 5′‐ACCCAAACCAGCTATGATGC‐3′;

FOXM, reverse, 5′‐GAAGCCACTGGATGTTGGAT‐3′;

MCM2, forward, 5′‐CCGTGACCTTCCACCATTTGA‐3′;

MCM2, reverse, 5′‐GGTAGTCCCTTTCCATGCCAT‐3′;

MCM4, forward, 5′‐GACGTAGAGGCGAGGATTCC‐3′;

MCM4, reverse, 5′‐GCTGGGAGTGCCGTATGTC‐3′;

MCM5, forward, 5′‐ATGTCGGGATTCGACGATCCT‐3′;

MCM5, reverse, 5′‐CCAGGTTGTAATGCCGCTTG‐3′.

### 4.24 AS‐MS (Affinity Selection‐Mass Spectrometry)

80 µL of a mixture containing 160 compounds dissolved in DMSO was placed in a freeze dryer to remove the solvent. The lyophilized compounds were redissolved in 8 µL of DMSO, followed by dilution with PBS, and then mixed with protein solution. The working concentration of the protein was 50 µm, and the concentration of each individual compound was 50 µm, with a total reaction volume of 100 µL. After incubation at room temperature for 30 min, unbound small‐molecule compounds were removed using a desalting column. 300 µL of acetonitrile was added to 100 µL of the effluent, and the mixture was centrifuged at 15 000 rpm for 10 min. The supernatant was collected and lyophilized to remove the solvent. The sample was redissolved in 100 µL of methanol, filtered through a 0.22 µm membrane filter, and then subjected to mass spectrometry analysis.

The mass spectrometry detection method was: Mass spectra were acquired on a Waters Acquity UPLC H‐Class Plus/ACQUITY QDA mass spectrometer equipped with an electrospray ionization (ESI) source operating in positive ion mode. Separation was performed on an ACQUITY UPLC BEH C18 column (2.1 × 50 mm, 1.7 µm). The chromatographic conditions were as follows: flow rate 0.3 mL/min; mobile phase A, 0.1% formic acid in water; mobile phase B, acetonitrile. The gradient program was: 5% B maintained initially, increased linearly to 95% B over 4 min, held at 95% B for 1 min, decreased linearly to 5% B over 0.1 min, and maintained at 5% B for 0.9 min. Mass spectra were acquired in full scan mode over the range 50.00–1250.00 Da, using centroid mode with a cone voltage of 15 V.

## Author Contributions

H.X., C.L., and S.C. initiated and designed the project, M.W. performed the development of AS‐TLC assay, M.W., H.X., and Y.X. performed protein expression and validation of AS‐TLC assay, M.W. and S.F. performed the YTHDC1 screening assay, S.F. performed the screening of YTHDC1 derivatives and complexes structural determination through X‐ray crystallography, H.X. L.J. designed the YTHDC1 compounds, J.G., Y.W., J.R., and X.L. synthesized and characterized the YTHDC1 compounds, P.C. and H.L. helped synthesize the probe of CDK2, G.S. provided CDK2 protein, M.W., S.F., H.X.,. C.L. and S.C. wrote and revised the manuscript. All authors discussed the results and commented on the manuscript.

## Conflicts of Interest

The authors declare no conflict of interest.

## Supporting information




**Supporting File 1**: advs77386‐sup‐0001‐SuppMat.docx.

**Supporting File 2**: advs77386‐sup‐0002‐SuppMat.docx.

## Data Availability

The data that support the findings of this study are available on request from the corresponding author. The data are not publicly available due to privacy or ethical restrictions.
