# Supplementary material for: AS‐TLC‐Based High‐Throughput Screening Integrated with CuAAC Optimization Strategy Facilitates the Rapid Discovery of YTHDC1 Inhibitor
Source: Adv Sci (Weinh). 2026 Aug 27;13(63):e77386. doi: 10.1002/advs.77386 (PMC13521195; doi:10.1002/advs.77386)
Supplement: Supplementary file 2 [file ADVS-13-e77386-s002.docx]

**Supplementary Figures and Tables**

**AS-TLC-Based High-Throughput Screening Integrated with CuAAC Optimization Strategy Facilitates the Rapid Discovery of YTHDC1 Inhibitor**

Mingchen Wang ^a, b, c, d, +^, Shuiping Fu ^d, e, +^, Ye Xu ^a, d, +^, Jia Gao ^f^, Jiwei Ren ^f^, Xu Yang ^c^, Hesong Xu ^a^, Ge Sun ^d^, Yanjie Li ^a, d^, Yanlin Wang ^f^, Lu Jin ^f^, Peijuan Chen ^g^, Ying Xu ^d^, Jianhao Li ^a, c^, Hua Lin ^g^, Chujiao Hu ^b^, Kaixian Chen ^d^, Huan Xiong ^f, *^, Cheng Luo ^a, b, d, e, h, *^, Shijie Chen ^c, d, *^

*^a^ School of Pharmaceutical Science and Technology, Hangzhou Institute for Advanced Study, University of Chinese Academy of Sciences, Hangzhou, 310024, China.*

*^b^ Precision Medicine Research Institute of Guizhou, and Guizhou Provincial Key Laboratory of Digestive System Diseases, The Affiliated Hospital of Guizhou Medical University, Guizhou Medical University, Guiyang, 550014, China.*

*^c^ Innovation Center for AI and Drug Discovery, School of Pharmacy, East China Normal University, Shanghai 200062, China.*

*^d^ State Key Laboratory of Drug Research, Shanghai Institute of Materia Medica, Chinese Academy of Sciences, Shanghai, 201203, China.*

*^e^ University of Chinese Academy of Sciences, Beijing 100049, China*

*^f^ Zhongshan Institute for Drug Discovery, Shanghai Institute of Materia Medica, Chinese Academy of Sciences, Zhongshan 528400, China.*

*^g^ Key Laboratory of Microbial Pathogenesis and Interventions of Fujian Province University, the Key Laboratory of Innate Immune Biology of Fujian Province, Biomedical Research Center of South China, College of Life Sciences, Fujian Normal University, Fuzhou 350117, China.*

*^h^ Tianfu Jincheng Laboratory, Chengdu, 610212, China.*

^+^ *These authors contributed equally to this work.*

*^*^ Corresponding author.*

*E-mail addresses:* [*xionghuan@zidd.ac.cn*](mailto:xionghuan@zidd.ac.cn) *(Huan Xiong); cluo@simm.ac.cn (Cheng Luo); sjchen@pharm.ecnu.edu.cn (Shijie Chen)*

**Supplementary Fig. 1** Determined the experimental conditions for the AS-TLC HTS method. **a-b** Recovery and identification of the positive compound GNE-6640 under varying mixture scales, with individual compound and USP7 protein concentrations both at 50 μM. **c** A “hit” was defined as the compound selected randomly from every group to simulate the hit compound in the real screening situation. They were compared with respective mixtures of 40, 80, 160, compound standards. The white dotted line connects the “hits” against the standards present in the mixed compounds. **d** Schematic diagram of the compound mixing method. Compounds in the same column (rows A-H) of plate 1 and plate 2 were merged into one well in plate 3 to get 10 (columns 2-11) mixtures of 16 compounds. Mixtures of 16 compounds from columns 2-11 in plate 3 were merged into a well in plate 3 to get a mixture of 160 compounds. **e** GNE-6640 initial position in compound library was backtracked in two steps. **f** Hit compound was developed with 160 compounds by gradient polarity developing agent. Developing agent 1: petroleum ether: ethyl acetate (5:1, v/v); Developing agent 2: petroleum ether: ethyl acetate (1:1, v/v); Developing agent 3: 100% ethyl acetate. White dotted lines connect standard compounds in the mixture that match the R*_f_* value and color with hits. **g** The proportion of compounds detectable under ultraviolet light in each group when randomly sampling 40, 80, 160 and 320 specimens from the laboratory's compound library containing 20,000 compounds (n = 3). **h** When the individual compound concentration was fixed at 50 μM, the detectable amount of the positive compound DC-BPi-01 (IC₅₀ = 7.8 μM) by TLC after incubation of mixed compounds with different concentrations of BPTF protein. **i** When the individual compound concentration was fixed at 50 μM, the detectable amount of the positive compound DC-BPi-10 (IC₅₀ = 14.7 μM) by TLC after incubation of mixed compounds with different concentrations of BPTF protein. The red arrows on the ordinate indicate the corresponding Rf values of each positive compound.

**Supplementary Fig. 2** **a** The positive compounds of USP7, GNE-6640 (IC_50_ = 0.4 μM) and USP7-IN-8 (IC_50_ = 1.4 μM). **b** The positive compounds of CDK2, Tagtociclib (IC_50_ = 1.16 nM) and AT7519 (IC_50_ = 47 nM). **c** The positive compounds of CECR2, BAZ2-ICR (IC_50_ = 1.55 μM) and GNE-375 (IC_50_ = 2.4 μM). **d** Positive compounds GNE-6640 and USP7-IN-8 for USP7 were added into the mix of compounds and incubated with USP7. GNE-6640 was identified from column 6, row E of plate 3, and USP7-IN-8 from column 7, row F of plate 8. **e** Positive compounds Tagtociclib and AT7519 were added into the mix of compounds and incubated with CDK2. Tagtociclib was identified from column 5, row B of plate 4, and AT7519 from column 3, row E of plate 8. **f** Positive compounds GNE-375 and BAZ2-ICR were added into the mix of compounds and incubated with CECR2. GNE-375 was identified from column 10, row B of plate 2, and BAZ2-ICR from column 6, row E of plate 10. Yellow arrows indicate hits from compound mixtures. White dotted lines connect standard compounds in the mixture that match the R*_f_* value and color with hits. White arrows indicate the same compound in 16 single compound standards from the previous step identified mixture group.

**Supplementary Fig. 3 a** In single compound reaction systems, Tagtociclib's Z-factor was 0.719. **b** In mixture reaction systems, Tagtociclib's Z-factor was 0.562. **c** n single compound reaction systems, Compound 25's Z-factor was 0.515. **d** In mixed systems, Compound 25's Z-factor was 0.633. **e** In single compound reaction systems, GNE-6640's Z-factor was 0.735. **f** In mixed systems, GNE-6640's Z-factor was 0.517.

**Supplementary Fig. 4** Identification and validation of 164-A02 for CDK2. **a** 164-A02 was identified by AS-TLC. **b** BLI analysis revealed that the dissociation constant (K_d_) between CDK2 and 164-A02 is 1.98 μM. **c** Positive compound 3-95-11 and 164-A02 inhibition for CDK2 (*n* = 3 replicates).

**Supplementary Fig. 5** Schematic diagram of AS-TLC activity assay.

**Supplementary Fig. 6 a** Design of the ubiquitin-rhodamine probe and AS-TLC combination screening assay for USP7. **b** Validation of AS-TLC separating and detecting rhodamine. Rho 110 is rhodamine; NPC indicates the no protein control group, and NCC indicates the no compound control group. “A+” represents the mixture containing GNE-6640, and “A” represents the mixture without inhibitors. **c** Screening and identification of USP7 positive compound GNE-6640. Yellow arrow indicates experimental groups where rhodamine was significantly decreased; white dotted lines connect standard compounds in the mixture that match the R*_f_* value and color with GNE-6640. White arrows indicate GNE-6640.

**Supplementary Fig. 7** Structure of CDK2 fluorescent probe CPJ-4-1.

**Supplementary Fig. 8** **a** Design of CPJ-4-1 and AS-TLC combination screening assay for CDK2. **b** Validating the detection of CPJ-4-1 with AS-TLC. The fluorescent substance at the center of the circular spots is CPJ-4-1; "+" indicates the mixture containing the inhibitor Tagtociclib, and "-" indicates the mixture without inhibitor. **c** Screening and identification of Tagtociclib. Red arrow indicates experimental group including 164-A02. Yellow arrow indicates experimental group where CPJ-4-1 was significantly reduced. White dotted lines connect standard compounds in the mixture that match with the R*_f_* value and color of Tagtociclib. White arrows indicate Tagtociclib.

**Supplementary Fig. 9** Screening and validation of YTHDC1 hit compounds. **a** Feasibility assessment of the HTRF-based mixture screening strategy using compound **25**. The red dot indicates the mixture containing compound **25**. **b** Compound **25** was identified from the candidate mixture by AS-TLC analysis. White dotted lines connect standard compounds in the mixture that exhibit identical Rf values and coloration to compound **25**. White arrows indicate compound **25** in 16 monomer compounds resolved in the development. **c** Replicate validation of the initial 16 candidate mixtures. Red dots indicate candidate mixture retained inhibitory activity upon retesting, blue dot indicates the mixture containing compound **25**. **d** Preliminary assessment of compound binding to YTHDC1. Compound **25** was recovered from mixture 6, and indicated with standard compound by white dotted lines. The developing agent was petroleum ether: ethyl acetate (2:1, v/v). **e** A candidate compound was identified in mixture 3, as marked by a white arrow. The developing agent was ethyl acetate. **f** Hit compound 168-G04 was identified through AS-TLC. White dotted lines link standard compounds in the mixture that share the same Rf value and color with 168-G04. White arrows denote 168-G04 retrieved after YTHDC1 binding and the corresponding standard, both developed in the ethyl acetate. **g** AS-TLC validation of 168-G04 binding to YTHDC1. 168-G04 was incubated with YTHDC1 at concentrations of 50 μM, 100 μM, and 200 μM. **h** In the AlphaScreen assay, 168-G04 exhibited an IC_50_ value greater than 100 μM. **i** BLI analysis revealed that the dissociation constant (K_d_) between YTHDC1 and 168-G04 is 71.8 μM. **j** Results of the HTRF screening of 480 individual compounds. Red dots represent four hit compounds that showed inhibition rates exceeding 70% after correction for fluorescence interference. **k-m** Binding of the 167-D08, 168-H04, and 180-E09 to YTHDC1 were further evaluated by BLI. Compounds exhibited K_d_ values exceeding 100 μM. **n** Inhibitory activity of compounds 167-D08, 168-H04, and 180-E09 against YTHDC1 at concentrations of 50 μM, 25 μM, and 12.5 μM, as assessed by HTRF assay.

**Supplementary Table 1** Fluorescence intensity values at 620 nm and 665 nm for 91 sets of HTRF data*^a^*.

| Group | Fluorescence ratio | 665 nm fluorescence | 620 nm  fluorescence | Group | Fluorescence ratio | 665 nm fluorescence | 620 nm  fluorescence |
| --- | --- | --- | --- | --- | --- | --- | --- |
| 1 | 0.794520548 | 232 | 292 | 46 | 0.918215613 | 247 | 269 |
| 2 | 1.110091743 | 242 | 218 | 47 | 0.878661088 | 210 | 239 |
| 3 | 1.647058824 | 224 | 136 | 48 | 0.622259696 | 369 | 593 |
| 4 | 1.327014218 | 280 | 211 | 49 | 0.958015267 | 251 | 262 |
| 5 | 0.944186047 | 203 | 215 | 50 | 1.554621849 | 185 | 119 |
| 6 | 1.451612903 | 270 | 186 | 51 | 0.862500000 | 207 | 240 |
| 7 | 1.122222222 | 202 | 180 | 52 | 1.019011407 | 268 | 263 |
| 8 | 1.086142322 | 290 | 267 | 53 | 0.935185185 | 202 | 216 |
| 9 | 1.165876777 | 246 | 211 | 54 | 0.657446809 | 309 | 470 |
| 10 | 1.511111111 | 272 | 180 | 55 | 0.825783972 | 237 | 287 |
| 11 | 0.865384615 | 225 | 260 | 56 | 1.085365854 | 178 | 164 |
| 12 | 1.758928571 | 197 | 112 | 57 | 0.99378882 | 160 | 161 |
| 13 | 1.151315789 | 175 | 152 | 58 | 1.015151515 | 268 | 264 |
| 14 | 1.443113772 | 241 | 167 | 59 | 0.886363636 | 195 | 220 |
| 15 | 1.37654321 | 223 | 162 | 60 | 1.178343949 | 185 | 157 |
| 16 | 1.456375839 | 217 | 149 | 61 | 0.801843318 | 174 | 217 |
| 17 | 1.696808511 | 319 | 188 | 62 | 1.241025641 | 242 | 195 |
| 18 | 1.447204969 | 233 | 161 | 63 | 0.980544747 | 252 | 257 |
| 19 | 0.677152318 | 409 | 604 | 64 | 1.189054726 | 239 | 201 |
| 20 | 0.944785276 | 308 | 326 | 65 | 1.200913242 | 263 | 219 |
| 21 | 1.552083333 | 298 | 192 | 66 | 0.975778547 | 282 | 289 |
| 22 | 1.041420118 | 176 | 169 | 67 | 0.879562044 | 241 | 274 |
| 23 | 0.882352941 | 180 | 204 | 68 | 0.707746479 | 201 | 284 |
| 24 | 1.258883249 | 248 | 197 | 69 | 1.187500000 | 152 | 128 |
| 25 | 0.602627258 | 367 | 609 | 70 | 0.800000000 | 136 | 170 |
| 26 | 0.682779456 | 226 | 331 | 71 | 1.420454545 | 125 | 88 |
| 27 | 0.774193548 | 336 | 434 | 72 | 1.366336634 | 138 | 101 |
| 28 | 1.161904762 | 244 | 210 | 73 | 1.012738854 | 318 | 314 |
| 29 | 0.956521739 | 198 | 207 | 74 | 0.797014925 | 267 | 335 |
| 30 | 0.614107884 | 148 | 241 | 75 | 0.57299843 | 365 | 637 |
| 31 | 1.06374502 | 267 | 251 | 76 | 0.644113667 | 476 | 739 |
| 32 | 1.305194805 | 201 | 154 | 77 | 0.506072874 | 375 | 741 |
| 33 | 0.704545455 | 310 | 440 | 78 | 0.690661479 | 355 | 514 |
| 34 | 1.562500000 | 250 | 160 | 79 | 0.750656168 | 286 | 381 |
| 35 | 1.043668122 | 239 | 229 | 80 | 0.588235294 | 130 | 221 |
| 36 | 1.137142857 | 199 | 175 | 81 | 1.279503106 | 206 | 161 |
| 37 | 0.683333333 | 328 | 480 | 82 | 0.597328244 | 313 | 524 |
| 38 | 0.864035088 | 197 | 228 | 83 | 1.350746269 | 181 | 134 |
| 39 | 0.754475703 | 295 | 391 | 84 | 1.099337748 | 166 | 151 |
| 40 | 0.947154472 | 233 | 246 | 85 | 0.915789474 | 174 | 190 |
| 41 | 1.021276596 | 192 | 188 | 86 | 0.814009662 | 337 | 414 |
| 42 | 1.055900621 | 170 | 161 | 87 | 1.182857143 | 207 | 175 |
| 43 | 0.536209553 | 348 | 649 | 88 | 0.861344538 | 205 | 238 |
| 44 | 0.771028037 | 330 | 428 | 89 | 1.00990099 | 204 | 202 |
| 45 | 0.604010025 | 241 | 399 | 90 | 0.384721266 | 559 | 1453 |
|  |  |  |  | 91 | 0.322580645 | 50 | 155 |

*a* A total of 38 candidate groups marked in blue and red exhibited fluorescence ratios below 0.9, including 22 blue-labeled groups with abnormally high fluorescence intensity (>300) at 625 nm and 16 red-labeled groups proceeding to further validation.

**Supplementary Table 2** Analytical purity of 498 azide derivatives from the click chemistry library.*^a^*

|   P1-A2  98% |   P1-A7  45% |   P1-A11  69% |   P2-A2  7% |   P2-A3  0 |   P2-A10  6% | P3-A2  87% |   P3-A9  29% |
| --- | --- | --- | --- | --- | --- | --- | --- |
|   P1-B2  91% |   P1-B7  0 |   P1-B11  5% |   P2-B2  31% |   P2-B3  0 |   P2-B10  12% |   P3-B2  81% |   P3-B9  17% |
|   P1-C2  3% |   P1-C7  0 |   P1-C11  0 |   P2-C2  59% |   P2-C3  78% |   P2-C10  29% |   P3-C2  68% |   P3-C9  17% |
|   P1-D2  0 |   P1-D7  31% |   P1-D11  49% |   P2-D2  62% |   P2-D3  46% |   P2-D10  52% |   P3-D2  0 |   P3-D9  23% |
|   P1-E2  21% |   P1-E7  85% | P1-E11  8% |   P2-E2  9% |   P2-E3  20% |   P2-E10  47% |   P3-E2  74% |   P3-E9  42% |
|   P1-F2  19% |   P1-F7  68% |   P1-F11  0 |   P2-F2  41% |   P2-F3  7% |   P2-F10  41% |   P3-F2  2% |   P3-F9  15% |
|   P1-G2  46% |   P1-G7  4% |   P1-G11  47% |   P2-G2  11% |   P2-G3  17% |   P2-G10  0 |   P3-G2  39% |   P3-G9  25% |
|   P1-H2  3% |   P1-H7  97% |   P1-H11  17% |   P2-H2  8% |   P2-H3  74% |   P2-H10  45% |   P3-H2  19% |   P3-H9  34% |
|   P3-A11  0 |   P4-A2  9% |   P4-A3  1% |   P4-A4  6% |   P5-D2  0 | P5-D10  0 |   P6-A8  55% |   P6-A9  29% |
|   P3-B11  66% |   P4-B2  8% |   P4-B3  6% |   P4-B4  9% |   P5-D3  0 |   P5-D11  0 |   P6-A9  88% |   P6-B9  90% |
|   P3-C11  11% |   P4-C2  0 |   P4-C3  20% |   P4-C4  6% |   P5-D4  10% |   P5-A4  4% |   P6-E8  60% |   P6-D9  30% |
|   P3-D11  60% |   P4-D2  3% |   P4-D3  0 |   P4-D4  0 |   P5-D5  23% |   P5-B4  93% |   P6-F8  63% |   P6-E9  2% |
|   P3-E11  39% |   P4-E2  36% |   P4-E3  7% | P4-E4  40% | P5-D6  18% | P5-C4  5% | P6-G8  67% | P6-F9  58% |
| P3-F11  29% | P4-F2  0 | P4-F3  5% | P4-F4  86% | P5-D7  11% | P6-C8  11% | P6-H8  16% | P6-G9  0 |
| P3-G11  0 | P4-G2  23% | P4-G3  14% | P4-G4  0 | P5-D8  0 | P6-D8  0 | P6-C9  0 | P6-H9  0 |
| P3-H11  0 | P4-H2  40% | P4-H3  25% | P4-H4  56% | P5-D9  12% | P7-A2  0 | P7-B2  88% | P7-C2  11% |
| P7-D2  98% | P7-E2  91% | P7-F2  93% | P7-G2  98% | P7-H2  33% | P6-A5  0 | P6-B5  58% | P6-C5  91% |
| P6-D5  90% | P6-E5  5% | P6-F5  86% | P6-G5  2% | P6-H5  9% | P7-A7  0 | P7-B7  98% | P7-C7  97% |
| P7-D7  0 | P7-E7  97% | P7-F7  10% | P7-G7  96% | P7-H7  99% | P7-A5  94% | P7-B5  0 | P7-C5  87% |
| P7-D5  16% | P7-E5  92% | P7-F5  2% | P7-G5  90% | P7-H5  99% | P8-A3  87% | P8-B3  7% | P8-C3  7% |
| P8-D3  79% | P8-E3  0 | P8-F3  29% | P8-G3  61% | P8-H3  92% | P8-A8  99% | P8-B8  94% | P8-C8  0 |
| P8-D8  49% | P8-E8  92% | P8-F8  99% | P8-G8  98% | P8-H8  99% | P8-A9  53% | P8-B9  18% | P8-C9  30% |
| P8-D9  0 | P8-E9  83% | P8-F9  0 | P8-G9  37% | P8-H9  22% | P9-A2  30% | P9-B2  64% | P9-C2  84% |
| P9-D2  7% | P9-E2  99% | P9-F2  77% | P9-G2  75% | P9-H2  52% | P9-A5  79% | P9-B5  0 | P9-C5  62% |
| P9-D5  4% | P9-E5  0 | P9-F5  36% | P9-G5  79% | P9-H5  50% | P9-A9  0 | P9-B9  99% | P9-C9  99% |
| P9-D9  99% | P9-E9  99% | P9-F9  86% | P9-G9  82% | P9-H9  99% | P10-A2  79% | P10-B2  48% | P10-C2  76% |
| P10-D2  0 | P10-E2  23% | P10-F2  73% | P10-G2  13% | P10-H2  84% | P10-A5  73% | P10-B5  0 | P10-C5  0 |
| P10-D5  8% | P10-E5  3% | P10-F5  70% | P10-G5  5% | P10-H5  63% | P10-A7  68% | P10-B7  0 | P10-C7  73% |
| P10-D7  31% | P10-E7  18% | P10-F7  5% | P10-G7  26% | P10-H7  55% | P11-A3  37% | P11-B3  0 | P11-C3  57% |
| P11-D3  81% | P11-E3  52% | P11-F3  28% | P11-G3  56% | P11-H3  0 | P11-A5  92% | P11-B5  95% | P11-C5  15% |
| P11-D5  84% | P11-E5  68% | P11-F5  56% | P11-G5  89% | P11-H5  81% | P11-A7  75% | P11-B7  71% | P11-C7  36% |
| P11-D7  85% | P11-E7  86% | P11-F7  12% | P11-G7  29% | P11-H7  69% | P12-A4  82% | P12-B4  90% | P12-C4  0 |
| P12-D4  89% | P12-E4  90% | P12-F4  35% | P12-G4  61% | P12-H4  50% | P12-A8  87% | P12-B8  66% | P12-B10  98% |
| P12-C8  12% | P12-D8  44% | P12-E8  70% | P12-F8  82% | P12-G8  67% | P12-H8  20% | P12-A10  27% | P13-B2  40% |
| P12-C10  0 | P12-D10  0 | P12-E10  61% | P12-F10  97% | P12-G10  92% | P12-H10  74% | P13-A2  30% | P13-C2  79% |
| P13-D2  0 | P13-E2  64% | P13-F2  80% | P13-G2  43% | P13-H2  18% | P13-A4  25% | P13-B4  81% | P13-C4  71% |
| P13-D4  95% | P13-E4  52% | P13-F4  62% | P13-G4  23% | P13-H4  4% | P13-A3  90% | P13-B3  98% | P13-C3  98% |
| P13-D3  94% | P13-E3  32% | P13-F3  98% | P13-G3  0 | P13-H3  25% | P14-A4  44% | P14-B4  10% | P14-C4  36% |
| P14-D4  72% | P14-E4  41% | P14-F4  10% | P14-G4  41% | P14-H4  22% | P14-A7  37% | P14-B7  0 | P14-C7  0 |
| P14-D7  88% | P14-E7  53% | P14-F7  27% | P14-G7  17% | P14-H7  28% | P14-A9  93% | P14-B9  96% | P14-C9  84% |
| P14-D9  26% | P14-E9  76% | P14-F9  98% | P14-G9  59% | P14-H9  92% | P15-A4  35% | P15-B4  37% | P15-C4  34% |
| P15-D4  24% | P15-E4  33% | P15-F4  30% | P15-G4  10% | P15-H4  0 | P15-A5  93% | P15-B5  96% | P15-C5  93% |
| P15-D5  87% | P15-E5  89% | P15-F5  28% | P15-G5  93% | P15-H5  98% | P15-A8  10% | P15-B8  89% | P15-C8  34% |
| P15-D8  0 | P15-E8  53% | P15-F8  66% | P15-G8  28% | P15-H8  59% | P16-E2  76% | P16-E3  30% | P16-E4  58% |
| P16-E5  62% | P16-E6  56% | P16-E7  15% | P16-E8  46% | P16-E9  0 | P16-E10  26% | P16-E11  6% | P16-A6  99% |
| P16-B6  98% | P16-C6  0 | P16-D6  97% | P16-E6  98% | P16-F6  98% | P16-G6  17% | P17-A2  0 | P17-B2  37% |
| P17-C2  76% | P17-D2  47% | P17-E2  0 | P17-F2  46% | P17-G2  61% | P17-H2  67% | P17-A7  23% | P17-B7  87% |
| P17-C7  97% | P17-D7  98% | P17-E7  90% | P17-F7  77% | P17-G7  33% | P17-H7  98% | P17-A10  95% | P17-B10  25% |
| P17-C10  53% | P17-D10  84% | P17-E10  33% | P17-F10  82% | P17-G10  68% | P17-H10  82% | P18-A4  42% | P18-B4  93% |
| P18-C4  84% | P18-D4  78% | P18-E4  10% | P18-F4  40% | P18-G4  47% | P18-H4  69% | P18-A7  87% | P18-B7  11% |
| P18-C7  55% | P18-D7  43% | P18-E7  7% | P18-F7  34% | P18-G7  8% | P18-H7  3% | P18-A8  93% | P18-B8  12% |
| P18-C8  69% | P18-D8  83% | P18-E8  0 | P18-F8  0 | P18-G8  70% | P18-H8  10% | P19-B2  20% | P19-B3  7% |
| P19-B4  0 | P19-B5  73% | P19-B6  35% | P19-B7  37% | P19-B8  29% | P19-B9  78% | P19-B10  20% | P19-B11  43% |
| P19-A4  97% | P19-B4  0 | P19-C4  0 | P19-D4  74% | P19-E4  96% | P19-F4  71% | P20-G2  99% | P20-H2  81% |
| P20-A2  96% | P20-B2  99% | P20-C2  90% | P20-D2  77% | P20-E2  99% | P20-F2  99% | P20-A4  39% | P20-B4  98% |
| P20-C4  99% | P20-D4  97% | P20-E4  69% | P20-F4  93% | P20-G4  69% | P20-H4  97% | P20-A10  19% | P20-B10  73% |
| P20-C10  6% | P20-D10  6% | P20-E10  66% | P20-F10  34% | P20-G10  59% | P20-H10  33% | P21-A2  98% | P21-B2  80% |
| P21-C2  99% | P21-D2  72% | P21-E2  98% | P21-F2  96% | P21-G2  92% | P21-H2  98% | P21-A4  86% | P21-B4  99% |
| P21-C4  99% | P21-D4  99% | P21-E4  94% | P21-F4  98% | P21-G4  98% | P21-H4  37% | P21-A8  99% | P21-B8  98% |
| P21-C8  86% | P21-D8  74% | P21-E8  75% | P21-F8  60% | P21-G8  95% | P21-H8  99% | P22-A2  59% | P22-A3  57% |
| P22-A4  58% | P22-A5  58% | P22-A6  65% | P22-A7  50% | P22-A8  50% | P22-A9  0 | P22-A10  13% | P22-A11  11% |
| P22-B2  34% | P22-B3  99% | P22-B4  87% | P22-B5  22% | P22-B6  65% | P22-B7  99% | P22-B8  99% | P22-B9  87% |
| P22-B10  99% | P22-B11  99% |  |  |  |  |  |  |

*^a^* Compounds are numbered according to the position of their microtiter plate wells, e.g. **P1-A2** refers to the compound from sub-library, **P**late **1**, 96-well microtiter plate position of **A2**

**Supplementary Fig. 10.** Overview of reaction outcomes and yield distribution in the sampled library (a) Product detection status of 498 compounds randomly sampled from 1636 total high-throughput reactions. The sampled set included 415 reactions with detectable target product (green) and 83 reactions without detectable product (beige). (b) Reaction yield distribution among the 415 product-positive compounds. Reactions were grouped into four yield ranges: >90% (n = 101, green), 70-90% (n = 80, blue), 50-70% (n = 66, yellow), and <50% (n = 168, brown).

**UPLC-MS result of crude P1-E7.**

**UPLC-MS result of crude P2-C3.**

**UPLC-MS result of crude P3-A2**

**UPLC-MS result of crude P4-F4**

**UPLC-MS result of crude P6-B9**

**UPLC-MS result of crude P7-F2**

**UPLC-MS result of crude P9-H9.**

**UPLC-MS result of crude P10-A3.**

**UPLC-MS result of crude P11-D3.**

**UPLC-MS result of crude P12-A4.**

**UPLC-MS result of crude P13-B3.**

**UPLC-MS result of crude P13-D3.**

**UPLC-MS result of crude P14-B9**

**UPLC-MS result of crude P14-F9.**

**UPLC-MS result of crude P15-A5.**

**UPLC-MS result of crude P15-H5.**

**UPLC-MS result of crude P16-D6.**

**UPLC-MS result of crude P16-F6.**

**UPLC-MS result of crude P17-B7.**

**UPLC-MS result of crude P20-A2.**

**Supplementary Fig.11 UPLC-MS results of crude products P1-E7, P2-C3, P3-A2, P4-F4, P6-B9, P7-F2, P9-H9, P10-A3, P11-D3, P12-A4, P13-B3, P13-D3, P14-B9, P14-F9, P15-A5, P15-H5, P16-D6, P16-F6, P17-B7, P20-A2.**

**Supplementary Fig. 12** Primary screening of the click chemistry library. **a** Structure of YTHDC1 fluorescent probe **FP-YD**. **b** YTHDC1 was treated with increasing concentrations of compound **25** and corresponding DMSO controls; protein activity was subsequently assessed by fluorescence polarization (FP) assay. Data represent mean ± SD of triplicate measurements from four biologically independent experiments. **c** Primary screening inhibition profile of the click-chemistry library at 5 μM concentration via FP assay. Red dots indicated compounds with inhibition against YTHDC1greater than 95%.

**Supplementary Table 3** YTHDC1 inhibition rate of the click-library plate 1~9 in first round screening*.*

| **Sub-library C1** Inhibition Rate (%) *^a^* | | | | | | | | | |
| --- | --- | --- | --- | --- | --- | --- | --- | --- | --- |
| **Plate** | **1** | **2** | **3** | **4** | **5** | **6** | **7** | **8** | **9** |
| **A02** | 81.18 | 45.24 | 73.95 | 35.38 | 23.53 | 59.80 | 54.23 | 50.51 | 42.78 |
| **A03** | 27.03 | 53.19 | 71.32 | 56.61 | 51.88 | 75.71 | 28.78 | 54.71 | 31.01 |
| **A04** | 49.85 | 64.40 | 77.84 | 29.10 | 43.00 | 61.42 | 41.38 | 52.45 | 60.75 |
| **A05** | 90.72 | 56.53 | 54.31 | 84.92 | 57.70 | 51.97 | 45.83 | 47.68 | 58.71 |
| **A06** | 53.27 | 50.25 | 47.71 | 29.58 | 30.48 | 36.78 | 49.06 | 42.76 | 77.86 |
| **A07** | 83.17 | 77.52 | 88.50 | 75.06 | 50.67 | 38.40 | 71.67 | 72.89 | 35.50 |
| **A08** | 25.44 | 47.23 | 66.87 | 21.63 | 46.47 | 31.21 | 49.46 | 39.28 | 20.76 |
| **A09** | 27.83 | 54.78 | 42.46 | 51.05 | 17.31 | 52.53 | 71.03 | 43.65 | 25.42 |
| **A10** | 23.06 | 50.41 | 40.15 | 22.42 | 4.55 | 29.59 | 47.04 | 30.96 | 47.02 |
| **A11** | 54.62 | 72.75 | 53.67 | 21.71 | 10.93 | 101.56 | 44.86 | 30.24 | 36.35 |
| **B02** | 39.52 | 38.56 | 79.51 | 16.06 | 27.73 | 30.88 | 31.69 | 57.70 | 39.90 |
| **B03** | 26.64 | 53.75 | 81.42 | 42.14 | 32.98 | 64.73 | 25.88 | 49.30 | 38.38 |
| **B04** | 48.82 | 53.51 | 89.85 | 36.81 | 77.25 | 27.09 | 68.12 | 62.71 | 32.70 |
| **B05** | 57.96 | 30.85 | 56.22 | 48.82 | 53.58 | 87.99 | 37.43 | 73.37 | 58.54 |
| **B06** | 30.93 | 66.63 | 61.94 | 54.47 | 22.56 | 39.36 | 19.90 | 45.10 | 27.87 |
| **B07** | 32.12 | 21.55 | 59.40 | 18.92 | 50.27 | 79.51 | 50.67 | 35.08 | 73.54 |
| **B08** | 28.62 | 41.74 | 53.19 | 36.10 | 53.26 | 35.49 | 48.17 | 9.80 | 56.17 |
| **B09** | 57.33 | 81.34 | 66.00 | 32.36 | 26.76 | 50.83 | 51.00 | 48.41 | 55.92 |
| **B10** | 27.75 | 24.17 | 74.58 | 77.45 | 26.28 | 26.52 | 50.43 | 34.36 | 51.93 |
| **B11** | 28.94 | 67.43 | 91.28 | 32.28 | 19.90 | 77.89 | 41.79 | 43.40 | 53.54 |
| **C02** | 30.21 | 61.86 | 28.86 | 48.74 | 43.16 | 13.68 | 36.21 | 36.21 | 52.44 |
| **C03** | 92.55 | 92.63 | 25.28 | 57.17 | 43.89 | 37.43 | 29.11 | 51.64 | 57.36 |
| **C04** | 94.38 | 26.56 | 53.43 | 59.71 | 39.20 | 43.08 | 40.66 | 46.72 | 65.15 |
| **C05** | 38.72 | 44.92 | 24.09 | 17.02 | 13.19 | 64.81 | 35.49 | 55.92 | 45.33 |
| **C06** | 25.52 | 27.91 | 44.53 | 38.32 | 45.75 | 35.33 | 27.89 | 57.14 | 53.71 |
| **C07** | 38.56 | 33.79 | 38.48 | 39.36 | 42.68 | 79.99 | 33.71 | 48.17 | 58.12 |
| **C08** | 38.48 | 35.38 | 39.20 | 26.88 | 2.53 | 53.10 | 48.49 | 58.75 | 43.72 |
| **C09** | 25.13 | 55.74 | 31.01 | 33.87 | 17.07 | 48.49 | 44.94 | 25.23 | 56.09 |
| **C10** | 20.12 | 85.16 | 51.52 | 25.52 | 38.72 | 27.81 | 75.15 | 43.89 | 63.29 |
| **C11** | 44.05 | 95.73 | 37.21 | 34.67 | 42.92 | 39.12 | 49.46 | 22.64 | 38.89 |
| **D02** | 89.45 | 65.68 | 45.00 | 36.26 | 46.15 | 65.05 | 49.70 | 44.29 | 47.19 |
| **D03** | 37.53 | 87.15 | 31.25 | 34.35 | 45.50 | 39.61 | 32.01 | 46.80 | 34.65 |
| **D04** | 45.48 | 35.86 | 39.36 | 14.71 | -1.67 | 27.98 | 45.42 | 15.78 | 57.70 |
| **D05** | 34.27 | 62.18 | 45.48 | 54.78 | 15.54 | 30.24 | 69.17 | 53.10 | 41.51 |
| **D06** | 43.57 | 40.07 | 52.80 | 39.99 | 39.04 | 46.23 | 79.11 | 42.92 | 56.17 |
| **D07** | 71.24 | 8.27 | 80.55 | 45.24 | 31.21 | 1.56 | 43.65 | 34.44 | 60.15 |
| **D08** | 48.42 | 74.50 | 92.23 | 25.84 | 6.73 | 43.81 | 53.26 | 36.62 | 49.82 |
| **D09** | 64.56 | 42.86 | 35.86 | 30.85 | 0.92 | 48.41 | 47.20 | 49.78 | 51.00 |
| **D10** | 84.12 | 39.84 | 65.60 | 34.43 | 4.07 | 18.52 | 41.63 | 40.01 | 56.09 |
| **D11** | 65.44 | 32.76 | 52.08 | 39.20 | 33.06 | 52.37 | 50.83 | 70.54 | 37.02 |
| **E02** | 29.50 | 28.39 | 97.16 | 33.00 | 22.08 | 54.55 | 33.87 | 50.51 | 39.23 |
| **E03** | 47.15 | 43.89 | 37.77 | 47.39 | 42.03 | 45.26 | 29.11 | 48.73 | 75.74 |
| **E04** | 34.19 | 53.11 | 36.73 | 43.73 | 31.53 | 81.69 | 27.33 | 47.44 | 39.06 |
| **E05** | 73.31 | 41.35 | 52.88 | 28.70 | 19.01 | 39.45 | 27.81 | 60.61 | 61.93 |
| **E06** | 33.16 | 73.23 | 34.35 | 44.84 | 19.41 | 33.31 | 34.28 | 52.29 | 50.66 |
| **E07** | 94.62 | 48.58 | 71.16 | 20.43 | 26.36 | 49.62 | 36.21 | 51.32 | 61.85 |
| **E08** | 32.68 | 65.52 | 5.33 | 52.48 | 27.09 | 39.93 | 60.61 | 57.94 | 84.13 |
| **E09** | 34.75 | 37.13 | 62.74 | 47.39 | 22.56 | 22.40 | 47.52 | 55.68 | 59.14 |
| **E10** | 85.08 | 28.78 | 25.13 | 63.61 | 26.12 | 19.25 | 40.25 | 40.74 | 49.48 |
| **E11** | 53.19 | 38.01 | 21.23 | 75.38 | 27.81 | 39.77 | 43.73 | 54.23 | 57.27 |
| **F02** | 3.82 | 62.81 | 52.00 | 28.07 | 44.29 | 30.40 | 39.36 | 62.06 | 62.10 |
| **F03** | 33.47 | 46.04 | 69.02 | 48.90 | 39.28 | 39.20 | 41.46 | 62.14 | 61.34 |
| **F04** | 48.50 | 48.90 | 42.14 | 6.68 | 42.27 | 43.48 | 33.63 | 66.10 | 25.84 |
| **F05** | 58.60 | 63.29 | 60.91 | 9.54 | 48.65 | 29.59 | 46.15 | 54.31 | 47.87 |
| **F06** | 21.23 | 51.76 | 51.29 | 46.91 | 35.41 | 8.59 | 27.41 | 61.25 | 69.64 |
| **F07** | 70.45 | 40.23 | 53.51 | 30.77 | 22.48 | 33.14 | 38.48 | 72.89 | 67.01 |
| **F08** | 38.64 | 51.36 | 41.35 | 34.19 | 26.76 | 2.77 | 16.67 | 88.48 | 59.98 |
| **F09** | 49.77 | 56.06 | 62.34 | 32.28 | 32.82 | 6.97 | 31.37 | 62.39 | 64.05 |
| **F10** | 57.81 | 34.35 | 43.65 | 19.88 | 22.16 | 25.23 | 39.28 | 55.52 | 34.99 |
| **F11** | 44.92 | 30.14 | 25.76 | 33.16 | 20.38 | 88.56 | 12.63 | 26.12 | 80.49 |
| **G02** | 46.59 | 12.72 | 59.71 | 47.23 | 45.50 | 48.01 | 52.21 | 37.91 | 65.91 |
| **G03** | 63.61 | 29.10 | 15.35 | 45.48 | 32.42 | 14.81 | 32.50 | 50.11 | 38.46 |
| **G04** | 46.36 | 52.24 | 33.79 | 17.17 | 27.57 | 51.64 | 52.37 | 50.59 | 83.37 |
| **G05** | 72.12 | 55.34 | 57.57 | 17.65 | 30.64 | 37.75 | 52.77 | 80.08 | 83.37 |
| **G06** | 94.14 | 31.49 | 51.13 | 97.80 | 28.62 | 48.41 | 40.58 | 36.62 | 61.59 |
| **G07** | 61.30 | 33.63 | 45.96 | 49.85 | 14.81 | 37.75 | 37.99 | 54.39 | 58.97 |
| **G08** | 88.02 | 43.97 | 36.97 | 25.68 | 27.57 | 36.70 | 53.50 | 59.72 | 54.05 |
| **G09** | 63.93 | 53.59 | 50.17 | 46.83 | 21.51 | 39.85 | 34.84 | 56.33 | 44.90 |
| **G10** | 67.90 | 45.00 | 38.96 | 28.15 | 30.72 | 29.03 | 41.22 | 56.65 | 71.00 |
| **G11** | 29.82 | 17.57 | 38.01 | 30.29 | 16.18 | 44.37 | 50.92 | 46.80 | 28.72 |
| **H02** | 32.84 | 43.49 | -0.64 | 56.61 | 28.78 | 56.81 | 45.10 | 65.78 | 19.74 |
| **H03** | 53.43 | 32.28 | 33.87 | 22.50 | 21.51 | 16.83 | 45.91 | 46.80 | 57.61 |
| **H04** | 43.25 | 49.46 | 42.94 | 2.94 | 21.19 | 40.98 | 37.26 | 53.02 | 34.48 |
| **H05** | 39.92 | 33.71 | 45.48 | 5.96 | 23.61 | 42.60 | 41.71 | 54.71 | 46.34 |
| **H06** | 54.39 | 30.29 | 11.53 | 34.91 | 40.50 | 41.71 | 39.20 | 13.68 | 69.47 |
| **H07** | 92.31 | 59.87 | 25.68 | 38.72 | 27.65 | 61.58 | 75.07 | 51.97 | 74.05 |
| **H08** | 58.12 | 32.76 | 18.69 | 33.00 | 21.43 | 42.19 | 33.87 | 63.03 | 59.81 |
| **H09** | 86.67 | 90.41 | 38.72 | 29.66 | 21.76 | 24.02 | 21.43 | 60.12 | 72.27 |
| **H10** | 87.07 | 41.27 | 46.04 | 8.11 | 25.88 | 35.16 | 21.92 | 59.15 | 80.06 |
| **H11** | 79.67 | 23.38 | 31.25 | 31.73 | 18.12 | 16.02 | 0.03 | 41.71 | 52.19 |

*^a^* Negative control was added to the first and 12th columns of the screened 384-well microtiter plates, the wells exhibiting the top twenty inhibition rates were highlighted in red.

**Supplementary Table 4** YTHDC1 inhibition rate of the click-library plate 10~17 in first round screening*.*

| **Sub-library C1** Inhibition Rate (%) *^a^* | | | | | | | | |
| --- | --- | --- | --- | --- | --- | --- | --- | --- |
| **Plate** | **10** | **11** | **12** | **13** | **14** | **15** | **16** | **17** |
| **A02** | 46.68 | 55.49 | 34.74 | 55.72 | 63.05 | 30.42 | 47.54 | 48.54 |
| **A03** | 67.27 | 63.29 | 49.56 | 44.82 | 61.26 | 57.09 | 49.42 | 48.63 |
| **A04** | 43.63 | 46.00 | 11.44 | 31.78 | 53.08 | 13.46 | 12.27 | 63.46 |
| **A05** | 77.10 | 53.54 | 48.97 | 39.62 | 35.19 | 53.76 | 41.41 | 56.17 |
| **A06** | 57.36 | 45.33 | 65.91 | 29.14 | 49.50 | 55.30 | 67.40 | 79.33 |
| **A07** | 38.46 | 78.88 | 81.50 | 27.52 | 62.79 | 57.51 | 9.46 | 69.47 |
| **A08** | 45.33 | 84.38 | 44.22 | 59.05 | 40.81 | 42.26 | 3.83 | 66.90 |
| **A09** | 70.32 | 49.05 | 38.72 | 23.18 | 47.46 | 66.03 | 5.37 | 36.71 |
| **A10** | 44.90 | 8.22 | 43.72 | 76.51 | 48.31 | 64.16 | 10.05 | 53.43 |
| **A11** | 69.64 | 88.45 | 65.49 | 63.99 | 35.19 | 47.80 | 21.81 | 46.66 |
| **B02** | 60.24 | 53.54 | 61.68 | 22.66 | 51.46 | 50.53 | 16.96 | 38.77 |
| **B03** | 69.56 | 59.22 | 51.60 | 10.82 | 28.54 | 49.08 | 49.84 | 48.46 |
| **B04** | 59.90 | 38.89 | 50.58 | 54.62 | 49.93 | 53.85 | 36.98 | 55.40 |
| **B05** | 68.62 | 52.10 | 68.37 | 40.98 | 58.45 | 17.72 | 28.03 | 35.59 |
| **B06** | 70.83 | 64.64 | 42.11 | 43.54 | 61.43 | 40.39 | 48.99 | 52.32 |
| **B07** | 61.76 | 21.69 | 52.95 | 62.28 | 42.18 | 61.94 | 43.03 | 60.03 |
| **B08** | 65.24 | 32.02 | 50.07 | 65.35 | 43.45 | 76.51 | 12.27 | 56.26 |
| **B09** | 70.15 | 65.15 | 29.23 | 38.51 | 60.15 | 90.74 | 46.86 | 72.13 |
| **B10** | 53.29 | 49.65 | 56.59 | 54.96 | 55.98 | 71.49 | 62.54 | 76.67 |
| **B11** | 62.27 | 46.68 | 53.12 | 63.65 | 43.71 | 45.75 | 68.50 | 48.97 |
| **C02** | 59.64 | 73.03 | 59.14 | 56.40 | 59.22 | 23.94 | 50.44 | 54.37 |
| **C03** | 65.66 | 77.44 | 53.88 | 27.69 | 63.90 | 42.43 | 39.36 | 65.52 |
| **C04** | 66.76 | 77.61 | 50.92 | 61.01 | 51.46 | 63.22 | -6.48 | 55.40 |
| **C05** | 55.07 | 96.75 | 46.34 | 59.73 | 41.15 | 98.49 | 44.56 | 56.95 |
| **C06** | 58.71 | 64.13 | 65.07 | 83.93 | 27.78 | 30.67 | 37.57 | 54.80 |
| **C07** | 63.54 | 49.82 | 59.81 | 43.71 | 53.17 | 47.46 | 29.91 | 60.12 |
| **C08** | 63.37 | 42.11 | 41.01 | 66.29 | 40.90 | 34.34 | 16.44 | 62.35 |
| **C09** | 44.14 | 69.64 | 69.39 | 61.09 | 43.45 | 57.51 | 32.46 | 53.09 |
| **C10** | 66.42 | 50.49 | 28.72 | 10.31 | 15.08 | 5.37 | 42.09 | 30.27 |
| **C11** | 62.10 | 54.82 | 63.37 | 42.86 | 52.66 | -8.18 | 16.27 | 50.69 |
| **D02** | 51.09 | 34.40 | 64.47 | 59.90 | 26.50 | 27.52 | 26.92 | 38.94 |
| **D03** | 49.48 | 64.05 | 60.75 | 57.17 | 66.54 | 20.36 | 48.65 | 60.29 |
| **D04** | 53.97 | 66.42 | 57.27 | 45.75 | 55.89 | 35.44 | 30.84 | 53.00 |
| **D05** | 37.28 | 67.44 | 39.48 | 13.89 | 65.18 | 68.84 | 9.46 | 61.23 |
| **D06** | 33.80 | 68.03 | 47.70 | 46.35 | 61.35 | 55.64 | 34.25 | 48.37 |
| **D07** | 50.49 | 86.42 | 35.41 | 51.55 | 53.08 | 13.29 | 8.78 | 74.53 |
| **D08** | 41.34 | 79.21 | 38.38 | 51.12 | 65.69 | 68.93 | 53.25 | 19.90 |
| **D09** | 27.11 | 56.09 | 57.61 | 55.72 | 57.68 | 74.38 | 20.11 | 54.63 |
| **D10** | 67.52 | 46.85 | 50.16 | 60.58 | 54.10 | 50.61 | 25.99 | 57.12 |
| **D11** | 41.34 | 57.61 | 58.63 | 57.68 | 38.09 | 49.59 | 0.60 | 46.40 |
| **E02** | 67.95 | 44.39 | 54.31 | 46.18 | 23.69 | 16.19 | 28.80 | 47.51 |
| **E03** | 62.19 | 35.33 | 87.18 | 48.22 | 41.75 | 56.40 | 20.53 | 52.32 |
| **E04** | 64.64 | 79.47 | 28.97 | 28.80 | 46.18 | 52.91 | 12.01 | 34.39 |
| **E05** | 78.20 | 48.12 | 59.64 | 30.76 | 11.33 | 45.58 | 70.63 | 33.79 |
| **E06** | 47.27 | 71.51 | 14.74 | 36.81 | 52.06 | 73.10 | 70.55 | 44.68 |
| **E07** | 76.08 | 57.44 | 65.07 | 31.70 | 15.17 | 31.01 | 22.58 | 31.56 |
| **E08** | 58.37 | 56.59 | 55.32 | 60.24 | 50.27 | 20.02 | 11.59 | 71.36 |
| **E09** | 34.40 | 61.51 | 51.34 | 44.05 | 55.72 | 28.46 | 26.92 | 53.17 |
| **E10** | 50.83 | 41.94 | 39.31 | 55.21 | 29.40 | 12.87 | 20.36 | 49.23 |
| **E11** | 54.98 | 61.17 | 33.04 | 51.12 | 4.52 | 68.50 | 48.31 | 56.17 |
| **F02** | 87.09 | 49.39 | 46.43 | 36.13 | 44.22 | 68.84 | 2.22 | 27.36 |
| **F03** | 55.58 | 41.26 | 63.71 | 61.52 | 39.45 | 69.95 | 27.86 | 34.22 |
| **F04** | 64.47 | 50.24 | 58.63 | 54.96 | 47.29 | 29.48 | 32.80 | 69.21 |
| **F05** | 30.42 | 65.49 | 58.88 | 47.20 | 62.28 | 2.56 | 37.40 | 54.97 |
| **F06** | 30.25 | 85.99 | 45.07 | 68.67 | 70.97 | 42.69 | 25.31 | 24.27 |
| **F07** | 52.27 | 57.10 | 71.93 | 44.73 | 74.47 | 56.75 | 27.01 | 26.59 |
| **F08** | 58.63 | 44.82 | 69.81 | 43.45 | 63.05 | -3.83 | 64.41 | 37.48 |
| **F09** | 48.72 | 55.58 | 54.39 | 82.39 | 49.25 | 50.36 | 33.48 | 65.95 |
| **F10** | 53.46 | 27.28 | 49.90 | 84.44 | 57.68 | 20.79 | 28.03 | 53.86 |
| **F11** | 67.52 | 38.04 | 10.59 | 38.60 | 26.58 | 37.32 | 2.73 | 61.84 |
| **G02** | 50.66 | 40.33 | 71.42 | 65.27 | 42.35 | 33.91 | 10.99 | 60.81 |
| **G03** | 53.29 | 47.70 | 60.07 | 36.64 | 54.62 | 19.94 | 24.45 | 34.99 |
| **G04** | 66.85 | 56.26 | 64.30 | 48.31 | 55.21 | 11.67 | 21.47 | 68.01 |
| **G05** | 64.56 | 73.11 | 65.49 | 22.66 | 44.82 | 16.44 | 39.19 | 55.57 |
| **G06** | 62.61 | 42.02 | 62.95 | 46.61 | 37.57 | 40.56 | 28.20 | 59.52 |
| **G07** | 64.64 | 64.22 | 66.00 | 28.88 | 56.15 | 28.29 | 69.95 | 47.68 |
| **G08** | 71.51 | 47.95 | 54.31 | 49.93 | 27.35 | 53.93 | 67.23 | 62.26 |
| **G09** | 57.02 | 43.38 | 61.08 | 34.17 | 23.26 | 42.26 | 68.59 | 49.83 |
| **G10** | 57.53 | 30.84 | 67.18 | 58.62 | 55.21 | 26.58 | 71.91 | 44.51 |
| **G11** | 38.38 | 37.96 | 74.64 | 54.96 | 44.39 | 20.53 | 71.23 | 34.56 |
| **H02** | 40.58 | 54.39 | 37.96 | 65.27 | 51.04 | 37.66 | 72.59 | 45.63 |
| **H03** | 48.72 | 58.03 | 46.34 | 42.01 | 27.95 | 34.25 | 54.44 | 40.82 |
| **H04** | 25.76 | 48.80 | 52.53 | 30.50 | 55.47 | 34.59 | 71.57 | 59.61 |
| **H05** | 28.97 | 68.03 | 46.17 | 27.35 | 59.73 | 14.57 | 66.71 | 40.91 |
| **H06** | 6.44 | 39.65 | 55.15 | 67.14 | 29.22 | 21.30 | 67.82 | 25.81 |
| **H07** | 21.69 | 75.74 | 64.56 | 30.84 | 25.22 | 30.67 | 70.63 | 48.80 |
| **H08** | 88.53 | 39.06 | 52.02 | 52.66 | 44.65 | 20.96 | 40.64 | 44.68 |
| **H09** | 39.57 | 69.39 | 64.05 | 31.95 | 33.74 | 64.24 | 71.49 | 54.63 |
| **H10** | 68.46 | 39.82 | 62.10 | 25.39 | 55.64 | 19.94 | 66.12 | 32.50 |
| **H11** | 64.13 | 41.60 | 23.38 | 38.77 | 45.16 | 77.79 | 65.10 | 18.18 |

*^a^* Negative control was added to the first and 12th columns of the screened 96-well microtiter plates, the wells exhibiting the top twenty inhibition rates were highlighted in red.

**Supplementary Table 5** YTHDC1 inhibition rate of the click-library plate 18~22 in first round screening.

| **Sub-library C2** Inhibition Rate (%) *^a^* | | | | | | |
| --- | --- | --- | --- | --- | --- | --- |
| **Plate** | **18** | **19** | **20** | **21** | **22** |  |
| **A02** | 16.55 | 18.61 | 67.32 | 50.36 | 89.99 |  |
| **A03** | 27.19 | 5.83 | 61.32 | 92.35 | 53.26 |  |
| **A04** | 35.08 | 46.57 | 66.47 | 69.59 | 46.55 |  |
| **A05** | 48.20 | 39.02 | 66.98 | 27.06 | 83.65 |  |
| **A06** | 13.12 | 11.84 | 45.54 | 62.79 | 94.98 |  |
| **A07** | 63.12 | 65.87 | 50.94 | 85.10 | 44.92 |  |
| **A08** | 38.42 | 16.64 | 64.92 | 90.99 | 73.49 |  |
| **A09** | 55.32 | 28.73 | 54.89 | 70.41 | 87.82 |  |
| **A10** | 55.92 | 25.90 | 51.97 | 71.49 | 59.07 |  |
| **A11** | 74.19 | 19.64 | 62.18 | 15.45 | 16.35 |  |
| **B02** | 47.86 | 53.43 | 61.66 | 49.27 | 98.25 |  |
| **B03** | 39.88 | 66.38 | 66.04 | 66.05 | 80.83 |  |
| **B04** | 57.98 | 57.46 | 59.86 | 95.71 | 84.10 |  |
| **B05** | 22.90 | 66.90 | 68.18 | 87.45 | 95.62 |  |
| **B06** | 75.39 | 37.39 | 74.01 | 70.41 | 87.55 |  |
| **B07** | 20.58 | 32.68 | 74.27 | 56.44 | 70.04 |  |
| **B08** | 35.42 | 54.46 | 76.76 | 98.34 | 49.27 |  |
| **B09** | 39.37 | 39.97 | 55.75 | 98.25 | 56.35 |  |
| **B10** | 26.33 | 18.87 | 82.68 | 85.28 | 95.89 |  |
| **B11** | 76.76 | 20.24 | 72.30 | 66.96 | 93.71 |  |
| **C02** | 23.76 | 28.47 | 68.27 | 96.34 | 85.01 |  |
| **C03** | 79.59 | 64.15 | 40.14 | 100.33 | 43.38 |  |
| **C04** | 64.92 | 61.66 | 69.13 | 85.37 | 80.11 |  |
| **C05** | 85.51 | 67.07 | 41.34 | 91.26 | 91.35 |  |
| **C06** | 68.78 | 7.55 | 64.84 | 91.99 | 92.53 |  |
| **C07** | 75.81 | 46.57 | 51.46 | 79.02 | 93.80 |  |
| **C08** | 52.57 | 40.57 | 63.55 | 83.83 | 36.12 |  |
| **C09** | 37.56 | 59.86 | 21.78 | 30.59 | 20.80 |  |
| **C10** | 57.72 | 50.60 | 77.02 | 49.27 | 25.15 |  |
| **C11** | 43.14 | 34.31 | 75.21 | 72.04 | 40.39 |  |
| **D02** | 55.15 | 24.36 | 67.58 | 66.60 | 11.00 |  |
| **D03** | 65.35 | 19.30 | 57.80 | 90.81 | 29.96 |  |
| **D04** | 44.08 | 24.70 | 63.55 | 89.45 | 43.20 |  |
| **D05** | 52.06 | 39.28 | 73.93 | 96.89 | 32.50 |  |
| **D06** | 31.48 | 25.81 | 62.26 | 85.37 | 24.70 |  |
| **D07** | 56.17 | 41.60 | 64.41 | 89.18 | 54.17 |  |
| **D08** | 74.53 | 59.86 | 56.26 | 91.99 | 35.76 |  |
| **D09** | 64.49 | 25.56 | 73.58 | 83.65 | 32.32 |  |
| **D10** | 55.49 | 57.29 | 43.83 | 76.66 | 30.32 |  |
| **D11** | 44.60 | 20.84 | 54.46 | 91.17 | 39.66 |  |
| **E02** | 31.39 | 34.05 | 81.39 | 89.18 | 39.21 |  |
| **E03** | 37.99 | 8.06 | 74.96 | 93.35 | 52.63 |  |
| **E04** | 51.03 | 35.25 | 61.23 | 96.61 | 27.51 |  |
| **E05** | 49.23 | 52.66 | 75.21 | 70.68 | 50.91 |  |
| **E06** | 46.83 | 14.49 | 71.44 | 38.03 | 43.38 |  |
| **E07** | 35.16 | 26.93 | 70.93 | 95.44 | 64.96 |  |
| **E08** | 53.77 | 23.16 | 78.90 | 80.11 | 32.59 |  |
| **E09** | 47.08 | 30.96 | 50.51 | 92.99 | 27.78 |  |
| **E10** | 43.91 | 23.93 | 79.07 | 75.21 | 20.71 |  |
| **E11** | 46.66 | 45.03 | 75.30 | 74.94 | 57.71 |  |
| **F02** | 35.08 | 46.74 | 66.04 | 91.17 | 31.77 |  |
| **F03** | 36.19 | 63.04 | 62.44 | 38.85 | 31.32 |  |
| **F04** | 2.74 | 40.39 | 63.89 | 97.88 | 15.54 |  |
| **F05** | 41.77 | 45.97 | 58.49 | 91.81 | 49.46 |  |
| **F06** | 51.89 | 3.77 | 52.32 | 85.10 | 37.03 |  |
| **F07** | 57.38 | 68.27 | 45.54 | 42.56 | 52.54 |  |
| **F08** | 44.00 | 27.36 | 57.80 | 27.87 | 35.49 |  |
| **F09** | 5.15 | 48.63 | 56.78 | 88.27 | 22.25 |  |
| **F10** | 41.42 | 67.75 | 53.17 | 87.82 | 28.23 |  |
| **F11** | 67.32 | 65.78 | 45.03 | 79.11 | 26.78 |  |
| **G02** | 65.87 | 62.26 | 64.41 | 80.74 | 37.58 |  |
| **G03** | 54.89 | 65.61 | 57.89 | 68.68 | 40.48 |  |
| **G04** | 65.35 | 67.15 | 69.90 | 95.25 | 44.29 |  |
| **G05** | 61.66 | 63.46 | 79.25 | 48.46 | 36.49 |  |
| **G06** | 32.25 | 47.94 | 80.10 | 89.45 | 30.41 |  |
| **G07** | 54.46 | 72.47 | 73.84 | 92.53 | 38.66 |  |
| **G08** | 63.72 | 68.35 | 72.21 | 84.10 | 16.81 |  |
| **G09** | 44.85 | 65.18 | 71.96 | 84.19 | 21.16 |  |
| **G10** | 58.58 | 63.89 | 68.78 | 92.17 | 18.35 |  |
| **G11** | 46.66 | 66.64 | 80.53 | 94.71 | 37.58 |  |
| **H02** | 45.20 | 72.81 | 58.06 | 32.68 | 50.27 |  |
| **H03** | 31.82 | 72.30 | 56.26 | 64.33 | 44.83 |  |
| **H04** | 55.66 | 69.04 | 59.61 | 78.39 | 35.94 |  |
| **H05** | 9.52 | 68.78 | 50.00 | 90.27 | 52.63 |  |
| **H06** | 21.61 | 29.42 | 48.28 | 87.00 | 23.52 |  |
| **H07** | 39.45 | 70.84 | 62.86 | 83.56 | 51.00 |  |
| **H08** | 32.50 | 66.30 | 73.24 | 90.36 | 25.51 |  |
| **H09** | 43.40 | 68.44 | 90.57 | 95.98 | 24.97 |  |
| **H10** | 23.76 | 61.84 | 53.86 | 73.31 | 30.32 |  |
| **H11** | 54.55 | 70.58 | 60.89 | 68.14 | 27.15 |  |

*^a^* Negative control was added to the first and 12th columns of the screened 96-well microtiter plates, the wells exhibiting the top twenty inhibition rates were highlighted in red.

**Supplementary Fig. 13 a** BLI analysis revealed that the dissociation constant (K_d_) between YTHDC1 and LC-YD03 is 102 nM. **b** BLI analysis revealed that the dissociation constant (K_d_) between YTHDC1 and compound **25** is 189 nM.

**Supplementary Fig. 14** Selectivity profiling of LC-YD03. **a** FP-based inhibition of YTH family proteins by LC-YD03 at 50 μM. **b** AlphaScreen-based inhibition of YTH family proteins by LC-YD03 at 10 μM. **c** Inhibitory activity of LC-YD03 against the METTL3/METTL14 complex. Data are shown as mean ± SD from three independent experiments.

**Supplementary Fig. 15 a** Inhibition rates of LC-YD03 against 41 kinases at 1000 nM. **b** Distribution of the tested kinases across the kinase phylogenetic tree. Green circles indicate the tested kinases. Note: PI3Kα and PI3Kδ could not be displayed in the kinase phylogenetic tree; their representative colors correspond to the branch colors shown in the legend.

**Supplementary Fig. 16** FP, and AlphaScreen analyses of YTHDC1 mutants. **a** FP assay windows of WT, N363A, N364A, and S378A YTHDC1, shown as ΔmP（n=3）. **b, c,** and **d**, FP inhibition curves of LC-YD03 against WT, N363A, and S378A YTHDC1（n=3）. **e** AlphaScreen signals of WT, N363A, N364A, and S378A YTHDC1 with the m⁶A RNA probe. Data are shown as mean ± SD（n=3）.

**Supplementary Fig. 17** Structural optimization and biological evaluation of LC-YD03 derivatives. **a** Effect of LC-YD03 on the growth of THP-1 cells. Cell viability was measured after 7 days of treatment（n=3）.**b** Effect of LC-YD03 on the growth of MOLM-13 cells. Cell viability was measured after 7 days of treatment（n=3）. **c** Intracellular exposure of LC-YD03 and Compound 25 in THP-1 cells（n=3）. **d** Structure optimization strategy of LC-YD03 and design of derivatives LC-YD07–LC-YD19. **e** YTHDC1 inhibitory activity and thermal stabilization effects of LC-YD02–LC-YD19 measured by AlphaScreen, TSA, CTG assays. Data are shown as mean ± SD (n = 3).

**Supplementary Fig. 18** TSA and AlphaScreen analyses of LC-YD derivatives against YTHDC1. **a** Effects of LC-YD16, LC-YD17, LC-YD18, and LC-YD19 on the thermal stability of YTHDC1 measured by TSA（n=3）. **b** AlphaScreen inhibition curves of LC-YD16, LC-YD17, LC-YD18 and LC-YD19 against the interaction between YTHDC1 and the m⁶A RNA probe. IC₅₀ values were determined using a four-parameter logistic equation. Data are shown as mean ± SD（n=3）.

**Supplementary Fig. 19** Selectivity profiling of LC-YD17. **a** FP-based inhibition of YTH family proteins by LC-YD17 at 50 μM. **b** AlphaScreen-based inhibition of YTH family proteins by LC-YD17 at 10 μM. **c** Inhibitory activity of LC-YD17 against the METTL3/METTL14 complex. Data are shown as mean ± SD from three independent experiments.

**Supplementary Table 6** Comparison of the advantages and application limitations of AS-TLC, AS-MS, DEL, and NMR

| Technology | Major advantages | Major application limitations |
| --- | --- | --- |
| AS-TLC | Low cost, low technical barrier, robust against interference, and combines activity screening with compound tracing. | Limited by detergents, covalent compounds, UV visualization, and the scale of the physical compound library. |
| AS-MS | Direct compound identification, suitable for complex mixtures, with relatively high throughput. | Susceptible to differences in ionization efficiency, dissociation of weak ligands, and competition within pooled mixtures. |
| DEL | Largest chemical space, highest throughput, and low sample consumption. | Limited by DNA chemical compatibility, synthesis quality, linker effects, and off-DNA resynthesis. |
| NMR | Excels at weak-binding/fragment screening and can provide rich binding information. | Relatively low sensitivity and throughput, with high requirements for protein quality, instrumentation, and professional expertise. |

**Supplementary Fig.20** Targeting USP7, 1,280 compounds were screened via AS-MS. **a-d** The AS-MS chromatograms of 160 screened compounds in group 1-4. The unique chromatographic peaks of candidate substances to be analyzed in the experimental groups, which are absent in the control group, are marked in red. **e-h** The AS-MS chromatograms of 160 screened compounds in group 5-8. **i** The mass spectrometry results of the substance corresponding to the red chromatographic peak in group1 which was identified as ALM4. **j** The mass spectrometry results of the substance corresponding to the red chromatographic peak in group 2, which was identified as GNE-6640.

**Supplementary Fig.21** Targeting USP7, 2,560 compounds were screened via AS-MS. **a-p** The AS-MS chromatograms of 160 screened compounds in each group. The unique chromatographic peaks of candidate substances to be analyzed in the experimental groups, which are absent in the control group, are marked in red. **q** The mass spectrometry results of the substance corresponding to the red chromatographic peak in group 6. **r** The mass spectrometry results of the substance corresponding to the red chromatographic peak in group 14.

**Supplementary Table 7 Compounds of similar exact molecular weight in screening library.**

| Sample ID | *m/z* | Compounds of similar exact molecular weight | Analysis |
| --- | --- | --- | --- |
| Group 6 | 485.05 | Not found | An isotope peak appears here. Note that the compound may contain chlorine. |
| Group 14 | 391.23 | 390.49 | / |
|  |  | 390.27 |  |

**Supplementary Fig.22** Validation of AS-MS screening results targeting USP7. **a** AS-MS chromatogram of the compound with a molecular weight of 390.49 and USP7. **b** AS-MS chromatogram of the compound with a molecular weight of 390.27 and USP7. **c** AS-MS chromatogram of the negative control group containing USP7 without any compound in the reaction system. **d** AS-MS chromatogram of the positive control group with ALM4 and USP7 included in the reaction system. The unique chromatographic peaks of candidate substances to be analyzed in the experimental groups, which are absent in the control group, are marked in red. **e** The mass spectrometry results of the substance corresponding to the red chromatographic peak in positive control, which was identified as ALM4.

**Supplementary Fig.23** The enzyme activity assay system combined with AS-TLC was used to screen 3,840 compounds targeting USP7. **a-c** In the enzyme activity assay stage, the fluorescence of rhodamine after TLC development of the samples. White triangle mark two experimental groups with significantly weakened rhodamine fluorescence, whose corresponding compound mixtures were subjected to further AS-TLC to identify hit compounds. **d** 50-8E was identified from the first compound mixture in **a** through AS-TLC as the positive compound ALM4. **e** 1-B07 was identified from the fifth compound mixture in **a** through AS-TLC as the positive compound GNE-6640.

**Supplementary Table 8** Comparison of protein consumption and time required between two screening strategies*^a^*.

| Method | USP7 dosage | Time consumed | NO. of hits |
| --- | --- | --- | --- |
| AS-MS | 120 nmol | 12 h | 2 |
| AS-TLC combined  with activity assay | 10.12 nmol | 8 h | 2 |

*^a^* The comparative data were derived from the AS-MS experiment screening 3,840 compounds presented in Supplementary Fig. 20-22, as well as the experiment of the AS-TLC combined with enzyme activity assay system screening 3,840 compounds shown in Supplementary Fig. 23.

**Supplementary Fig.24** AS-MS screening of 800 compounds was performed for targeting CDK2. **a-e** The AS-MS chromatograms of 160 screened compounds in each group targeting CDK2. **f** The mass spectrometry results of the substance corresponding to the red chromatographic peak in group 1, which was identified as Tagtociclib. **g** The mass spectrometry results of the substance corresponding to the red chromatographic peak in group 3, which was identified as AT7519. The 800 compounds used in this experiment were sourced from the same origin as those in Supplementary Fig. 2e.

**Supplementary Fig.25** AS-MS screening of 800 compounds was performed for targeting YTHDC1. **a-e** The AS-MS chromatograms of 160 screened compounds in group 1-5 targeting YTHDC1. Chromatographic peaks of the substances to be analyzed are marked in red. **f** The mass spectrometry results of the substance corresponding to the red chromatographic peak in group 5, which was identified as compound **25**. The 800 compounds used in this experiment share an identical source with group 2-6 compounds in Supplementary Fig. 9d.

**Supplementary Table 9** Comparison of compounds hit in AS-TLC combined with activity assay and AS-MS*^a^*.

| Compounds | IC_50_/K_d_ | AS-MS | AS-TLC combined  with activity assay |
| --- | --- | --- | --- |
| GNE-6640 | IC_50_ = 0.4 μM | hit | hit |
| ALM4 | IC_50_ = 1.5 nM | hit | hit |
| Tagtociclib | IC_50_ = 1.16 μM | hit | hit |
| AT7519 | IC_50_ = 47 nM | hit | hit |
| 164-A02 | K_d_ = 1.98 μM | miss | hit |
| compound **25** | IC_50_ = 177.6 nM | hit | hit |
| fragment YD | IC_50_ = 20.01 μM | miss | hit |

*^a^* The comparative data were derived from the AS-MS experiment in Supplementary Fig. 20 and Supplementary Fig. 24-25, as well as the experiment of the AS-TLC/ AS-TLC combined with activity assay system shown in Supplementary Fig. 2e, Supplementary Fig. 4, Fig. 2b-e and Supplementary Fig. 9c-i.

**Supplementary Fig.26 a** AS-TLC experimental group sample (left) containing membrane protein DHCR7 with mixed compounds and the protein-free compound-containing AS-TLC control group sample (right). **b** The impact of detergents in the system on final compound detection during AS-TLC experiments with membrane proteins. **c** Different spotting amounts of 10 mM covalent compound USP7/47 inhibitor all showed significant visibility under 254 nm UV light. **d** TLC detection of the AS-TLC experimental group sample (left) containing USP7 with inhibitor and the inhibitor standard sample (right).

**Supplementary Table 10** X-ray Diffraction Data Collection and Refinement Statistics

|  | **YTHDC1+fragment YD**  **（PDB: 9VS9）** | | | **YTHDC1+LC-YD01**  **（PDB: 9VRA）** | **YTHDC1+LC-YD03**  **（PDB: 9VSB）** |
| --- | --- | --- | --- | --- | --- |
| **Resolution range** | 38.6 - 1.824 (1.889 - 1.824) | | | 39.25 - 2.493 (2.582 - 2.493) | 27.32 - 1.804 (1.868 - 1.804) |
| **Space group** | P 1 2_1_ 1 | | | P 1 2_1_ 1 | P 1 2_1_ 1 |
| **Unit cell** | 39.857 103.277 41.862 90 104.425 90 | | | 40.166 103.443 41.522 90 102.288 90 | 39.448 103.341 41.452 90 105.587 90 |
| **Total reflections** | 184697 (18404) | | | 42674 (4183) | 129408 (13009) |
| **Unique reflections** | 29119 (2881) | | | 11589 (1168) | 28710 (2829) |
| **Multiplicity** | 6.3 (6.4) | | | 3.7 (3.6) | 4.5 (4.6) |
| **Completeness (%)** | 97.33 (97.14) | | | 99.59 (98.80) | 96.76 (96.95) |
| **Mean I/sigma(I)** | 15.72 (3.13) | | | 6.37 (2.59) | 10.13 (2.52) |
| **Wilson B-factor** | 21.27 | | | 12.18 | 18.39 |
| **R-merge** | 0.0898 (1.194) | | | 0.2716 (1.233) | 0.1112 (0.7052) |
| **Reflections used in refinement** | 28418 (2818) | | | 11563 (1154) | 28457 (2829) |
| **Reflections used for R-free** | 1989 (199) | | | 605 (74) | 1426 (139) |
| **R-work** | 0.2168 (0.2909) | | | 0.2586 (0.3123) | 0.2517 (0.3147) |
| **R-free** | 0.2532 (0.3303) | | | 0.2909 (0.3369) | 0.2785 (0.3461) |
| **No. atoms** | 2769 | | | 2681 | 2795 |
| **macromolecules** | 2612 | | | 2615 | 2638 |
| **ligands** | 29 | | | 35 | 66 |
| **solvent** | 128 | | | 31 | 91 |
| **RMS(bonds)** | | 0.008 | 0.060 | | 0.279 |
| **RMS(angles)** | | 0.99 | 1.01 | | 3.17 |
| **Ramachandran favored (%)** | | 98.76 | 99.07 | | 97.21 |
| **Ramachandran allowed (%)** | | 1.24 | 0.93 | | 2.79 |
| **Average B-factor** | | 27.72 | 16.21 | | 28.28 |
| **macromolecules** | | 27.67 | 16.10 | | 28.01 |
| **ligands** | | 30.58 | 22.53 | | 41.97 |
| **solvent** | | 28.02 | 18.25 | | 26.25 |

Statistics for the highest-resolution shell are shown in parentheses.
