# Supplementary material for: AS‐TLC‐Based High‐Throughput Screening Integrated with CuAAC Optimization Strategy Facilitates the Rapid Discovery of YTHDC1 Inhibitor
Source: Adv Sci (Weinh). 2026 Aug 27:e77386. Online ahead of print. doi: 10.1002/advs.77386 (PMC13521195; doi:10.1002/advs.77386)
Supplement: Supplementary file 1 [file ADVS-9999-e77386-s001.docx]

**Supporting Information**

**AS-TLC-Based High-Throughput Screening Integrated with CuAAC Optimization Strategy Facilitates the Rapid Discovery of YTHDC1 Inhibitor**

Mingchen Wang ^a, b, c, d, +^, Shuiping Fu ^d, e, +^, Ye Xu ^a, d, +^, Jia Gao ^f^, Jiwei Ren ^f^, Xu Yang ^c^, Hesong Xu ^a^, Ge Sun ^d^, Yanjie Li ^a, d^, Yanlin Wang ^f^, Lu Jin ^f^, Peijuan Chen ^g^, Ying Xu ^d^, Jianhao Li ^a, c^, Hua Lin ^g^, Chujiao Hu ^b^, Kaixian Chen ^d^, Huan Xiong ^f, *^, Cheng Luo ^a, b, d, e, h, *^, Shijie Chen ^c, d, *^

*^a^ School of Pharmaceutical Science and Technology, Hangzhou Institute for Advanced Study, University of Chinese Academy of Sciences, Hangzhou, 310024, China.*

*^b^ Precision Medicine Research Institute of Guizhou, and Guizhou Provincial Key Laboratory of Digestive System Diseases, The Affiliated Hospital of Guizhou Medical University, Guizhou Medical University, Guiyang, 550014, China.*

*^c^ Innovation Center for AI and Drug Discovery, School of Pharmacy, East China Normal University, Shanghai 200062, China.*

*^d^ State Key Laboratory of Drug Research, Shanghai Institute of Materia Medica, Chinese Academy of Sciences, Shanghai, 201203, China.*

*^e^ University of Chinese Academy of Sciences, Beijing 100049, China*

*^f^ Zhongshan Institute for Drug Discovery, Shanghai Institute of Materia Medica, Chinese Academy of Sciences, Zhongshan 528400, China.*

*^g^ Key Laboratory of Microbial Pathogenesis and Interventions of Fujian Province University, the Key Laboratory of Innate Immune Biology of Fujian Province, Biomedical Research Center of South China, College of Life Sciences, Fujian Normal University, Fuzhou 350117, China.*

*^h^ Tianfu Jincheng Laboratory, Chengdu, 610212, China.*

^+^ *These authors contributed equally to this work.*

*^*^ Corresponding author.*

*E-mail addresses:* [*xionghuan@zidd.ac.cn*](mailto:xionghuan@zidd.ac.cn) *(Huan Xiong); cluo@simm.ac.cn (Cheng Luo); sjchen@pharm.ecnu.edu.cn (Shijie Chen)*

**Table of Contents**

- **Material and Methods**

1. **Material Table**
2. **Library construction and product analysis by UPLC-MS**
3. **Chemistry**

General remarks

Synthesis of CPJ-4-1

Synthesis of compound YD-1/2/3 and LC-YD01

Synthesis of compound FP-YD

Synthesis of LC-YD01 derived triazole compounds

Synthesis of compound LC-YD07

Synthesis of compound LC-YD08/09

Synthesis of compound LC-YD10

Synthesis of compound LC-YD15

Synthesis of compound LC-YD14

Synthesis of compound LC-YD16/17/18/19

- **Spectrums of compound characterization**

**Material Table**

| MATERIAL | SOURCE | IDENTIFIER |
| --- | --- | --- |
| [TLC silica gel plate](https://www.jkchemical.com/product/2522673) | J&K Scientific | 937930 |
| plasmid | Beijing Tsingke Biotech | N/A |
| spin desalting plate | Thermo Scientific | 89807 |
| ub-rho110 | KS-V Peptide | N/A |
| biacore series sensor chip cm5 | Cytiva | 29149604 |
| GSTrap ff column | GE Healthcare | 10278066 |
| Hitrap heparin hp column | Cytiva | 17040703 |
| superdex 75 increase 10/300 gl | Cytiva | 29148721 |
| BL21(DE3) | WEIDI | EC1002L |
| petroleum ether | Sinopharm Chemical Reagent | 80098618 |
| ethyl acetate | Sinopharm Chemical Reagent | 10009418 |
| methanol | Sinopharm Chemical Reagent | 80080418 |
| dichloromethane | Sinopharm Chemical Reagent | 80047318 |
| acetonitrile | Sinopharm Chemical Reagent | 40064160 |
| dimethyl sulfoxide | Sigma aldrich | D8418 |
| 0.5 m edta, ph 8.0 | Sangon Biotech | B540625-0500 |
| imidazole | Sinopharm Chemical Reagent | 30104916 |
| sodium chloride | Sangon Biotech | A501218-0005 |
| dl-dithiothreitol | Beyotime | ST043 |
| HEPES, sodium salt | Sangon Biotech | A600265-0250 |
| 2-mercaptoethanol | Sinopharm Chemical Reagent | 80076918 |
| magnesium chloride hexahydrate | Sigma Aldrich | M2670 |
| isopropyl β-d-thiogalactopyranoside | Sangon Biotech | A600168-0100 |
| glycerol | Sangon Biotech | A501745-0500 |
| CHAPS | Sinopharm Chemical Reagent | xw02000660 |
| GSH | VETEC | V900456 |
| tris | Sangon Biotech | A501492-0500 |
| tween-20 | Sangon Biotech | A600560-0500 |
| poly-l-lysine | Sangon Biotech | A600751-0025 |
| agar | Sangon Biotech | A505255-0250 |
| yeast extract | OXID | LP0021B |
| tryptone | OXOID | LP0042B |
| kanamycin sulfate | Sangon Biotech | A506636-0100 |
| ampicillin, sodium salt | Diamond | A100339-0025 |
| phosphate buffer saline | MeilunBio | MA0015 |
| s-(5‘-adenosyl)-l-methionine chloride | Sigma | A7007 |
| SYPRO Orange dye | Invitrogen | S6650 |
| bovine serum albumin | Sangon Biotech | V900933 |
| Biotin-GAACCGGm^6^ACUGUCUUA) | GenScript | N/A |
| 6-FAM-GAACCGGm^6^ACUGUCUUA | GenScript | N/A |
| EnVision multilabel reader | PerkinElmer | EnVision® 2105 |

**Library construction and product analysis by UPLC-MS**

- **Preparation of the amine library in 96-deep-well plates.**

Our collection of in-house amines building blocks, totaling 1636, were dissolved in DMSO to prepare 100 mM stock solutions and distributed in 22 96-deep-well plates (2 mL/well) and stored at -80 °C. The first and 12th columns of 96-deep-well plates were left empty as positive control.

- **Procedure for the preparation of the azide library in 96-deep-well plates.**

Regents:

• FSO_2_N_3_ solution: The concentration of FSO_2_N_3_ is approximately 200 mM. It was synthesized following the reported literature.

• Aqueous KHCO_3_ solution: 3.0 M in water, made by adding water to dissolve KHCO_3_ (30 g, 0.30 mol) until a total volume of 100 mL.

• Sodium ascorbate/Na_2_HPO_4_/citric acid buffer: an aqueous acidic buffer (pH ca. 5), was made with sodium ascorbate (2.48g, 12.5 mmol), Na_2_HPO_4_ (7.0 g, 49.3 mmol), citric acid (4.87 g, 25.4 mmol), and water (to make a total volume of 100 mL).

• Aqueous CuSO_4_/THPTA solution: 5 mM concentration for both CuSO_4_ and THPTA; made by dissolving CuSO_4_ (16.0 mg, 100 µmol) and THPTA (43.5 mg, 100 µmol) in water (20 mL).

Procedure:

From the amine library in 96-deep-well plates (2 mL/well)**:**

- Transferred amine solutions (100 mM in DMSO, 20 μL per well, 2 μmol amine) to new 22 96-deep-well plates by pipetting workstation
- Added aqueous KHCO_3_ (3.0 M, 26.7 μL per well, 80 μmol) to each plate
- Added FSO_2_N_3_ solution (200 mM in DMSO/MTBE, 1:1 v/v, 100 μL per well, 20 μmol) to each plate
- Adjusted total volume to 400 μL with DMSO (73 μL per well)

The sealed plates were incubated with shaking (1000 rpm) at 37 °C for 2 h, then stored at -80°C for long-term preservation.

- **Procedure for the preparation of compound library YD in microplates.**

Procedure:

Azide solutions (50 mM in DMSO, 10 μL/well, 0.5 µmol) were transferred to 96-well microplates by pipetting workstation. To each well of the microplates was added the sodium ascorbate/Na_2_HPO_4_/citric acid buffer (15 µL), the microplates were then sealed and incubated at 37 ℃ for 15 min. To each well was added the solution of **LC-YD01** (25 mM in DMSO, 20 μL, 0.5 μmol), followed by the aqueous CuSO_4_/ THPTA solution (5 mM CuSO_4_/5 mM THPTA in H₂O, 5 µL). The plates were sealed and swirled at 1000 rpm and 40 ℃ overnight.

| Reagents | Concentration | Volume | Final Concentration |
| --- | --- | --- | --- |
| LC-YD01 | 25 mM in DMSO | 20 µL | 50 mM |
| Azide | 50 mM in DMSO/MTBE | 10 µL | 50 mM |
| THPTA/CuSO_4_ | 5 mM in Water | 5 µL | 0.25 mM |
| Buffer | / | 15 µL | / |
| Total Volume | 50 µL/well | | |

The detailed conditions for the construction of compound library YD：

The progression of the reaction was monitored using ultra-performance liquid chromatography-mass spectrometry (UPLC-MS) to confirm the formation of desired compounds. From the compound library, totaling 1636, 498 crude samples were analyzed, and their conversion efficiencies were quantified as percentages (**Supplementary Table 2**), and 20 representative compounds (**P1-E7, P2-C3, P3-A2, P4-F4, P6-B9, P7-F2, P9-H9, P10-A3, P11-D3, P12-A4, P13-B3, P13-D3, P14-B9, P14-F9, P15-A5, P15-H5, P16-D6, P16-F6, P17-B7, P20-A2.**) were selected for detailed profiling (**Supplementary Fig.11**).

**Chemistry**

**General remarks**

All reagents and solvents were purchased from commercial suppliers and used as received unless other noted. NMR spectra were recorded at ambient temperature using standard pulse methods on Bruker Avance Neo Ascend at 500 MHz or 600 MHz. ^1^H (500 MHz), ^13^C (151/126 MHz), ^19^F (471 MHz). The chemical shifts (*δ*) are expressed in parts per million (ppm) with reference of residual ^1^H or ^13^C of deuterated solvents: DMSO-*d*_6_ (2.50, 39.52). Coupling constants (*J*) are quoted to the nearest 0.1 Hz, and multiplicities are given by the following abbreviations and combinations thereof: s (singlet), d (doublet), t (triplet), q (quartet), m (multiplet), brs (broad singlet). Purification was performed by flash chromatography using a Biotage Isolera One system equipped with dual-wavelength UV detection (254 and 280 nm). Normal-phase and reversed-phase separation carried out on a Biotage Isolera One device. The purity of all final compounds was confirmed by HPLC with an Agilent 1260 spectrometer. HPLC analysis conditions: EC-C18, 2.7 μm, 4.6 mm × 100 mm. Method: YTH 5 to 95 ACN-15min: flow rate, 0.5 mL/min; eluent A, 0.1% formic acid in water; eluent B, acetonitrile; gradient conditions were initially 5% B, increasing linearly to 95% B over 10 min, decreasing linearly to 5% B over 1 min, remaining at 5% B for 4 min; YTH 5 to 95 ACN-30min: 0.5 mL/min; eluent C, 0.1% formic acid in water; eluent D, acetonitrile; gradient conditions were initially 5% D, increasing linearly to 95% D over 25 min, decreasing linearly to 5% D over 1 min, remaining at 5% D for 4 min. All compounds are >95% pure by HPLC analysis. Mass spectra were assessed on a Waters UPLC-MS spectrometer. The analytical method was as follows: flow rate, 0.3 mL/min; eluent A, 0.1% formic acid in water; eluent B, acetonitrile; gradient conditions were initially 5% B, increasing linearly to 95% B over 4 min, remaining at 95% B for 1 min, decreasing linearly to 5% B over 0.1 min, remaining at 5% B for 0.9 min. High-resolution mass spectroscopy (HRMS) data were recorded on a Thermo Scientific Exactive Plus spectrometer with H-ESI II probe for Ion Max Source.

**Supplementary Scheme 1.** Synthesis of compound CPJ-4-1

**Step 1**: To a solution of compound **1** (10.0 g, 43.1 mmol, 1 equiv.) in DCM (100 mL) was added cyclopentylamine (5.1 g, 51.7 mmol, 1.2 equiv.) and Et_3_N (12 mL, 46.2 mmol, 2 equiv.). The mixture was stirred at room temperature for 3 h and monitored by LC-MS. The mixture was extracted with DCM (3 × 150 mL), washed with brine (1 × 200 mL). The organic layers were combined and dried over Na_2_SO_4_ and the solvent was evaporated under reduced pressure. The crude product was purified by flash column chromatography (petroleum ether/ethyl acetate = 10/1, v/v) to afford intermediate **2** (10 g, 83%) as a yellow solid powder. UPLC-MS **(**ESI**)**: *m/z* 282.13 [M + H]^+^.

**Step 2**: A 500 mL single-necked flask equipped with a magnetic stir bar was charged with a mixture of intermediate **2** (10 g, 35.6 mmol, 1 equiv.) in 200 mL of dry THF. The mixture was cooled to 0 °C, followed by the dropwise addition of LiAlH_4_ (2.5 M in THF, 42.7 mL, 106.8 mmol, 3.0 equiv.). The mixture was stirred at room temperature for 2 h and monitored by LCMS. The mixture was cooled to 0 °C and stirred for 15 min, and then slowly added water by the dropwise addition until the reaction eases, added water (20 mL) and then added NaOH solution (1 mM, 100 mL). The mixture was stirred at room temperature for 1 h. Afterwards, the organic phase was collected and the solvent was evaporated under reduced pressure to obtain intermediate **3** (8g, 94%) which was used directly in the next step without purification. UPLC-MS **(**ESI**)**: *m/z* 240.08 [M + H]^+^.

**Step 3**: To a solution of intermediate **3** (8 g, 33.5 mmol, 1 equiv.) in DCM (100 mL) was added MnO_2_ (23.3 g, 268 mmol, 8 equiv.). The mixture was stirred at room temperature overnight. Then, the MnO_2_ in the mixture was filtered out and additional MnO_2_ (23.3 g, 268 mmol, 8 equiv.) was added and the mixture was stirred at room temperature for another 4 h. The reaction mixture was filtered to obtain an organic phase and the solvent was evaporated under reduced pressure to obtain intermediate **4** (7.5 g, 94%) which was used directly in the next step without purification. UPLC-MS **(**ESI**)**: *m/z* 238.05 [M + H]^+^.

**Step 4**: To a solution of intermediate **4** (7.5 g, 31.6 mmol, 1 equiv.) in acetic acid (60 mL) was added cyanoacetic acid (5.38 g, 63.2 mmol, 2 equiv.) and benzylamine (0.35 mL, 3.1 mmol, 0.1 equiv.). The mixture was stirred at 100 °C overnight and monitored by LCMS. The crude product was purified by flash column chromatography (DCM/MeOH = 100/5, v/v) to afford intermediate **5** (6.8 g, 75%) as a yellow solid powder. UPLC-MS **(**ESI**)**: *m/z* 286.85 [M + H]^+^.

**Step 5**: Intermediate **5** (6.8 g, 23.8 mmol, 1 equiv.) and 3-chloroperoxybenzoic acid (9.8 g, 57.2 mmol, 2.4 equiv.) were dissolved in DCM (50 mL) and the mixture was stirred at room temperature overnight and monitored by LCMS. The reaction mixture was extracted with DCM (3 × 80 mL), washed with brine (1 × 100 mL), and dried over anhydrous Na_2_SO_4_, concentrated *in vacuo* to afford intermediate **6** (6.5 g, 85%) as a yellow solid powder which was used for the next step without further purification. UPLC-MS **(**ESI**)**: *m/z* 302.98 [M + H]^+^.

**Step 6**: A reaction mixture containing intermediate **6** (6.5 g, 20.4 mmol, 1 equiv.), 4-amino-1-Boc-piperidine (6.12 g, 30.6 mmol, 1.5 equiv.), DIPEA (7.1mL, 40.8 mmol, 2 equiv.) and DCM (120 mL) was stirred at room temperature overnight and monitored by LC-MS. The mixture was extracted with DCM (3 × 150 mL), washed with brine (1 × 200 mL). The organic layers were combined and dried over Na_2_SO_4_ and the solvents were evaporated under reduced pressure. The crude product was purified by flash column chromatography (DCM/MeOH = 100/5, v/v) to afford intermediate **7** (7 g, 78%) as a yellow solid powder. UPLC-MS **(**ESI**)**: *m/z* 438.94 [M + H]^+^.

**Step 7**: To a solution of intermediate **7** (7 g, 15.9 mmol, 1 equiv.) in DCM (100 mL) was added trifluoroacetic acid (20 mL). The mixture was stirred at 0 °C for 3 h and monitored by LCMS. Then the mixture was evaporated under reduced pressure to obtain intermediate **8** (5 g, 93%) which was used directly in the next step without purification. UPLC-MS **(**ESI**)**: *m/z* 339.23 [M + H]^+^.

**Step 8**: A reaction mixture containing intermediate **8** (5.0 g, 14.8 mmol, 1 equiv.), Boc-5-aminopentanoic acid (6.4 g, 29.6 mmol, 2 equiv.), HATU (8.4 g, 22.2 mmol, 1.5 equiv.), DIPEA (7.7mL, 44.4 mmol, 3 equiv.) and DMF (80 mL) was stirred at room temperature for 5 h and monitored by LCMS. The mixture was extracted with EtOAc (3×100 mL), washed with brine (1×120 mL), and the combined organic layers were dried over anhydrous Na_2_SO_4_, concentrated *in vacuo.* The crude product was purified by flash column chromatography (DCM/MeOH = 10/1, v/v) to afford intermediate **9** (6 g, 75%) as a solid powder. UPLC-MS **(**ESI**)**: *m/z* 537.97 [M + H]^+^.

**Step 9**: Trifluoroacetic acid (20 mL) was added to a solution of intermediate **9** (6 g, 11.2 mmol, 1 equiv.) in DCM (100 mL). The mixture was cooled to 0 - 5 °C and stirred continuously on a magnetic stirrer for 30 min and monitored by LC-MS. Then the mixture was evaporated under reduced pressure to obtain intermediate **10** (4.5 g, 92%) which was used directly in the next step without purification. UPLC-MS **(**ESI**)**: *m/z* 438.29 [M + H]^+^.

**Step 10**: To a solution of intermediate **10** (50 mg, 0.11 mmol, 1 equiv.) in DMF (1.5 mL) was added fluorescein isothiocyanate isomer I (49 mg, 0.13 mmol, 1.1 equiv.) and K_2_CO_3_ (30.4 mg, 0.22 mmol, 2 equiv.). The mixture was stirred at room temperature overnight and monitored by LCMS. The crude product was purified by semi-preparative chromatographic column to afford compound **11** (mixture of rotamers) as a yellow solid powder (55 mg, 61%). HRMS (ESI) *m/z* calcd for C_44_H_43_N_8_O_7_S [M + H]^+^: 827.2970, found: 827.2966. ^1^H NMR (600 MHz, DMSO-*d*_6_) *δ* 9.88 (s, 1H), 8.73 (s, 1H), 8.67 (s, 1H), 8.55 (d, *J* = 7.6 Hz, 2H), 8.47 (s, 1H), 8.09 (s, 2H), 7.17 (d, *J* = 8.2 Hz, 1H), 6.60 (d, *J* = 8.7 Hz, 4H), 5.75 (q, *J* = 9.0 Hz, 1H), 4.02 (d, *J* = 8.9 Hz, 1H), 3.96 - 3.89 (m, 2H), 3.54 (s, 4H), 2.76 (s, 1H), 2.29 (s, 2H), 1.99 - 1.84 (m, 8H), 1.61 - 1.54 (m, 8H).

**Supplementary Scheme 2.** Synthesis of compound YD-1/2/3 and LC-YD01

**Step 1.** To a solution of 2,6-dichloropurine (1.0 equiv.) in DMF (0.5 M) were added K_2_CO_3_ (2.0 equiv.) and alkyl halide (1.5 equiv.). The reaction mixture was stirred at room temperature overnight until complete consumption of the starting material (monitored by TLC). The reaction mixture was extracted with ethyl acetate (× 3). The combined organic layers were washed with brine, dried over anhydrous Na_2_SO_4_, filtered, and concentrated under reduced pressure. The crude was purified by flash column chromatography on silica gel using a mixture of petroleum ether and ethyl acetate to afford the product.

**Step 2**. A suspension of 9-alkyl-2,6-dichloropurine (1.0 equiv.) in MeOH (0.5 M) was treated with methylamine (33 wt% in methanol, 3.0 equiv.). The mixture was stirred at room temperature for 2 h. After completion, the reaction mixture was concentrated to afford crude product. The crude was purified by flash column chromatography on silica gel using a mixture of petroleum ether and ethyl acetate to afford the product.

**2-Chloro-*N*,9-dimethyl-9*H*-purin-6-amine (YD-1).**

The **step 1** was used 2,6-dichloropurine (0.20 g, 1.06 mmol, 1.0 equiv.) and iodomethane (99 µL, 1.59 mmol, 1.5 equiv.) as starting materials, and the desired compound was obtained as a white solid (178 mg, 83% yield). UPLC-MS (ESI): *m/z* 202.83 [M + H]^+^.

The **step 2** was followed and the desired compound was obtained as a white solid (78 mg, 45% yield). ^1^H NMR (500 MHz, DMSO-*d*_6_) *δ* 8.14 (brs, 1H), 8.07 (s, 1H), 3.68 (s, 3H), 2.92 (d, *J* = 4.7 Hz, 3H). ^13^C NMR (126 MHz, DMSO-*d*_6_) *δ* 155.46, 153.17, 149.82, 141.66, 118.16, 29.57, 27.15. Purity: 99.9%. HRMS (ESI) *m/z* calcd for C_7_H_9_ClN_5_ [M + H]^+^: 198.0541, found: 198.0540.

**2-Chloro-9-ethyl-*N*-methyl-9*H*-purin-6-amine (YD-2).**

The **step 1** was used 2,6-dichloropurine (0.20 g, 1.06 mmol, 1.0 equiv.) and bromoethane (112 µL, 1.59 mmol, 1.5 equiv.) as starting materials, and the desired compound was obtained as a white solid (90 mg, 39% yield). UPLC-MS (ESI): *m/z* 216.92 [M + H]^+^.

The **step 2** was followed and the desired compound was obtained as a white solid (81 mg, 90% yield). ^1^H NMR (500 MHz, DMSO-*d*_6_) *δ* 8.17 (brs, 1H), 8.16 (s, 1H), 4.12 (q, *J* = 7.3 Hz, 2H), 2.91 (d, *J* = 4.7 Hz, 3H), 1.37 (t, *J* = 7.3 Hz, 3H). ^13^C NMR (126 MHz, DMSO-*d*_6_) *δ* 155.51, 153.10, 149.29, 140.71, 118.36, 38.28, 27.15, 15.19. Purity: 96.9%. HRMS (ESI) *m/z* calcd for C_8_H_11_ClN_5_ [M + H]^+^: 212.0697, found: 212.0696.

**2-Chloro-*N*-methyl-9-propyl-9*H*-purin-6-amine (YD-3).**

The **step 1** was used 2,6-dichloropurine (0.2 g, 1.06 mmol, 1.0 equiv.) and 1-bromopropane (144 µL, 1.59 mmol, 1.5 equiv.) as starting materials, and the desired compound was obtained as a white solid (177 mg, 72% yield). UPLC-MS (ESI): *m/z* 230.89 [M + H]^+^.

The **step 2** was followed and the desired compound was obtained as a white solid (156 mg, 79% yield). ^1^H NMR (500 MHz, DMSO-*d*_6_) *δ* 8.16 (brs, 1H), 8.13 (s, 1H), 4.05 (t, *J* = 7.1 Hz, 2H), 2.92 (d, *J* = 4.7 Hz, 3H), 1.78 (tq, *J* = 7.3, 7.1 Hz, 2H), 0.82 (t, *J* = 7.3 Hz, 3H). ^13^C NMR (126 MHz, DMSO-*d*_6_) *δ* 155.52, 153.15, 149.52, 141.18, 118.30, 44.70, 27.16, 22.68, 10.84. Purity: 99.9%. HRMS (ESI) *m/z* calcd for C_9_H_13_ClN_5_ [M + H]^+^: 226.0854, found: 226.0851.

**2-Chloro-*N*-methyl-9-(prop-2-yn-1-yl)-9*H*-purin-6-amine (LC-YD01).**

The **step 1** was used 2,6-dichloropurine (1.89 g, 10 mmol, 1.0 equiv.) and 3-bromoprop-1-yne (1.20 mL, 15 mmol, 1.5 equiv.) as starting materials, and the desired compound was obtained as a white solid (964 mg, 42% yield). ^1^H NMR (500 MHz, DMSO-*d*_6_) *δ* 8.77 (s, 1H), 5.19 (d, *J* = 2.6 Hz, 2H), 3.58 (t, *J* = 2.6 Hz, 1H). UPLC-MS (ESI): *m/z* 226.90 [M + H]^+^.

The **step 2** was followed and the desired compound was obtained as a white solid (972 mg, 99% yield). ^1^H NMR (500 MHz, DMSO-*d*_6_) *δ* 8.25 (brs, 1H), 8.19 (s, 1H), 5.01 (d, *J* = 2.6 Hz, 2H), 3.49 (t, *J* = 2.6 Hz, 1H), 2.92 (d, *J* = 4.7 Hz, 3H). ^13^C NMR (126 MHz, DMSO-*d*_6_) *δ* 155.52, 153.47, 149.04, 140.44, 118.16, 77.92, 76.23, 32.58, 27.19. Purity: 96.3%. HRMS (ESI) *m/z* calcd for C_9_H_9_ClN_5_ [M + H]^+^: 222.0541, found: 222.0540.

**Supplementary Scheme 3.** Synthesis of compound FP-YD.

**4-((2,6-Dichloro-9*H*-purin-9-yl) methyl)benzoic acid (12).**

To a solution of 2,6-dichloropurine (**11**) (378 mg, 2.0 mmol, 1.0 equiv.) in DMF (3 mL) were added K_2_CO_3_ (553 mg, 4.0 mmol, 2.0 equiv.) and 4-(bromomethyl) benzoic acid (430 mg, 2.0 mmol, 1.0 equiv.). The reaction mixture was stirred at room temperature overnight until completed consumption of the starting material (monitored by TLC). The reaction mixture was extracted with ethyl acetate (× 3) and 1 M hydrochloric acid solution. The combined organic layers were washed with brine, dried over anhydrous Na_2_SO_4_, filtered, and concentrated under reduced pressure. The residue was purified by C18 reversed-phase chromatography to give the title compound as a white solid (202 mg, 31% yield). ^1^H NMR (500 MHz, DMSO-*d*_6_) *δ* 12.99 (s, 1H), 8.86 (s, 1H), 7.94 - 7.89 (m, 2H), 7.43 - 7.39 (m, 2H), 5.59 (s, 2H). LC-MS (ESI): *m/z* 323.0 [M + H]^+^.

**4-((2-Chloro-6-(methylamino)-9*H*-purin-9-yl)methyl) benzoic acid (13)**.

A suspension of compound **12** (100 mg) in EtOH (2 mL) and 33% MeNH_2_ in EtOH (2 mL) was stirred at room temperature for 2 h. After the completed consumption of the starting material, the mixture was concentrated *in vacuo*. The crude was purified by flash column chromatography to afford the compound **13** as a white solid (92 mg, 94% yield). ^1^H NMR (500 MHz, DMSO-*d*_6_) *δ* 8.26 (s, 2H), 7.91 (d, *J* = 8.2 Hz, 2H), 7.33 (d, *J* = 8.2 Hz, 2H), 5.43 (s, 2H), 2.92 (d, *J* = 4.6 Hz, 3H). LC-MS (ESI): *m/z* 318.0 [M + H]^+^.

***N-*(6-aminohexyl)-4-((2-chloro-6-(methylamino)-*9H*-purin-9-yl)methyl) benzamide (15).**

A mixture of compound **13** (84 mg, 0.26 mmol, 1.0 equiv.), HATU (111 mg, 0.29 mmol, 1.2 equiv.), DIPEA (91 µL, 0.52 mmol, 2.0 equiv.), and *tert*-butyl (6-aminohexyl) carbamate (56 mg, 0.26 mmol, 1.0 equiv.) in DMF (5 mL) was stirred at room temperature until completion (monitored by TLC). The reaction mixture was diluted with water (15 mL) and extracted with EtOAc (10 mL × 3). The combined organic layers were washed with brine (20 mL), dried over anhydrous Na_2_SO_4_, and concentrated *in vacuo* to afford the crude compound **14** as a white solid (107 mg, 80% yield). The crude was used in the next step without further purification.

Compound **14** (107 mg) was dissolved in DCM (3 mL) and treated with 4 M HCl in ethyl acetate (1 mL). The reaction mixture was stirred at room temperature until completion (monitored by TLC). The solution was concentrated *in vacuo* to afford compound **15** as an off-white solid (80 mg). ^1^H NMR (500 MHz, DMSO-*d*_6_) *δ* 8.43 (s, 1H), 8.26 (s, 2H), 7.82 - 7.76 (m, 2H), 7.32 (d, *J* = 8.1 Hz, 2H), 5.40 (s, 2H), 3.64 (s, 4H), 3.22 (q, *J* = 6.6 Hz, 2H), 2.92 (s, 3H), 1.49 (t, *J* = 7.1 Hz, 2H), 1.39 - 1.32 (m, 2H), 1.31 - 1.23 (m, 4H). LC-MS (ESI): *m/z* 416.16 [M + H]^+^.

**4-((2-Chloro-6-(methylamino)-9*H*-purin-9-yl)methyl)-*N*-(6-(3-(3',6'-dihydroxy-3-oxo-3*H*-spiro[isobenzofuran-1,9'-xanthen]-5-yl)thioureido)hexyl) benzamide (FP-YD).**

Under argon atmosphere, a solution of compound **15** (58 mg, 0.14 mmol, 1.0 equiv.) in anhydrous DMF (2 mL) was treated successively with FITC (55 mg, 0.14 mmol, 1.0 equiv.) and anhydrous DIPEA (73 μL, 0.42 mmol, 3.0 equiv.). The reaction mixture was stirred in the dark at room temperature for 6 h, then quenched by addition of ice-cold water (10 mL). The crude was purified by C18 reversed-phase chromatography to afford **FP-YD** as an orange solid (48 mg, 43% yield). ^1^H NMR (500 MHz, DMSO-*d*_6_) *δ* 10.12 (s, 2H), 9.84 (s, 1H), 8.42 (t, *J* = 5.7 Hz, 1H), 8.32-8.16 (m, 3H), 8.10-8.02 (m, 1H), 7.79 (d, *J* = 7.8 Hz, 2H), 7.76-7.66 (m, 1H), 7.32 (d, *J* = 7.8 Hz, 2H), 7.17 (d, *J* = 8.3 Hz, 1H), 6.73 - 6.50 (m, 6H), 5.40 (s, 2H), 3.50 (s, 2H), 3.24 (q, *J* = 6.8 Hz, 2H), 2.92 (d, *J* = 4.2 Hz, 3H), 1.65 - 1.45 (m, 4H), 1.34 (s, 4H). ^13^C NMR (151 MHz, DMSO-*d*_6_) *δ* 180.45, 168.68, 165.89, 163.18, 159.54, 155.64, 153.54, 151.97, 149.54, 147.18, 141.28, 139.60, 134.32, 129.61, 129.13, 127.68, 127.23, 126.59, 124.12, 118.37, 116.51, 112.67, 109.82, 102.34, 83.12, 46.10, 43.90, 29.16, 28.40, 27.28, 26.32, 26.28. Purity: 99.93%. HRMS (ESI) *m/z* calcd for C_41_H_38_ClN_8_O_6_S [M + H]^+^: 805.2318, found: 805.2315.

***General procedure for the synthesis of LC-YD01 derived triazole compounds***

To a solution of corresponding primary amine (0.34 mmol, 1.5 equiv.) in DMSO (3.0 mL) was added FSO_2_N_3_ (330 mM in MTBE, 1.0 mL, prepared following the previous literature ¹) and KHCO_3_ (3.0 M in water, 1.0 mL). The reaction mixture was stirred for 2 h at room temperature. Then, sodium ascorbate/Na_2_HPO_4_/citric acid buffer (1 mL, prepared following the previous literature ¹) was added to the reaction mixture and stirred for another 15 min. To this stirred mixture was added LC-YD01 (50 mg, 0.22 mmol, 1.0 equiv.) followed by a solution of CuSO_4_•5H_2_O and THPTA (1:1 equiv., 5 mM in H₂O; 1.0 mL). The resulting mixture was stirred at 40 °C for 12 - 24 h. The crude product was purified by C18 reversed-phase chromatography (Biotage Sfär C18, 12 g, 30 µm; flow rate 20 mL/min) to afford the corresponding triazole product.

**2-(4-((2-Chloro-6-(methylamino)-9*H*-purin-9-yl)methyl)-1*H*-1,2,3-triazol-1-yl)-4-methylphenol (P2-C11).** White powder. 46% yield. ^1^H NMR (500 MHz, DMSO-*d*_6_) *δ* 10.30 (s, 1H), 8.51 (s, 1H), 8.25 (s, 1H), 8.23 (brs, 1H), 7.40 (dd, *J* = 2.1, 0.8 Hz, 1H), 7.13 (ddd, *J* = 8.3, 2.1, 0.8 Hz, 1H), 6.98 (d, *J* = 8.3 Hz, 1H), 5.51 (s, 2H), 2.92 (d, *J* = 4.4 Hz, 3H), 2.25 (s, 3H). ^13^C NMR (151 MHz, DMSO-*d*_6_) *δ* 155.55, 153.38, 149.38, 147.11, 141.91, 141.07, 130.61, 128.54, 125.17, 125.13, 123.96, 118.21, 116.92, 38.12, 27.19, 19.83. Purity: 99.9%. HRMS (ESI) *m/z* calcd for C_16_H_16_ClN_8_O [M + H]^+^: 371.1130, found: 371.1129.

**(*R*)-2-((*tert*-butoxycarbonyl)amino)-3-(4-(4-((2-chloro-6-(methylamino)-9*H*-purin-9-yl)methyl)-1*H*-1,2,3-triazol-1-yl)phenyl)propanoic acid (P3-E2).** White powder. 84% yield. ^1^H NMR (500 MHz, DMSO-*d*_6_) *δ* 12.63 (brs, 1H), 8.74 (s, 1H), 8.24 (s, 1H), 8.22 (brs, 1H), 7.78 (d, *J* = 8.3 Hz, 2H), 7.44 (d, *J* = 8.3 Hz, 2H), 7.16 (d, *J* = 8.5 Hz, 1H), 5.50 (s, 2H), 4.19 - 4.12 (m, 1H), 3.12 - 3.07 (m, 1H), 2.92 (d, *J* = 4.8 Hz, 3H), 2.90 - 2.86 (m, 1H), 1.30 (s, 9H). ^13^C NMR (126 MHz, DMSO-*d*_6_) *δ* 173.44, 155.59, 155.51, 153.43, 149.41, 143.40, 141.08, 139.00, 134.98, 130.55, 121.79, 119.93, 118.22, 78.17, 54.96, 38.21, 35.97, 28.16, 27.23. Purity: 96.6%. HRMS (ESI) *m/z* calcd for C_23_H_27_ClN_9_O_4_ [M + H]^+^: 528.1869, found: 528.1868.

**3'-(4-((2-Chloro-6-(methylamino)-9*H*-purin-9-yl)methyl)-1*H*-1,2,3-triazol-1-yl)-2'-hydroxy-[1,1'-biphenyl]-3-carboxylic acid (LC-YD02).** Yellow powder. 54% yield. ^1^H NMR (500 MHz, DMSO-*d*_6_) *δ* 12.98 (brs, 1H), 9.64 (brs, 1H), 8.57 (s, 1H), 8.24 (s, 1H), 8.23 (brs, 1H), 8.14 (t, *J* = 1.8 Hz, 1H), 7.95 (dt, *J* = 7.7, 1.3 Hz, 1H), 7.78 (dt, *J* = 7.7, 1.3 Hz, 1H), 7.59 (t, *J* = 7.7 Hz, 1H), 7.52 (dd, *J* = 7.9, 1.7 Hz, 1H), 7.45 (dd, *J* = 7.7, 1.7 Hz, 1H), 7.14 (t, *J* = 7.9 Hz, 1H), 5.52 (s, 2H), 2.92 (d, *J* = 4.6 Hz, 3H). ^13^C NMR (151 MHz, DMSO-*d*_6_) *δ* 167.39, 155.55, 153.37, 149.39, 147.38, 142.05, 141.07, 137.71, 133.40, 131.47, 131.22, 130.14, 128.65, 128.20, 126.91, 125.61, 125.38, 120.40, 118.14, 38.07, 27.19. Purity: 98.7%. HRMS (ESI) *m/z* calcd for C_22_H_18_ClN_8_O_3_ [M + H]^+^: 477.1185, found: 477.1186.

**2-(3-(4-((2-Chloro-6-(methylamino)-9*H*-purin-9-yl)methyl)-1*H*-1,2,3-triazol-1-yl)phenyl)acetic acid (LC-YD03).** White powder. 58% yield. ^1^H NMR (500 MHz, DMSO-*d*_6_) *δ* 12.43 (brs, 1H), 8.77 (s, 1H), 8.25 (brs, 2H), 7.81 (t, *J* = 2.0 Hz, 1H), 7.75 (dd, *J* = 8.0, 1.7 Hz, 1H), 7.52 (t, *J* = 7.7 Hz, 1H), 7.38 (d, *J* = 7.7 Hz, 1H), 5.51 (s, 2H), 3.70 (s, 2H), 2.92 (d, *J* = 4.7 Hz, 3H). ^13^C NMR (151 MHz, DMSO-*d*_6_) *δ* 172.23, 155.60, 153.45, 149.63, 143.51, 141.17, 137.21, 136.40, 129.97, 129.71, 121.86, 121.20, 118.39, 38.24, 27.23. Purity: 99.3%. HRMS (ESI) *m/z* calcd for C_17_H_16_ClN_8_O_2_ [M + H]^+^: 399.1079, found: 399.1080.

**Methyl 2-(4-((2-chloro-6-(methylamino)-9*H*-purin-9-yl)methyl)-1*H*-1,2,3-triazol-1-yl)-2,3-dihydro-1*H*-indene-2-carboxylate (LC-YD04).** White powder. 88% yield. ^1^H NMR (500 MHz, DMSO-*d*_6_) *δ* 8.21 (brs, 1H), 8.16 (brs, 1H), 8.13 (brs, 1H), 7.18 - 7.14 (m, 2H), 7.10 - 7.05 (m, 2H), 5.33 (s, 2H), 3.86 (s, 1H), 3.83 (s, 1H), 3.67 (s, 1H), 3.64 (s, 1H), 2.91 (d, *J* = 4.6 Hz, 3H), 2.54 (s, 3H). ^13^C NMR (151 MHz, DMSO-*d*_6_) *δ* 171.33, 155.52, 153.31, 149.25, 141.03, 140.96, 140.90, 126.29, 124.02, 123.58, 118.04, 76.80, 43.69, 38.18, 27.17. Purity: 97.9%. HRMS (ESI) *m/z* calcd for C_20_H_20_ClN_8_O_2_ [M + H]^+^: 439.1392, found: 439.1391.

**(*S*)-2-(4-((2-chloro-6-(methylamino)-9*H*-purin-9-yl)methyl)-1*H*-1,2,3-triazol-1-yl)-2-(naphthalen-1-yl)acetic acid (LC-YD05).** White powder. 84% yield. ^1^H NMR (500 MHz, DMSO-*d*_6_) *δ* 8.20 (d, *J* = 5.2 Hz, 1H), 8.13 (s, 1H), 8.10 (s, 1H), 8.09 - 7.97 (m, 3H), 7.61(brs, 2H), 7.57 - 7.54 (m, 2H), 7.45 (s, 1H), 5.35 (s, 2H), 2.90 (d, *J* = 4.6 Hz, 3H). (The proton of carboxyl was not detected). ^13^C NMR (151 MHz, DMSO-*d*_6_) *δ* 169.51, 155.51, 153.33, 149.28, 141.84, 140.99, 133.54, 130.85, 130.01, 129.94, 128.89, 127.18, 126.30, 125.51, 124.05, 122.86, 118.10, 62.73, 38.08, 27.18. Purity: 98.9%. HRMS (ESI) *m/z* calcd for C_21_H_18_ClN_8_O_2_ [M + H]^+^: 449.1236, found: 449.1235.

**(*S*)-3-(benzyloxy)-2-(4-((2-chloro-6-(methylamino)-9*H*-purin-9-yl)methyl)-1*H*-1,2,3-triazol-1-yl)propanoic acid (P21-B4).** White powder. 72% yield. ^1^H NMR (500 MHz, DMSO-*d*_6_) *δ* 8.24 (brs, 1H), 8.21 (brs, 1H), 8.17 (s, 1H), 7.28 - 7.24 (m, 3H), 7.15 - 7.13 (m, 2H), 5.81 (dd, *J* = 7.1, 3.3 Hz, 1H), 5.46 (s, 2H), 4.47 (q, *J* = 12.0 Hz, 2H), 4.16 (dd, *J* = 10.6, 7.1 Hz, 1H), 3.95 (dd, *J* = 10.6, 3.3 Hz, 1H), 2.92 (d, *J* = 4.6 Hz, 3H). (The proton of carboxyl was not detected). ^13^C NMR (151 MHz, DMSO-*d*_6_) *δ* 168.28, 155.55, 153.42, 149.30, 142.06, 140.99, 137.48, 128.25, 127.60, 127.49, 124.17, 118.16, 72.16, 68.70, 62.17, 38.18, 27.20. Purity: 98.3%. HRMS (ESI) *m/z* calcd for C_19_H_20_ClN_8_O_3_ [M + H]^+^: 443.1341, found: 443.1343.

**2-(4-((2-Chloro-6-(methylamino)-9*H*-purin-9-yl)methyl)-1*H*-1,2,3-triazol-1-yl)-5-hydroxybenzoic acid (P21-B8).** Brown powder. 61% yield. ^1^H NMR (500 MHz, DMSO-*d*_6_) *δ* 13.05 (brs, 1H), 10.40 (s, 1H), 8.41 (s, 1H), 8.22 (brs, 1H), 8.15 (s, 1H), 7.38 (d, *J* = 8.6 Hz, 1H), 7.29 (d, *J* = 2.8 Hz, 1H), 7.06 (dd, *J* = 8.6, 2.8 Hz, 1H), 5.47 (s, 2H), 2.93 (d, *J* = 4.5 Hz, 3H). ^13^C NMR (126 MHz, DMSO-*d*_6_) *δ* 166.09, 158.46, 155.58, 153.43, 149.41, 141.62, 140.86, 129.71, 128.55, 127.07, 125.79, 118.72, 118.16, 116.71, 38.07, 27.23. Purity: 95.0%. HRMS (ESI) *m/z* calcd for C_16_H_14_ClN_8_O_3_ [M + H]^+^: 401.0872, found: 401.0873.

**3-(4-((2-Chloro-6-(methylamino)-9*H*-purin-9-yl)methyl)-1*H*-1,2,3-triazol-1-yl)-5-hydroxybenzoic acid (P21-B9).** Brown powder. 21% yield. ^1^H NMR (500 MHz, DMSO-*d*_6_) *δ* 10.54 (brs, 1H), 8.87 (s, 1H), 8.24 (brs, 2H), 7.81 (s, 1H), 7.50 (s, 1H), 7.42 (s, 1H), 5.50 (s, 2H), 2.92 (d, *J* = 4.6 Hz, 3H). (The proton of carboxyl was not detected). ^13^C NMR (151 MHz, DMSO-*d*_6_) *δ* 166.52, 158.70, 155.55, 153.35, 149.39, 143.48, 141.11, 137.41, 122.10, 118.16, 116.21, 111.01, 110.78, 38.13, 27.19. Purity: 97.0%. HRMS (ESI) *m/z* calcd for C_16_H_14_ClN_8_O_3_ [M + H]^+^: 401.0872, found: 401.0869.

**(*S*)-2-(4-((2-chloro-6-(methylamino)-9*H*-purin-9-yl)methyl)-1*H*-1,2,3-triazol-1-yl)-3-(thiophen-2-yl)propanoic acid (P21-C2).** White powder. 87% yield. ^1^H NMR (500 MHz, DMSO-*d*_6_) *δ* 13.71 (brs, 1H), 8.23 (brs, 1H), 8.15 (brs, 2H), 7.23 (d, *J* = 5.1 Hz, 1H), 6.79 (t, *J* = 3.5 Hz, 1H), 6.68 (d, *J* = 3.5 Hz, 1H), 5.64 (s, 1H), 5.40 (s, 2H), 3.71 (d, *J* = 6.6 Hz, 2H), 2.92 (d, *J* = 3.6 Hz, 3H). ^13^C NMR (126 MHz, DMSO-*d*_6_) *δ* 169.18, 155.55, 153.41, 149.30, 142.12, 140.88, 137.72, 126.76, 126.71, 125.08, 124.09, 118.21, 63.54, 38.24, 31.10, 27.21. Purity: 99.6%. HRMS (ESI) *m/z* calcd for C_16_H_16_ClN_8_O_2_S [M + H]^+^: 419.0800, found: 419.0799.

**(*S*)-2-(4-((2-chloro-6-(methylamino)-9*H*-purin-9-yl)methyl)-1*H*-1,2,3-triazol-1-yl)-2-phenylacetic acid (P21-C3).** White powder. 78% yield. ^1^H NMR (500 MHz, DMSO-*d*_6_) *δ* 13.76 (brs, 1H), 8.22 (brs, 1H), 8.18 (brs, 1H), 8.16 (s, 1H), 7.51 - 7.47 (m, 2H), 7.45 - 7.40 (m, 3H), 6.72 (s, 1H), 5.40 (s, 2H), 2.91 (d, *J* = 4.6 Hz, 3H). ^13^C NMR (126 MHz, DMSO-*d*_6_) *δ* 169.11, 155.57, 153.42, 149.33, 142.08, 141.06, 134.17, 129.19, 129.03, 128.83, 123.87, 118.17, 65.54, 38.15, 27.24. Purity: 99.8%. HRMS (ESI) *m/z* calcd for C_17_H_16_ClN_8_O_2_ [M + H]^+^: 399.1079, found: 399.1080.

**(*S*)-2-(4-((2-chloro-6-(methylamino)-9*H*-purin-9-yl)methyl)-1*H*-1,2,3-triazol-1-yl)-3-(2,4-difluorophenyl)propanoic acid (P21-D5).** White powder. 74% yield. ^1^H NMR (500 MHz, DMSO-*d*_6_) *δ* 13.67 (brs, 1H), 8.23 (brs, 1H), 8.17 (s, 1H), 8.09 (brs, 1H), 7.11 - 7.02 (m, 2H), 6.77 (td, *J* = 8.4, 2.1 Hz, 1H), 5.65 (dd, *J* = 10.9, 5.1 Hz, 1H), 5.37 (s, 2H), 3.58 - 3.51 (m, 1H), 3.47 - 3.40 (m, 1H), 2.92 (d, *J* = 4.4 Hz, 3H). ^13^C NMR (151 MHz, DMSO-*d*_6_) *δ* 169.23, 161.76 (dd, *J* = 117.8, 12.1 Hz), 160.12 (dd, *J* = 117.8, 12.1 Hz), 155.55, 153.39, 149.31, 142.17, 140.88, 132.23 (dd, *J* = 9.7, 5.8 Hz), 123.90, 119.25 (dd, *J* = 10.7, 3.1Hz), 118.23, 111.14 (dd, *J* = 21.0, 3.5 Hz), 103.63 (t, *J* = 26.0 Hz), 62.02, 38.18, 30.00, 27.20. ^19^F NMR (471 MHz, DMSO-*d*_6_) *δ* -111.50 (d, *J* = 7.1 Hz), -113.39 (d, *J* = 7.1 Hz). Purity: 98.0%. HRMS (ESI) *m/z* calcd for C_18_H_16_ClF_2_N_8_O_2_ [M + H]^+^: 449.1047, found: 449.1046.

**(*S*)-2-(4-((2-chloro-6-(methylamino)-9*H*-purin-9-yl)methyl)-1*H*-1,2,3-triazol-1-yl)-2-cyclohexylacetic acid (P21-E4).** White powder. 90% yield. ^1^H NMR (500 MHz, DMSO-*d*_6_) *δ* 13.13 (brs, 1H), 8.23 (s, 1H), 8.21 (brs, 2H), 5.44 (s, 2H), 5.15 (d, *J* = 8.3 Hz, 1H), 2.92 (d, *J* = 4.6 Hz, 3H), 2.22 - 2.12 (m, 1H), 1.76 - 1.65 (m, 2H), 1.63 - 1.53 (m, 2H), 1.29 - 1.18 (m, 2H), 1.10 - 0.84 (m, 4H). ^13^C NMR (126 MHz, DMSO-*d*_6_) *δ* 169.49, 155.57, 153.38, 149.47, 142.05, 141.12, 123.82, 118.29, 67.40, 38.32, 29.15, 28.14, 27.21, 25.46, 25.17, 25.13. Purity: 99.9%. HRMS (ESI) *m/z* calcd for C_17_H_22_ClN_8_O_2_ [M + H]^+^: 405.1549, found: 405.1548.

**(*S*)-2-(4-((2-chloro-6-(methylamino)-9*H*-purin-9-yl)methyl)-1*H*-1,2,3-triazol-1-yl)-4-phenylbutanoic acid (P21-E7).** White powder. 85% yield. ^1^H NMR (500 MHz, DMSO-*d*_6_) *δ* 13.47 (brs, 1H), 8.27 (s, 1H), 8.22 (brs, 2H), 7.25 (t, *J* = 7.4 Hz, 2H), 7.17 (t, *J* = 7.4 Hz, 1H), 7.09 (d, *J* = 7.4 Hz, 2H), 5.45 (s, 2H), 5.29 (t, *J* = 7.0 Hz, 1H), 2.92 (d, *J* = 4.5 Hz, 3H), 2.47 - 2.41 (m, 3H), 2.40 - 2.33 (m, 1H). ^13^C NMR (126 MHz, DMSO-*d*_6_) *δ* 170.15, 155.55, 153.39, 149.37, 142.23, 141.05, 140.12, 128.39, 128.33, 126.13, 123.65, 118.22, 61.91, 38.31, 32.73, 31.34, 27.19. Purity: 96.2%. HRMS (ESI) *m/z* calcd for C_19_H_20_ClN_8_O_2_ [M + H]^+^: 427.1392, found: 427.1394.

**(*S*)-2-(4-((2-chloro-6-(methylamino)-9*H*-purin-9-yl)methyl)-1*H*-1,2,3-triazol-1-yl)-3-(1*H*-indol-3-yl)propanoic acid (P21-F4).** White powder. 87% yield. ^1^H NMR (500 MHz, DMSO-*d*_6_) *δ* 10.79 (s, 1H), 8.24 (s, 1H), 8.22 (brs, 1H), 8.07 (brs, 1H), 7.38 (d, *J* = 8.1 Hz, 1H), 7.27 (d, *J* = 8.1 Hz, 1H), 7.01 (t, *J* = 7.4 Hz, 1H), 6.93 (s, 1H), 6.88 (t, *J* = 7.4 Hz, 1H), 5.66 - 5.62 (m, 1H), 5.36 (s, 2H), 3.65 - 3.61 (m, 2H), 2.93 (d, *J* = 4.6 Hz, 3H). (The proton of carboxyl was not detected). ^13^C NMR (126 MHz, DMSO-*d*_6_) *δ* 170.00, 155.57, 153.42, 149.32, 141.91, 135.94, 126.78, 123.73, 123.71, 121.06, 118.50, 118.17, 117.88, 111.46, 108.57, 63.39, 38.15, 27.24. Purity: 98.2%. HRMS (ESI) *m/z* calcd for C_20_H_19_ClN_9_O_2_ [M + H]^+^: 452.1345, found: 452.1342.

**(*S*)-2-(4-((2-chloro-6-(methylamino)-9*H*-purin-9-yl)methyl)-1*H*-1,2,3-triazol-1-yl)-3-(phenylthio)propanoic (P21-G4).** White powder. 72% yield. ^1^H NMR (500 MHz, DMSO-*d*_6_) *δ* 8.24 (brs, 1H), 8.22 (s, 1H), 8.17 (s, 1H), 7.29 - 7.25 (m, 4H), 7.22 - 7.18 (m, 1H), 5.47 (dd, *J* = 9.4, 5.2 Hz, 1H), 5.41 (s, 2H), 3.80 - 3.76 (m, 2H), 2.93 (d, *J* = 4.6 Hz, 3H). (The proton of carboxyl was not detected). ^13^C NMR (151 MHz, DMSO-*d*_6_) *δ* 168.61, 155.57, 153.43, 149.32, 142.16, 140.98, 133.96, 129.58, 129.17, 126.77, 124.07, 118.15, 61.80, 38.16, 34.71, 27.22. Purity: 96.7%. HRMS (ESI) *m/z* calcd for C_18_H_18_ClN_8_O_2_S [M + H]^+^: 445.0956, found: 445.0958.

**(*R*)-2-(4-((2-chloro-6-(methylamino)-9*H*-purin-9-yl)methyl)-1*H*-1,2,3-triazol-1-yl)-2-(2-chlorophenyl)acetic acid (P21-H9).** White powder. 62% yield. ^1^H NMR (500 MHz, DMSO-*d*_6_) *δ* 13.99 (brs, 1H), 8.26 - 8.17 (m, 3H), 7.57 - 7.53 (m, 1H), 7.50-7.37 (m, 3H), 6.89 (s, 1H), 5.42 (s, 2H), 2.91 (d, *J* = 4.8 Hz, 3H). ^13^C NMR (151 MHz, DMSO-*d*_6_) *δ* 168.23, 155.57, 153.38, 149.53, 142.02, 141.15, 133.62, 132.03, 131.02, 129.91, 127.85, 124.36, 118.32, 63.17, 38.19, 27.19. Purity: 98.1%. HRMS (ESI) *m/z* calcd for C_17_H_15_Cl_2_N_8_O_2_ [M + H]^+^: 433.0690, found: 443.0691.

**2-(4-((2-Chloro-6-(methylamino)-9*H*-purin-9-yl)methyl)-1*H*-1,2,3-triazol-1-yl)-2-(2-(trifluoromethyl)phenyl)acetic acid (P22-B2).** White powder. 78% yield. ^1^H NMR (500 MHz, DMSO-*d*_6_) *δ* 8.28 (s, 1H), 8.22 (brs, 1H), 8.19 (brs, 1H), 7.81 (dd, *J* = 12.3, 7.7 Hz, 2H), 7.72 (d, *J* = 7.8 Hz, 1H), 7.67 (t, *J* = 7.7 Hz, 1H), 6.77 (s, 1H), 5.42 (s, 2H), 2.91 (d, *J* = 3.7 Hz, 3H). (The proton of carboxyl was not detected). ^13^C NMR (126 MHz, DMSO-*d*_6_) *δ* 168.42, 155.57, 153.42, 149.38, 142.01, 141.05, 133.26, 131.73, 131.31, 130.03, 127.72 (q, *J* = 30.0 Hz), 126.44 (q, *J* = 5.0 Hz), 124.35, 123.96 (q, *J* = 275.1 Hz), 118.21, 61.94, 38.17, 27.22. ^19^F NMR (471 MHz, DMSO-*d*_6_) *δ* -57.41. Purity: 99.7%. HRMS (ESI) *m/z* calcd for C_18_H_15_ClF_3_N_8_O_2_ [M + H]^+^: 467.0953, found: 467.0954.

**3-(4-((2-Chloro-6-(methylamino)-9*H*-purin-9-yl)methyl)-1*H*-1,2,3-triazol-1-yl)-3-(2-chlorophenyl)propanoic acid (LC-YD06).** White powder. 83% yield. ^1^H NMR (500 MHz, DMSO-*d*_6_) *δ* 12.63 (brs, 1H), 8.31 (s, 1H), 8.21 (brs, 1H), 8.16 (s, 1H), 7.53 - 7.48 (m, 1H), 7.39 - 7.28 (m, 3H), 6.44 (dd, *J* = 9.5, 5.6 Hz, 1H), 5.40 (s, 2H), 3.51 (dd, *J* = 17.0, 9.5 Hz, 1H), 3.27 (dd, *J* = 17.0, 5.6 Hz, 1H), 2.91 (d, *J* = 4.6 Hz, 3H). ^13^C NMR (151 MHz, DMSO-*d*_6_) *δ* 170.62, 155.52, 153.34, 149.31, 142.40, 140.98, 135.78, 132.09, 130.25, 129.84, 128.07, 127.95, 123.52, 118.14, 57.18, 38.22, 38.15, 27.18. Purity: 99.2%. HRMS (ESI) *m/z* calcd for C_18_H_17_Cl_2_N_8_O_2_ [M + H]^+^: 447.0846, found: 447.0845.

**(*S*)-3-(3-bromophenyl)-3-(4-((2-chloro-6-(methylamino)-9*H*-purin-9-yl)methyl)-1*H*-1,2,3-triazol-1-yl)propanoic acid (P22-B10).** White powder. 79% yield. ^1^H NMR (500 MHz, DMSO-*d*_6_) *δ* 12.55 (brs, 1H), 8.28 (s, 1H), 8.24 - 8.19 (m, 1H), 8.16 (s, 1H), 7.62 (t, *J* = 1.8 Hz, 1H), 7.52 - 7.49 (m, 1H), 7.38 - 7.35 (m, 1H), 7.31 (t, *J* = 7.8 Hz, 1H), 6.12 (dd, *J* = 9.5, 5.9 Hz, 1H), 5.40 (s, 2H), 3.53 (dd, *J* = 16.9, 9.5 Hz, 1H), 3.29 (dd, *J* = 16.9, 9.5 Hz, 1H), 2.91 (d, *J* = 4.7 Hz, 3H). ^13^C NMR (126 MHz, DMSO-*d*_6_) *δ* 170.73, 155.55, 153.42, 149.32, 142.49, 141.44, 141.00, 131.33, 131.00, 129.89, 126.05, 123.22, 121.88, 118.15, 59.66, 38.78, 38.25, 27.21. Purity: 99.9%. HRMS (ESI) *m/z* calcd for C_18_H_17_BrClN_8_O_2_ [M + H]^+^: 491.0341, found: 493.0318.

**2-Chloro-*N*-methyl-9-((1-phenyl-1*H*-1,2,3-triazol-4-yl)methyl)-9*H*-purin-6-amine (LC-YD11).** White powder. 55% yield. ^1^H NMR (500 MHz, DMSO-*d*_6_) *δ* 8.80 (s, 1H), 8.32 - 8.17 (m, 2H), 7.89 - 7.86 (m, 2H), 7.61 - 7.56 (m, 2H), 7.51 - 7.47 (m, 1H), 5.51 (s, 2H), 2.92 (d, *J* = 4.6 Hz, 3H). ^13^C NMR (151 MHz, DMSO-*d*_6_) *δ* 155.54, 153.38, 149.37, 143.45, 141.05, 136.48, 129.87, 128.79, 121.85, 120.12, 118.18, 38.16, 27.19. Purity: 96.9%. HRMS (ESI) *m/z* calcd for C_15_H_14_ClN_8_ [M + H]^+^: 341.1024, found: 341.1023.

**2-Chloro-*N*-methyl-9-((1-(*m*-tolyl)-1*H*-1,2,3-triazol-4-yl)methyl)-9*H*-purin-6-amine (LC-YD12).** White powder. 73% yield. ^1^H NMR (500 MHz, DMSO-*d*_6_) *δ* 8.75 (s, 1H), 8.32 - 8.16 (m, 2H), 7.71 (s, 1H), 7.66 (dd, *J* = 8.0, 2.2 Hz, 1H), 7.46 (t, *J* = 7.8 Hz, 1H), 7.30 (d, *J* = 7.7 Hz, 1H), 5.51 (s, 2H), 2.92 (d, *J* = 4.6 Hz, 3H), 2.39 (s, 3H). ^13^C NMR (151 MHz, DMSO-*d*_6_) *δ* 155.55, 153.38, 149.37, 143.42, 141.06, 139.65, 136.44, 129.67, 129.37, 121.76, 120.52, 118.18, 117.20, 38.19, 27.19, 20.88. Purity: 95.0%. HRMS (ESI) *m/z* calcd for C_16_H_16_ClN_8_ [M + H]^+^: 355.1181, found: 355.1179.

**2-Chloro-*N*-methyl-9-((1-(3-(trifluoromethyl)phenyl)-1*H*-1,2,3-triazol-4-yl)methyl)-9*H*-purin-6-amine** (**LC-YD13**). White powder. 71% yield. ^1^H NMR (500 MHz, DMSO-*d*_6_) *δ* 8.96 (s, 1H), 8.32 - 8.18 (m, 4H), 7.88 - 7.81 (m, 2H), 5.53 (s, 2H), 2.92 (d, *J* = 4.6 Hz, 3H). ^13^C NMR (151 MHz, DMSO-*d*_6_) *δ* 155.56, 153.41, 149.38, 143.84, 141.06, 136.95, 131.31, 130.51 (q, *J* = 32.6 Hz), 125.33 (q, *J* = 3.9 Hz), 124.10, 123.56 (q, *J* = 272.7 Hz), 122.23, 118.18, 116.84 (q, *J* = 3.9 Hz) , 38.17, 27.20. ^19^F NMR (471 MHz, DMSO-*d*_6_) *δ* -61.21. Purity: 95.3%. HRMS (ESI) *m/z* calcd for C_16_H_13_ClF_3_N_8_ [M + H]^+^: 409.0898, found: 409.0896.

**Supplementary Scheme 4.** Synthesis of compound LC-YD07.

**2-(3-((2-Chloro-6-(methylamino)-9*H*-purin-9-yl)methyl)phenyl)acetic acid** (**LC-YD07**).

To a solution of **fragment YD** (100 mg, 0.54 mmol, 1.0 equiv.) in DMF (3 mL) were added 3‑(bromomethyl)phenylacetic acid (137 mg, 0.60 mmol, 1.1 equiv.) and Cs_2_CO_3_ (352 mg, 1.08 mmol, 2.0 equiv.). The reaction mixture was stirred at 80 °C for 4 h, then cooled to room temperature and diluted with water (10 mL). The resulting mixture was purified by C18 column chromatography to afford the desired product **LC-YD07** as a white solid (32 mg, 19% yield). ^1^H NMR (500 MHz, DMSO-*d*_6_) *δ* 12.31 (brs, 1H), 8.22 (s, 2H), 7.29 (t, *J* = 7.9 Hz, 1H), 7.21 - 7.17 (m, 2H), 7.11 (dt, *J* = 7.7, 1.5 Hz, 1H), 5.33 (s, 2H), 3.54 (s, 2H), 2.92 (d, *J* = 4.7 Hz, 3H). ^13^C NMR (151 MHz, DMSO-*d*_6_) *δ* 172.60, 155.62, 153.50, 149.53, 141.24, 136.76, 135.68, 129.01, 128.77, 128.33, 125.67, 118.30, 46.21, 40.63, 27.26. Purity: 99.3%. HRMS (ESI) m/z calcd for C_15_H_15_ClN_5_O_2_ [M + H]^+^: 332.0909, found: 332.0910.

**Supplementary Scheme 5.** Synthesis of compound LC-YD08/09.

**Methyl 2-(3-(4-(hydroxymethyl)thiazol-2-yl)phenyl)acetate (17a).**

To a solution of (3-(2-methoxy-2-oxoethyl)phenyl)boronic acid (**16**) (200 mg, 1.03 mmol, 1.0 equiv.) in 1,4-dioxane (3 mL) were added (2-bromothiazol-4-yl)methanol (240 mg, 1.24 mmol, 1.2 equiv.), Pd(dppf)Cl_2_·CH_2_Cl_2_ (42 mg, 0.05 mmol, 0.05 equiv.), and a solution of Na_2_CO_3_ (218 mg, 2.06 mmol, 2.0 equiv.) in H_2_O (1 mL). The mixture was degassed and purged with N_2_ three times, then stirred at 90 °C for 3 h. After cooling to room temperature, the solvent was removed *in vacuo*. The residue was diluted with water (10 mL) and extracted with EtOAc (3 ×5 mL). The combined organic layers were washed with brine (3 × 15 mL). The organic layer was dried over Na_2_SO_4_, filtered, and concentrated *in vacuo*. The residue was purified by flash column chromatography to afford the compound **17a** as a yellow liquid (146 mg, 48% yield). ^1^H NMR (500 MHz, DMSO-*d*_6_) *δ* 7.86 (t, *J* = 1.8 Hz, 1H), 7.81 (dt, *J* = 7.8, 1.5 Hz, 1H), 7.48 (t, *J* = 1.2 Hz, 1H), 7.45 (t, *J* = 7.7 Hz, 1H), 7.37 (dt, *J* = 7.6, 1.4 Hz, 1H), 5.39 (t, *J* = 5.7 Hz, 1H), 4.63 (dd, *J* = 5.7, 1.1 Hz, 2H), 3.80 (s, 2H), 3.63 (s, 3H). LC-MS (ESI): *m/z* 263.93 [M + H]^+^.

**Methyl 2-(3-(5-(hydroxymethyl)thiazol-2-yl)phenyl)acetate (17b).**

Similar procedure of **17a** was performed to give **17b** (112 mg, 41% yield) as a yellow liquid. ^1^H NMR (500 MHz, Chloroform-*d*) *δ* 7.86 (t, *J* = 1.9 Hz, 1H), 7.81 (dt, *J* = 7.6, 1.6 Hz, 1H), 7.70 (t, *J* = 0.9 Hz, 1H), 7.40 (t, *J* = 7.6 Hz, 1H), 7.35 (dt, *J* = 7.7, 1.6 Hz, 1H), 4.90 - 4.88 (m, 2H), 3.71 (s, 3H), 3.69 (s, 2H), 2.21 (t, *J* = 7.7 Hz, 1H). LC-MS (ESI): *m/z* 263.93 [M + H]^+^.

**Methyl 2-(3-(4-(bromomethyl)thiazol-2-yl)phenyl)acetate** (**18a**).

To a solution of compound **17a** (140 mg, 0.53 mmol, 1.0 equiv.) in anhydrous DCM (3 mL) at 0 °C was added a solution of PBr_3_ (150 µL, 1.60 mmol, 3.0 equiv.) in anhydrous DCM (1 mL) dropwise. The mixture was stirred for 1 h. After the completed consumption of the starting material, the mixture was concentrated *in vacuo*. The residue was purified by flash column chromatography to afford the compound **18a** as a colorless oily liquid (105 mg, 61% yield). LC-MS (ESI): *m/z* 325.89 [M + H]^+^.

**Methyl 2-(3-(5-(bromomethyl)thiazol-2-yl)phenyl)acetate (18b).**

Similar procedure of **18a** was performed to give **18b** (64 mg, 47% yield) as a colorless oily liquid. ^1^H NMR (500 MHz, Chloroform-*d*) *δ* 7.86 (t, *J* = 1.8 Hz, 1H), 7.81 (dt, *J* = 7.5, 1.7 Hz, 1H), 7.79 (s, 1H), 7.41 (t, *J* = 7.6 Hz, 1H), 7.37 (dt, *J* = 7.6, 1.6 Hz, 1H), 4.75 (s, 2H), 3.71 (s, 3H), 3.70 (s, 2H). LC-MS (ESI): *m/z* 325.90 [M + H]^+^.

**Methyl 2-(3-(4-((2-chloro-6-(methylamino)-9*H*-purin-9-yl)methyl)thiazol-2-yl)phenyl)acetate (19a).**

To a solution of compound **18a** (105 mg, 0.32 mmol, 1.0 equiv.) in DMF (5 mL) were added **fragment YD** (65 mg, 0.35 mmol, 1.1 equiv.) and K_2_CO_3_ (88 mg, 0.64 mmol, 2.0 equiv.). The mixture was stirred at room temperature overnight. The reaction was then diluted with water (20 mL), extracted with EtOAc (3 × 10 mL), and the combined organic layers were washed with brine (3 × 15 mL). The organic layer was dried over Na_2_SO_4_, filtered, and concentrated *in vacuo*. The residue was purified by flash column chromatography to afford the compound **19a** as a white solid (39 mg, 28% yield). LC-MS (ESI): *m/z* 429.1 [M + H]^+^.

**Methyl 2-(3-(5-((2-chloro-6-(methylamino)-9*H*-purin-9-yl)methyl)thiazol-2-yl)phenyl)acetate** (**19b**).

Similar procedure of **19a** was performed to give **19b** (27 mg, 29% yield) as a white solid. ^1^H NMR (500 MHz, DMSO-*d*_6_) *δ* 8.28 (s, 2H), 7.94 (s, 1H), 7.81 (s, 1H), 7.78 (d, *J* = 7.7 Hz, 1H), 7.43 (t, *J* = 7.7 Hz, 1H), 7.37 (d, *J* = 7.6 Hz, 1H), 5.66 (s, 2H), 3.78 (s, 2H), 3.62 (s, 3H), 2.92 (d, *J* = 4.7 Hz, 3H).

**2-(3-(4-((2-Chloro-6-(methylamino)-9*H*-purin-9-yl)methyl)thiazol-2-yl)phenyl)acetic acid (LC-YD08)**.

To a solution of the compound **19a** (39 mg) in EtOH (2 mL) was added aqueous NaOH (0.5 mL, 2.5 M). The mixture was stirred at room temperature for 2 h, then acidified with 1 M HCl (1.5 mL). The resulting precipitate was collected by filtration, washed with water, and dried *in vacuo* to afford **LC-YD08** as a white solid (20 mg, 54% yield). ^1^H NMR (500 MHz, DMSO-*d*_6_) *δ* 12.40 (brs, 1H), 8.27 (s, 1H), 8.24 (d, *J* = 4.8 Hz, 1H), 7.82 (t, *J* = 1.7 Hz, 1H), 7.78 (dt, *J* = 7.6, 1.5 Hz, 1H), 7.51 (s, 1H), 7.43 (t, *J* = 7.7 Hz, 1H), 7.37 (dt, *J* = 7.7, 1.4 Hz, 1H), 5.49 (s, 2H), 3.68 (s, 2H), 2.92 (d, *J* = 4.6 Hz, 3H). ^13^C NMR (151 MHz, DMSO-*d*_6_) *δ* 172.47, 167.75, 155.55, 153.38, 151.95, 149.43, 141.30, 136.25, 132.65, 131.62, 129.20, 127.06, 124.51, 118.16, 117.63, 42.82, 40.27, 27.19. Purity: 98.7%. HRMS (ESI) *m/z* calcd for C_18_H_16_ClN_6_O_2_S [M + H]^+^: 415.0738, found: 415.0735.

**2-(3-(5-((2-Chloro-6-(methylamino)-9*H*-purin-9-yl)methyl)thiazol-2-yl)phenyl)acetic acid** (**LC-YD09**).

Similar procedure of **LC-YD08** was performed to give **LC-YD09** (16 mg, 66% yield) as a white solid. ^1^H NMR (500 MHz, DMSO-*d*_6_) *δ* 12.39 (brs, 1H), 8.28 (s, 2H), 7.93 (s, 1H), 7.80 (s, 1H), 7.76 (d, *J* = 7.8 Hz, 1H), 7.41 (t, *J* = 7.6 Hz, 1H), 7.36 (d, *J* = 7.7 Hz, 1H), 5.65 (s, 2H), 3.66 (s, 2H), 2.92 (d, *J* = 4.6 Hz, 3H). ^13^C NMR (151 MHz, DMSO-*d*_6_) *δ* 172.46, 167.84, 155.53, 153.49, 149.13, 143.27, 140.64, 136.22, 133.93, 132.76, 131.61, 129.17, 127.10, 124.45, 118.23, 40.22, 38.79, 27.19. Purity: 95.1%. HRMS (ESI) *m/z* calcd for C_18_H_16_ClN_6_O_2_S [M + H]^+^: 415.0738, found: 415.0733.

**Supplementary Scheme 6** Synthesis of compound LC-YD10.

**9-(4-Bromobenzyl)-2-chloro-*N*-methyl-9*H*-purin-6-amine** (**20**).

To a solution of 1-bromo-4-(bromomethyl)benzene (1.020 g, 4.08 mmol, 1.5 equiv.) in DMF (6 mL) were added **fragment YD** (500 mg, 2.72 mmol, 1.0 equiv.) and K_2_CO_3_ (752 mg, 5.44 mmol, 2.0 equiv.). The mixture was stirred at room temperature overnight. The reaction was then diluted with water (20 mL), extracted with EtOAc (3 × 10 mL), and the combined organic layers were washed with brine (3 × 15 mL). The organic layer was dried over Na_2_SO_4_, filtered, and concentrated *in vacuo*. The residue was purified by flash column chromatography to afford the compound **20** as a white solid (570 mg, 59% yield). LC-MS (ESI): *m/z* 352.0 [M + H]^+^.

**Methyl 2-(4'-((2-chloro-6-(methylamino)-9*H*-purin-9-yl)methyl)-[1,1'-biphenyl]-3-yl)acetate** (**21**).

To a solution of the compound **20** (100 mg, 0.28 mmol, 1.1 equiv.) in 1,4-dioxane (3 mL) were added (3-(2-methoxy-2-oxoethyl)phenyl)boronic acid (50 mg, 0.26 mmol, 1.0 equiv.), Pd(dppf)Cl_2_·CH_2_Cl_2_ (11 mg, 0.01 mmol, 0.05 equiv.), and a solution of Na_2_CO_3_ (55 mg, 0.52 mmol, 2.0 equiv.) in H_2_O (1 mL). The mixture was degassed and purged with N_2_ three times, then stirred at 90 °C for 3 h. After cooling to room temperature, the solvent was removed *in vacuo*. The residue was diluted with water (10 mL) and extracted with EtOAc (3 ×5 mL). The combined organic layers were washed with brine (3 × 15 mL). The organic layer was dried over Na_2_SO_4_, filtered, and concentrated *in vacuo*. The residue was purified by flash column chromatography to afford the compound **21** as an orange solid (71 mg, 60% yield). LC-MS (ESI): *m/z* 422.08 [M + H]^+^.

**2-(4'-((2-Chloro-6-(methylamino)-9*H*-purin-9-yl)methyl)-[1,1'-biphenyl]-3-yl)acetic acid** (**LC-YD10**).

To a solution of the compound **21** (71 mg) in EtOH (2 mL) was added aqueous NaOH (0.5 mL, 2.5 M). The mixture was stirred at room temperature for 2 h, then acidified with 1 M HCl (1.5 mL). The resulting precipitate was collected by filtration, washed with water, and dried *in vacuo* to afford **LC-YD10** as a white solid (30 mg, 44% yield). ^1^H NMR (500 MHz, DMSO-*d*_6_) *δ* 12.35 (brs, 1H), 8.28 (s, 1H), 8.25 (d, *J* = 4.9 Hz, 1H), 7.64 - 7.62 (m, 2H), 7.53 - 7.49 (m, 2H), 7.40 - 7.35 (m, 3H), 7.25 (d, *J* = 7.6 Hz, 1H), 5.39 (s, 2H), 3.63 (s, 2H), 2.93 (d, *J* = 4.6 Hz, 3H). ^13^C NMR (151 MHz, DMSO-*d*_6_) *δ* 172.64, 155.56, 153.44, 149.47, 141.19, 139.59, 139.55, 135.91, 135.70, 128.84, 128.64, 127.99, 127.82, 127.03, 124.96, 118.29, 45.98, 40.62, 27.19. Purity: 96.5%. HRMS (ESI) *m/z* calcd for C_21_H_19_ClN_5_O_2_ [M + H]^+^: 408.1222, found: 408.1219.

**Supplementary Scheme 7.** Synthesis of compound LC-YD15.

**Methyl 2-(3-(4-((2-chloro-6-(methylamino)-9*H*-purin-9-yl)methyl)-1*H*-1,2,3-triazol-1-yl)phenyl)acetate** (**LC-YD15**).

To a solution of the **LC-YD03** (50 mg, 0.125 mmol, 1.0 equiv.) in MeOH (5 mL) at room temperature was added SOCl_2_ (18 µL, 0.25 mmol, 2.0 equiv.) dropwise. The mixture was then heated to 80 °C and stirred for 2 h. After cooling to room temperature, the solvent was removed under reduced pressure. The residue was washed with MeOH (3 × 5 mL), and the resulting solid was collected by filtration and dried *in vacuo* to afford the **LC-YD15** as a white powder (22 mg, 43% yield). ^1^H NMR (500 MHz, DMSO*-d*_6_) *δ* 8.77 (s, 1H), 8.27 (brs, 2H), 7.82 (t, *J* = 2.0 Hz, 1H), 7.77 (dd, *J* = 7.8, 2.2 Hz, 1H), 7.53 (t, *J* = 7.9 Hz, 1H), 7.39 (d, *J* = 7.6 Hz, 1H), 5.51 (s, 2H), 3.81 (s, 2H), 3.63 (s, 3H), 2.92 (d, *J* = 4.3 Hz, 3H). ^13^C NMR (151 MHz, DMSO-*d*_6_) *δ* 171.25, 155.52, 153.44, 149.37, 143.50, 141.07, 136.46, 136.45, 129.94, 129.86, 121.87, 121.18, 118.63, 51.87, 40.06, 38.21, 27.21. Purity: 97.0%. HRMS (ESI) *m/z* calcd for C_18_H_18_ClN_8_O_2_ [M + H]^+^: 413.1236, found: 413.1239.

**Supplementary Scheme 8.** Synthesis of compound LC-YD14.

**2-(3-(4-((2-Chloro-6-(methylamino)-9*H*-purin-9-yl)methyl)-1*H*-1,2,3-triazol-1-yl)phenyl)acetamide (LC-YD14).**

To a solution of **LC-YD03** (50 mg, 0.13 mmol, 1.0 equiv.) in DMF (3 mL) were added HATU (57 mg, 0.20 mmol, 1.5 equiv.) and DIPEA (45 µL, 0.26 mmol, 2.0 equiv.). The mixture was stirred for 5 min, and then ammonium chloride (11 mg, 0.20 mmol, 1.5 equiv.) was added to the reaction mixture. The reaction was stirred at room temperature overnight. The reaction mixture was diluted with water (15 mL) and extracted with EtOAc (3 × 10 mL). The combined organic layers were washed with brine (3 × 10 mL), dried over anhydrous Na_2_SO_4_, and concentrated *in vacuo* to afford the crude. The resulting crude was purified by column chromatography to afford the **LC-YD14** as a white solid (12 mg, 23% yield). ^1^H NMR (500 MHz, DMSO-*d*_6_) *δ* 8.76 (s, 1H), 8.25 (brs, 2H), 7.79 (t, *J* = 2.0 Hz, 1H), 7.71 (dd, *J* = 8.0, 2.0 Hz, 1H), 7.50 (t, *J* = 7.5 Hz, 1H), 7.36 (d, *J* = 7.5 Hz, 1H), 6.95 (s, 1H), 5.51 (s, 2H), 3.49 (s, 2H), 2.92 (d, *J* = 4.6 Hz, 3H). ^13^C NMR (151 MHz, DMSO-*d*_6_) *δ* 171.63, 155.56, 153.39, 149.39, 143.46, 141.08, 138.54, 136.34, 129.63, 129.57, 121.85, 120.87, 118.11, 41.83, 38.17, 27.20. Purity: 99.6%. HRMS (ESI) *m/z* calcd for C_17_H_17_ClN_9_O [M + H]^+^: 398.1239, found: 398.1241.

**Supplementary Scheme 9.** Synthesis of compound LC-YD16/17/18/19.

To a solution of **LC-YD03** (50 mg, 0.13 mmol, 1.0 equiv.) in DMF (3 mL) were added HATU (57 mg, 0.20 mmol, 1.5 equiv.) and DIPEA (45 µL, 0.26 mmol, 2.0 equiv.). The mixture was stirred for 5 min, and then the corresponding amine (0.20 mmol, 1.5 equiv.) was added to the reaction mixture. The reaction was stirred at room temperature overnight. The reaction mixture was diluted with water (15 mL) and extracted with EtOAc (3 × 10 mL). The combined organic layers were washed with brine (3 × 10 mL), dried over anhydrous Na_2_SO_4_, and concentrated *in vacuo* to afford the crude. The resulting crude was purified by column chromatography to yield the target compound.

**2-(3-(4-((2-Chloro-6-(methylamino)-9*H*-purin-9-yl)methyl)-1*H*-1,2,3-triazol-1-yl)phenyl)-*N*,*N*-dimethylacetamide (LC-YD16).** White powder. 33% yield. ^1^H NMR (500 MHz, DMSO-*d*_6_) *δ* 8.74 (s, 1H), 8.24 (brs, 2H), 7.74 - 7.68 (m, 2H), 7.50 (t, *J* = 7.5 Hz, 1H), 7.32 (d, *J* = 7.5 Hz, 1H), 5.50 (s, 2H), 3.79 (s, 2H), 3.03 (s, 3H), 2.92 (d, *J* = 4.6 Hz, 3H), 2.83 (s, 3H). ^13^C NMR (201 MHz, DMSO-*d*_6_) *δ* 169.82, 155.61, 153.45, 149.43, 143.50, 141.13, 138.28, 136.38, 129.87, 129.67, 121.88, 121.14, 118.16, 39.99, 38.22, 37.15, 35.12, 27.25. Purity: 96.5%. HRMS (ESI) *m/z* calcd for C_19_H_21_ClN_9_O [M + H]^+^: 426.1552, found: 426.1552.

**2-(3-(4-((2-Chloro-6-(methylamino)-9*H*-purin-9-yl)methyl)-1*H*-1,2,3-triazol-1-yl)phenyl)-1-(piperidin-1-yl)ethan-1-one** (**LC-YD17)**. White powder. 30% yield. ^1^H NMR (500 MHz, DMSO-*d*_6_) *δ* 8.76 (s, 1H), 8.25 (brs, 2H), 7.76 (t, *J* = 1.9 Hz, 1H), 7.74 - 7.70 (m, 1H), 7.50 (t, *J* = 7.9 Hz, 1H), 7.33 (dt, *J* = 7.7, 1.3 Hz, 1H), 5.51 (s, 2H), 3.80 (s, 2H), 3.48 - 3.40 (m, 4H), 2.92 (d, *J* = 4.6 Hz, 3H), 1.59 - 1.50 (m, 2H), 1.46 - 1.32 (m, 4H). ^13^C NMR (151 MHz, DMSO-*d*_6_) *δ* 168.41, 155.64, 154.17, 149.67, 143.70, 141.39, 138.82, 136.81, 130.14, 130.01, 122.38, 121.26, 118.50, 46.82, 42.64, 40.51, 38.87, 27.69, 26.43, 25.72, 24.40. Purity: 97.1%. HRMS (ESI) *m/z* calcd for C_22_H_25_ClN_9_O [M + H]^+^: 466.1865, found: 466.1870.

**2-(3-(4-((2-Chloro-6-(methylamino)-9*H*-purin-9-yl)methyl)-1*H*-1,2,3-triazol-1-yl)phenyl)-1-morpholinoethan-1-one** (**LC-YD18**). White powder. 12% yield. ^1^H NMR (500 MHz, DMSO-*d*_6_) *δ* 8.75 (s, 1H), 8.24 (brs, 2H), 7.76 - 7.70 (m, 2H), 7.51 (t, *J* = 7.8 Hz, 1H), 7.33 (d, *J* = 7.6 Hz, 1H), 5.51 (s, 2H), 3.83 (s, 2H), 3.56 - 3.50 (m, 6H), 3.48 - 3.42 (m, 2H), 2.92 (d, *J* = 4.6 Hz, 3H). ^13^C NMR (151 MHz, DMSO-*d*_6_) *δ* 168.62, 155.56, 153.39, 149.38, 143.48, 141.08, 138.03, 136.37, 129.70, 129.68, 121.81, 121.00, 118.14, 66.07, 45.82, 41.75, 40.06, 38.79, 27.20. Purity: 95.3%. HRMS (ESI) *m/z* calcd for C_21_H_23_ClN_9_O_2_ [M + H]^+^: 468.1658, found: 468.1660.

**2-(3-(4-((2-Chloro-6-(methylamino)-9*H*-purin-9-yl)methyl)-1*H*-1,2,3-triazol-1-yl)phenyl)-1-(piperazin-1-yl)ethan-1-one** (**LC-YD19**). White powder. 52% yield. ^1^H NMR (500 MHz, DMSO-*d*_6_) 8.76 (s, 1H), 8.25 (brs, 2H), 8.23 - 8.20 (m, 1H), 7.75 (s, 1H), 7.73 (d, *J* = 8.0 Hz, 1H), 7.51 (t, *J* = 7.8 Hz, 1H), 7.32 (d, *J* = 7.7 Hz, 1H), 5.51 (s, 2H), 3.84 (s, 2H), 3.64 - 3.50 (m, 4H), 2.92 (d, *J* = 4.6 Hz, 3H), 2.84 (s, 4H). ^13^C NMR (151 MHz, DMSO-*d*_6_) *δ* 168.98, 156.02, 153.85, 149.84, 143.95, 141.55, 138.44, 136.82, 130.18, 130.14, 122.27, 121.48, 118.61, 44.83, 44.62, 44.31, 40.71, 40.51, 38.63, 27.66. Purity: 97.4%. HRMS (ESI) *m/z* calcd for C_21_H_24_ClN_10_O [M + H]^+^: 467.1818, found: 467.1821.

**Spectrums of compound characterization**

**^1^H NMR, ^13^C NMR, HPLC, HRMS spectra of new compounds**

^1^H NMR spectrum of compound **YD-1**

^13^C NMR spectrum of compound **YD-1**

HPLC spectrum of compound **YD-1**.

HRMS Spectra of compound **YD-1**.

^1^H NMR spectrum of compound **YD-2.**

^13^C NMR spectrum of compound **YD-2.**

HPLC spectrum of compound **YD-2.**

HRMS spectrum of compound **YD-2.**

^1^H NMR spectrum of compound **YD-3.**

^13^C NMR spectrum of compound **YD-3.**

HPLC spectrum of compound **YD-3.**

HRMS spectrum of compound **YD-3.**

^1^H NMR spectrum of compound **LC-YD01.**

^13^C NMR spectrum of compound **LC-YD01.**

HPLC spectrum of compound **LC-YD01.**

HRMS spectrum of compound **LC-YD01.**

^1^H NMR spectrum of compound **P2-C11.**

^13^C NMR spectrum of compound **P2-C11.**

HPLC spectrum of compound **P2-C11.**

HRMS spectrum of compound **P2-C11.**

^1^H NMR spectrum of compound **P3-E2.**

^13^C NMR spectrum of compound **P3-E2.**

HPLC spectrum of compound **P3-E2.**

HRMS spectrum of compound **P3-E2.**

^1^H NMR spectrum of compound **LC-YD02.**

^13^C NMR spectrum of compound **LC-YD02.**

HPLC spectrum of compound **LC-YD02.**

HRMS spectrum of compound **LC-YD02.**

^1^H NMR spectrum of compound **LC-YD03.**

^13^C NMR spectrum of compound **LC-YD03.**

HPLC spectrum of compound **LC-YD03.**

HRMS spectrum of compound **LC-YD03.**

^1^H NMR spectrum of compound **LC-YD04.**

^13^C NMR spectrum of compound **LC-YD04.**

HPLC spectrum of compound **LC-YD04.**

HRMS spectrum of compound **LC-YD04.**

^1^H NMR spectrum of compound **LC-YD05.**

^13^C NMR spectrum of compound **LC-YD05.**

HPLC spectrum of compound **LC-YD05.**

HRMS spectrum of compound **LC-YD05.**

^1^H NMR spectrum of compound **P21-B4.**

^13^C NMR spectrum of compound **P21-B4.**

HPLC spectrum of compound **P21-B4.**

HRMS spectrum of compound **P21-B4.**

^1^H NMR spectrum of compound **P21-B8.**

^13^C NMR spectrum of compound **P21-B8.**

HPLC NMR spectrum of compound **P21-B8.**

HRMS NMR spectrum of compound **P21-B8.**

^1^H NMR spectrum of compound **P21-B9.**

^13^C NMR spectrum of compound **P21-B9.**

HPLC spectrum of compound **P21-B9.**

HRMS spectrum of compound **P21-B9.**

^1^H NMR spectrum of compound **P21-C2.**

^13^C NMR spectrum of compound **P21-C2.**

HPLC spectrum of compound **P21-C2.**

HRMS spectrum of compound **P21-C2.**

^1^H NMR spectrum of compound **P21-C3.**

^13^C NMR spectrum of compound **P21-C3.**

HPLC spectrum of compound **P21-C3.**

HRMS spectrum of compound **P21-C3.**

^1^H NMR spectrum of compound **P21-D5.**

^13^C NMR spectrum of compound **P21-D5.**

^19^F NMR spectrum of compound **P21-D5.**

HPLC spectrum of compound **P21-D5.**

HRMS spectrum of compound **P21-D5.**

^1^H NMR spectrum of compound **P21-E4.**

^13^C NMR spectrum of compound **P21-E4.**

HPLC spectrum of compound **P21-E4.**

HRMS spectrum of compound **P21-E4.**

^1^H NMR spectrum of compound **P21-E7.**

^13^C NMR spectrum of compound **P21-E7.**

HPLC spectrum of compound **P21-E7.**

HRMS spectrum of compound **P21-E7.**

^1^H NMR spectrum of compound **P21-F4.**

^13^C NMR spectrum of compound **P21-F4.**

HPLC spectrum of compound **P21-F4.**

HRMS spectrum of compound **P21-F4.**

^1^H NMR spectrum of compound **P21-G4.**

^13^C NMR spectrum of compound **P21-G4.**

HPLC spectrum of compound **P21-G4.**

HRMS spectrum of compound **P21-G4.**

^1^H NMR spectrum of compound **P21-H9.**

^13^C NMR spectrum of compound **P21-H9.**

HPLC spectrum of compound **P21-H9.**

HRMS spectrum of compound **P21-H9.**

^1^H NMR spectrum of compound **P22-B2.**

^13^C NMR spectrum of compound **P22-B2.**

^19^F NMR spectrum of compound **P22-B2.**

HPLC spectrum of compound **P22-B2.**

HRMS spectrum of compound **P22-B2.**

^1^H NMR spectrum of compound **LC-YD06.**

^13^C NMR spectrum of compound **LC-YD06.**

HPLC spectrum of compound **LC-YD06.**

HRMS spectrum of compound **LC-YD06.**

^1^H NMR spectrum of compound **P22-B10.**

^13^C NMR spectrum of compound **P22-B10.**

HPLC spectrum of compound **P22-B10.**

HRMS spectrum of compound **P22-B10.**

^1^H NMR spectrum of compound **12.**

^1^H NMR spectrum of compound **13.**

^1^H NMR spectrum of compound **15.**

^1^H NMR spectrum of compound **FP-YD.**

^13^C NMR spectrum of compound **FP-YD.**

HPLC spectrum of compound **FP-YD.**

HRMS spectrum of compound **FP-YD.**

^1^H NMR spectrum of compound **LC-YD07**.

^13^C NMR spectrum of compound **LC-YD07**.

HPLC spectrum of compound **LC-YD07**.

HRMS spectrum of compound **LC-YD07**.

^1^H NMR spectrum of compound **LC-YD08**.

^13^C NMR spectrum of compound **LC-YD08**.

HPLC spectrum of compound **LC-YD08**.

HRMS spectrum of compound **LC-YD08**.

^1^H NMR spectrum of compound **LC-YD09**.

^13^C NMR spectrum of compound **LC-YD09**.

HPLC spectrum of compound **LC-YD09**.

HRMS spectrum of compound **LC-YD09**.

^1^H NMR spectrum of compound **LC-YD10**.

^13^C NMR spectrum of compound **LC-YD10**.

HPLC spectrum of compound **LC-YD10**.

HRMS spectrum of compound **LC-YD10**.

^1^H NMR spectrum of compound **LC-YD11**.

^13^C NMR spectrum of compound **LC-YD11**.

HPLC spectrum of compound **LC-YD11**.

HRMS spectrum of compound **LC-YD11**.

^1^H NMR spectrum of compound **LC-YD12**.

^13^C NMR spectrum of compound **LC-YD12**.

HPLC spectrum of compound **LC-YD12**.

HRMS spectrum of compound **LC-YD12**.

^1^H NMR spectrum of compound **LC-YD13**.

^13^C NMR spectrum of compound **LC-YD13**.

^19^F spectrum of compound **LC-YD13**.

HPLC spectrum of compound **LC-YD13**.

HRMS spectrum of compound **LC-YD13**.

^1^H NMR spectrum of compound **LC-YD14**.

^13^C NMR spectrum of compound **LC-YD14**.

HPLC spectrum of compound **LC-YD14**.

HRMS spectrum of compound **LC-YD14**.

^1^H NMR spectrum of compound **LC-YD15.**

^13^C NMR spectrum of compound **LC-YD15.**

HPLC spectrum of compound **LC-YD15.**

HRMS spectrum of compound **LC-YD15.**

^1^H NMR of spectrum of compound **LC-YD16**.

^13^C NMR spectrum of compound **LC-YD16**.

HPLC spectrum of compound **LC-YD16**.

HRMS spectrum of compound **LC-YD16**

^1^H NMR spectrum of compound **LC-YD17**.

^13^C NMR spectrum of compound **LC-YD17**.

HPLC spectrum of compound **LC-YD17**.

HRMS spectrum of compound **LC-YD17**.

^1^H NMR spectrum of compound **LC-YD18**.

^13^C NMR spectrum of compound **LC-YD18**.

HPLC spectrum of compound **LC-YD18**.

HRMS spectrum of compound **LC-YD18**.

^1^H NMR spectrum of compound **LC-YD19**.

^13^C NMR spectrum of compound **LC-YD19**.

HPLC spectrum of compound **LC-YD19**.

HRMS spectrum of compound **LC-YD19**.
